# Censored-Data-Aware Screening of Hazardous Metal(loid)s in Wild Mushrooms from Urban and Peri-Urban Green Spaces: Multielement Fingerprints and Adult Exposure Assessment

**DOI:** 10.3390/jox16040141

**Published:** 2026-07-30

**Authors:** Antonio Peña-Fernández, M. Carmen Lobo, M. Ángeles Peña Fernández, Guillermo Torrado Durán, Mark D. Evans, Tiziana Sgamma, Tomás Cámara-Pastor, Gurminderjeet S. Jagdev

**Affiliations:** 1 Area of Legal and Forensic Medicine, Department of Surgery, Medical and Social Sciences, Faculty of Medicine and Health Sciences, University of Alcalá, Ctra. Madrid-Barcelona Km. 33.600, 28871 Alcalá de Henares, Madrid, Spain; 2 Leicester School of Allied Health Sciences, De Montfort University, Leicester LE1 9BH, UK; 3 Departamento de Investigación Agroambiental, Madrid Institute for Rural, Agricultural and Food Research and Development (IMIDRA), Finca el Encín, Crta. Madrid-Barcelona Km. 38.2, 28800 Alcalá de Henares, Madrid, Spain; 4 Department of Biomedical Sciences, Faculty of Pharmacy, University of Alcalá, Crta. Madrid-Barcelona Km. 33.6, 28871 Alcalá de Henares, Madrid, Spain; 5 Scientific Computation Research Institute (SCRIUR), University of La Rioja, 26006 Logroño, La Rioja, Spain

**Keywords:** wild mushrooms, xenobiotics, metal(loid)s, cadmium, arsenic, lead, environmental exposure, metal burden, adult dietary screening, censored data

## Abstract

Wild mushrooms collected from urban and peri-urban green spaces may represent environmentally exposed biological matrices for hazardous metal(loid)s, but their interpretation is complicated by taxonomic heterogeneity, tissue partitioning and left-censored analytical data. This study evaluated As, Be, Cd, Co, Cr, Cu, Mn, Ni, Pb, V and Zn in wild mushrooms from Leicester/Leicestershire, UK, using a reconstructed fruiting-body-level dataset designed to avoid double counting paired cap/stem records. The central question was whether taxon- and tissue-aware multielement profiling alters screening-level exposure interpretation compared with a pooled edible-relevant summary. In total, 121 fruiting-body-level units were evaluated on a dry-weight basis. Cu, Mn and Zn were detected in all units, while Cd and Pb were detected in 117/121 and 106/121 units, respectively. V, Cr and Ni were detected in 84/121, 70/121 and 64/121 units, whereas As, Co and Be showed substantial censoring. Be was not interpreted distributionally. Central concentrations selected under the censored-data framework were 1.856 mg kg^−1^ for Cd, 71.142 mg kg^−1^ for Cu, 14.318 mg kg^−1^ for Mn, 3.874 mg kg^−1^ for Ni, 2.171 mg kg^−1^ for Pb, 0.104 mg kg^−1^ for V and 97.506 mg kg^−1^ for Zn. Taxon-level profiles showed marked heterogeneity, and paired tissue analysis in *Agaricus bitorquis* showed higher cap than stem concentrations for Cd, Cu, Pb and Zn. The primary PCA was restricted to well-detected elements (Cd, Cu, Mn, Pb, V and Zn), explaining 73.92% of total variance across the first three components. Sensitivity analyses showed that the overall PCA configuration remained stable across alternative treatments of censored observations and after exclusion of flagged extreme records, although some loading magnitudes showed moderate variation. Under the 300 g fresh-weight week^−1^ scenario, Cd represented 47.3% of the EFSA tolerable weekly intake of 2.5 µg kg^−1^ bw week^−1^ in the pooled edible-relevant population (*N* = 43) and 50.9% in *Agaricus bitorquis*; however, taxon-specific screening changed both the magnitude and ranking of the principal threshold-based drivers, with Cu becoming the leading driver for *Coprinopsis atramentaria* and *Marasmius oreades*. None of the primary or exploratory park-level soil–mushroom associations remained statistically significant after false-discovery-rate correction in the harmonised eight-park analysis. Overall, the findings partially support the working hypothesis: taxon- and tissue-aware profiling improved screening-level interpretation, whereas park-level soil context did not provide robust evidence of prediction or transfer. The fruiting-body-level, censored-data-aware framework provides a transparent basis for exposure screening, although uncertainties related to speciation, bioaccessibility, moisture assumptions and analytical interferences remain.

## 1. Introduction

Wild mushrooms are environmentally exposed biological matrices that may concentrate hazardous metal(loid)s from soil, litter, woody substrates and atmospheric deposition, thereby acting as potential dietary exposure vectors in urban and peri-urban settings. They are consumed for their flavour, seasonality and cultural value, and edible taxa may contribute protein, fibre, minerals, phenolics, polysaccharides and other fungal metabolites with potential health relevance [1,2,3]. Their mineral composition may include nutritionally relevant elements such as Cu, Mn and Zn, although the contribution of wild mushrooms to dietary mineral intake depends strongly on species, portion size, preparation, seasonality and consumption frequency [4,5]. This dual nutritional–toxicological role makes wild mushrooms relevant to environmental exposure assessment and screening-level toxicological interpretation, particularly when they are collected from urban and peri-urban green spaces.

The accumulation of metal(loid)s by mushrooms is widely recognised. Concentrations of As, Cd, Co, Cr, Cu, Mn, Ni, Pb, Zn and other elements, including Hg in some studies, can vary substantially among taxa, anatomical tissues and collection sites [5,6,7,8,9]. Metal accumulation is not simply proportional to total soil concentration. It may depend on fungal trophic strategy, substrate type, mycelial depth, fruiting-body development, soil pH, organic matter, metal speciation, atmospheric deposition and species-specific uptake, sequestration and binding mechanisms. This complexity is supported by studies showing that some wild edible fungi behave as bioaccumulators for Cd and Hg but as bioexcluders for Ni, Cr and Pb, while metal concentrations also differ between saprophytic and ectomycorrhizal taxa [10]. Consequently, pooled “mushroom” averages can obscure taxon-specific fingerprints that are more relevant for dietary interpretation and exposure screening.

The UK remains comparatively underrepresented in the wild-mushroom metal(loid) literature. Weeks et al. [11] provided an important UK multi-element survey of wild edible fungi and blackberries, reporting measurable concentrations of Pb, Cu, Cd, Hg, As and other elements in wild fungi. On a fresh-weight basis, their wild fungi showed median concentrations of 0.17 mg kg^−1^ for Cd, 0.22 mg kg^−1^ for Pb, 6.0 mg kg^−1^ for Cu, 11.4 mg kg^−1^ for Zn, 1.44 mg kg^−1^ for Mn and 0.109 mg kg^−1^ for As. That study remains a key UK comparator, but it did not combine fruiting-body-level reconstruction, taxon-level profiling, PCA-based multielement fingerprinting, explicit censored-data reporting, soil-context correlations and adult dietary screening. Additional UK datasets are therefore useful, particularly for urban and peri-urban green spaces where wild mushrooms may occur in environments influenced by legacy contamination, traffic, soil properties and local species composition.

The present work forms part of a broader Leicester/Leicestershire environmental chemistry framework. A related study using the same broad field system examined Cd, Cu and Zn in topsoil and wild mushrooms, focusing on spatial distribution, species/tissue accumulation and apparent soil-to-fungus transfer in urban and rural green spaces [12]. The Cd, Cu and Zn measurements derive from the same analytical campaign and are therefore not presented here as newly generated determinations. In the present study, these measurements were reprocessed together with eight additional elements within a fruiting-body-level, censored-data-aware framework addressing a distinct research question. A companion topsoil manuscript reports the wider multielement soil dataset, including the composite sampling design, soil physicochemical characterisation, geochemical interpretation, enrichment assessment and screening-level health calculations [13]. The present manuscript has a different focus. Rather than repeating a soil geochemistry or soil-to-fungus transfer assessment, it evaluates an 11-element mushroom-focused dataset comprising As, Be, Cd, Co, Cr, Cu, Mn, Ni, Pb, V and Zn. Soil data are retained only as environmental context through exploratory park-level correlations and are not used to infer mechanistic individual uptake. The precise data overlap and the analytical distinctions between the present and companion studies are summarised in Supplementary Table S1, Panel A.

A key methodological issue in mushroom datasets is the definition of the analytical unit. Caps and stems are often analysed separately because anatomical partitioning can affect dietary exposure. Several studies have reported higher concentrations of selected elements in caps than in stems, although tissue patterns are element- and species-dependent [10,14,15,16]. Recent elemental and isotopic work in bolete mushrooms has also shown uneven within-fruiting-body distribution of trace elements, with caps and spore-bearing tissues often containing higher concentrations than stems [17]. However, if paired cap and stem records are treated as independent whole mushrooms, the same fruiting body may be counted twice. Accordingly, the main descriptive and dietary-screening dataset was reconstructed at the fruiting-body level. Full details of the reconstruction procedure and its limitations are provided in Section 2.4.

Left-censored data are another important challenge in mushroom metal(loid) studies. Some elements are consistently quantifiable, whereas others may be predominantly below the limit of detection. In the present dataset, Cu, Mn and Zn were detected consistently, whereas Be was almost entirely censored. To reduce the bias associated with simple LoD substitution, primary distributional summaries were based on observed medians for uncensored groups or regression on order statistics for eligible censored groups, with detection frequency and censoring reported explicitly. Arithmetic and geometric means based on simple LoD substitution were not used for primary interpretation. The primary PCA was also restricted to six sufficiently detected elements, whereas broader-element PCAs were retained only as supplementary sensitivity or diagnostic analyses. This approach follows established recommendations for the transparent analysis of censored environmental and food-contaminant data [18,19].

Dietary exposure screening for wild mushrooms requires conservative but carefully qualified assumptions. Cd and Pb are toxicological priorities because wild mushrooms can accumulate these elements and because chronic exposure is associated with kidney toxicity, neurodevelopmental effects and other adverse outcomes [20,21,22]. Cd is particularly relevant for wild fungi because several taxa show efficient accumulation and because high seasonal consumption may contribute non-negligibly to weekly intake [15,16,23,24]. Pb requires a different interpretative framework because no simple nutritional threshold can be applied and because neurodevelopmental endpoints remain critical in food-risk assessment [21]. As also requires caution because total As does not identify inorganic As. Mushroom studies have shown that arsenic speciation can vary substantially among taxa, substrates and phylogenetic groups, with arsenobetaine and other organic As species predominating in some taxa, whereas inorganic As may represent a variable fraction in others [25,26,27,28,29,30,31]. In cultivated mushrooms, *Agaricus bisporus* and *Lentinula edodes* have also been shown to differ not only in total As concentration but also in the localisation of As within the fruiting body [28]. Consequently, treating total As as inorganic As is appropriate only as a conservative screening assumption, not as a definitive risk characterisation. Similarly, total Cr does not distinguish Cr(III), Cr(VI) or organically complexed chromium species, and any Cr(VI) approximation based on total Cr should be presented only as a precautionary screening convention and not as evidence of Cr(VI) occurrence [32,33].

The relationship between soil and mushroom metal(loid) concentrations also requires caution. Soil chemistry provides essential environmental context, but mushroom composition is influenced by fungal taxonomy, tissue, substrate biology, mycelial distribution, microsite conditions, atmospheric deposition and element-specific uptake or exclusion mechanisms. Experimental and market-based studies of cultivated mushrooms indicate that substrate composition can influence As, Pb and Cd transfer, but that risks and transfer patterns are species-, substrate- and serving-size dependent [31]. Recent foraged-mushroom evidence also indicates that mineral soils and substrates may be only moderate-to-weak predictors of element concentrations in sporocarps [34]. Therefore, in this study, soil–mushroom correlations were designed as exploratory park/area-level contextual analyses rather than as individual uptake or mechanistic transfer models.

This distinction is important because Cu, Mn and Zn may be interpreted partly as nutritionally relevant elements, whereas As, Be, Cd, Co, Cr, Ni, Pb and V require a more cautious toxicological or screening-oriented interpretation depending on detection frequency, chemical form, reference value availability and consumption assumptions.

Accordingly, the present study was designed to address a specific interpretative problem rather than simply to expand elemental coverage. We asked whether fruiting-body-level reconstruction, separation of edible-relevant from non-edible or uncertain taxa, and taxon-, tissue- and censored-data-aware multielement analysis would materially alter the identification and interpretation of screening-level exposure drivers compared with a pooled edible-relevant reference population. We hypothesised that taxon- and tissue-specific multielement profiles would reveal differences in the magnitude and ranking of exposure-relevant drivers that were obscured by the pooled reference population, while also identifying where broad multielement patterns were robust to censored-value treatment and where environmental-context associations should not be overinterpreted.

The aims of this study were to: (i) describe fruiting-body-level concentrations of As, Be, Cd, Co, Cr, Cu, Mn, Ni, Pb, V and Zn in wild mushrooms from Leicester/Leicestershire; (ii) evaluate taxon-level, tissue-level and spatial patterns without double counting paired cap/stem records in the main descriptive dataset; (iii) characterise multielement fingerprints using a primary PCA restricted to well-detected elements (Cd, Cu, Mn, Pb, V and Zn), with wider-element PCA retained only as supplementary sensitivity analysis; (iv) contextualise exploratory soil–mushroom and mushroom–soil physicochemical correlations at park/area level; and (v) perform adult dietary-exposure screening for edible and edible-with-caution taxa, using central concentration estimates and high-end sensitivity scenarios where sample size allowed, while retaining non-edible, toxic, uncertain-edibility and unidentified taxa separately as hypothetical-ingestion scenarios only.

## 2. Materials and Methods

### 2.1. Study Area, Sampling Framework and Analytical Design

**Figure 1:**
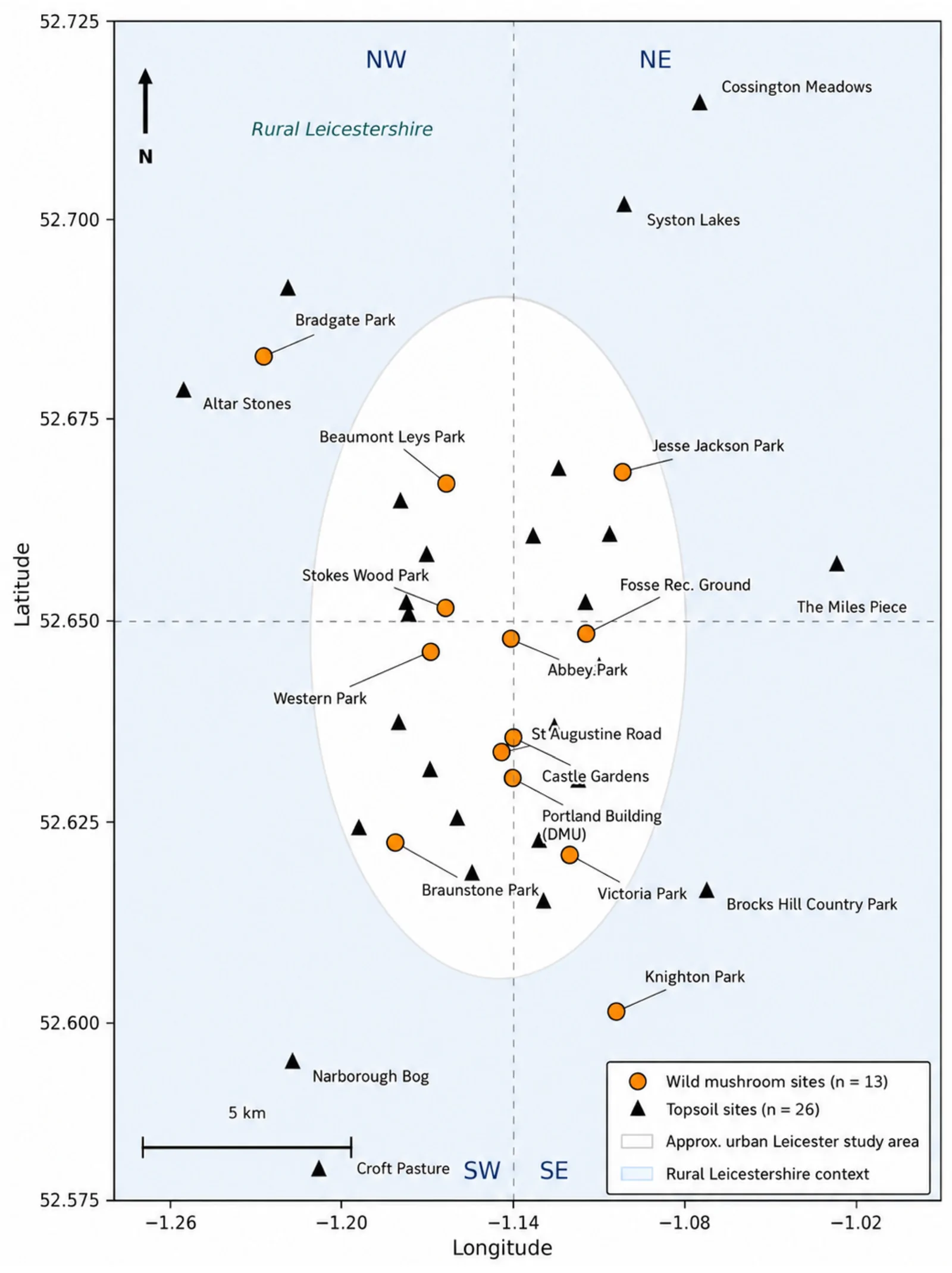
Sampling locations and coordinate-quadrant framework for the Leicester/Leicestershire wild mushroom and contextual topsoil datasets. Orange circles indicate wild mushroom collection sites included in the present multielement mushroom study (*n* = 13), and black triangles indicate the wider contextual topsoil sampling framework (*n* = 26 site-level composites). The approximate urban Leicester study area is shown in white, whereas the surrounding rural Leicestershire context is shown in light blue. Dashed horizontal and vertical lines define the NW, NE, SW and SE coordinate quadrants used for spatial grouping. The topsoil framework comprised 18 urban parks/open spaces and eight rural Leicestershire sites, with field increments pooled into homogenised site-level composites; these soil data are used here only as area-level environmental context, not as point-level evidence of individual mushroom uptake or statutory contaminated-land status.

Wild mushroom fruiting bodies were collected from urban green spaces in Leicester and from selected sites in Leicestershire, UK, within the same broad field framework used to characterise metal concentrations in regional topsoil and wild mushrooms (Figure 1; [12,13]). The present manuscript focuses on the mushroom multielement dataset and associated adult dietary-exposure screening. Soil data are used only as environmental context for exploratory park-level soil–mushroom and mushroom–soil physicochemical correlations.

The wider field design included 26 area-level topsoil composites, comprising 18 urban parks or open green spaces and eight rural Leicestershire sites. The full soil sampling design, composite preparation, geochemical interpretation and screening-level health assessment are reported in the companion topsoil study [13]. Wild mushroom fruiting bodies were collected opportunistically where naturally present. The mushroom dataset should therefore be interpreted as a field-based survey of available wild mushrooms rather than as a balanced taxonomic or county-wide inventory. The rural mushroom component was represented primarily by Bradgate Park and should not be interpreted as a pooled rural mushroom baseline for Leicestershire.

### 2.2. Wild Mushroom Collection, Labelling and Taxonomic Identification

Sampling was conducted between September and November 2019 under dry conditions, with ambient temperatures of approximately 8–17 °C. Mushrooms encountered naturally within each park or green-space area were collected opportunistically, and the geographical coordinates of each collection point were recorded using a handheld GPS device.

Nitrile gloves were worn during collection to reduce external contamination and avoid direct handling of potentially toxic or inedible taxa. Whenever practicable, intact fruiting bodies were removed with the stem attached to the upper soil or substrate. Individual specimens were placed in labelled, sealed polypropylene bags, transported to the laboratory and processed separately.

Preliminary taxonomic assignments were based on macroscopic morphology and were further assessed by DNA barcoding when suitable material was available. Genomic DNA was isolated from approximately 100 mg of frozen homogenised tissue using the DNeasy Plant Mini Kit^®^ (Qiagen Inc., Germantown, MD, USA). The fungal ribosomal internal transcribed spacer region was amplified with ITS1/ITS4 primers, sequenced and compared with reference sequences, consistent with the established use of ITS as a standard fungal barcode [35]. Specimens that could not be assigned confidently to species level were retained as “Unknown/Unidentified taxon” for analytical completeness but were not used for taxon-specific interpretation.

### 2.3. Mushroom Cleaning, Tissue Separation and Homogenisation

In the laboratory, visible debris, insects and adherent soil particles were manually removed from each fruiting body. Samples were washed with tap water, rinsed with deionised water and finally rinsed with Milli-Q ultrapure water produced using a Milli-Q^®^ Direct 8 system (Merck Millipore, Darmstadt, Germany; resistivity 18.2 MΩ·cm).

Where sufficient biomass was available, fruiting bodies were separated into cap and stem fractions before drying to allow tissue-specific evaluation. This approach is commonly used in mushroom trace-element studies because metal partitioning can differ between anatomical parts [10,14,15,16]. When biomass was limited, whole fruiting bodies were processed intact to prioritise taxon- and site-level analytical coverage.

Cleaned mushroom material was oven-dried at 50 °C until constant weight. The precise duration varied with sample size and initial moisture content, but drying was typically completed overnight. The dried samples were cryogenically homogenised with liquid nitrogen using a ceramic mortar and pestle to obtain a visually uniform powder. No sieve was used during mushroom sample preparation. The homogenisation equipment was thoroughly cleaned between samples to minimise cross-contamination. Processed samples were transferred to pre-cleaned capped polypropylene tubes and stored at −80 °C until mineralisation.

The ceramic mortar and pestle and other reusable sample-contact equipment were cleaned between samples using 5% *v*/*v* nitric acid, followed by thorough rinsing with deionised water and Milli-Q ultrapure water.

### 2.4. Fruiting-Body-Level Reconstruction for the Main Dataset

Because the original analytical dataset included whole fruiting bodies and tissue-separated cap/stem records, a fruiting-body-level dataset was reconstructed for the main descriptive and dietary-exposure analyses. Whole fruiting bodies were retained one-to-one, and paired cap/stem records were averaged without tissue-mass weighting as a pragmatic proxy for whole-fruiting-body composition. This reconstruction was used to avoid double counting of the same mushroom when both cap and stem had been analysed. Single-tissue records without a corresponding paired tissue were excluded from the main fruiting-body-level descriptive table but retained for supplementary tissue-specific summaries and visualisations. The final main descriptive dataset comprised 121 fruiting-body-level units. The taxon-level analytical inventory, including edibility category, tissue basis and area type, is provided in Supplementary Table S1, panel B.

Individual cap and stem dry masses were not available for all paired records; therefore, a true mass-weighted fruiting-body reconstruction could not be performed. The unweighted cap/stem average should therefore be interpreted as a pragmatic 50:50 reconstruction rather than as a measured biomass-weighted whole-fruiting-body concentration. To evaluate the uncertainty introduced by this assumption, a sensitivity analysis was performed for paired *Agaricus bitorquis* records using alternative cap:stem contribution scenarios of 30:70, 50:50 and 70:30. This taxon was selected because it provided the clearest paired cap/stem subset for evaluating tissue-related effects. The sensitivity analysis was used to assess the likely direction and magnitude of potential bias for elements showing tissue partitioning. For cap-enriched elements, a true cap contribution greater than 50% would mean that the unweighted 50:50 reconstruction underestimates the whole-fruiting-body concentration, whereas a true cap contribution below 50% would lead to overestimation. Conversely, for stem-enriched elements, the direction of bias would be reversed. These sensitivity analyses were used to support interpretation of the reconstruction approach but did not replace the primary fruiting-body-level dataset.

### 2.5. Mushroom Mineralisation and ICP-MS Determination

Where sufficient sample mass was available, approximately 0.500 g of dried homogenised mushroom material was weighed into pre-cleaned Teflon vessels and subjected to closed-vessel, non-microwave acid digestion. Concentrated nitric acid and hydrogen peroxide, both Suprapur^®^ grade (Merck, Darmstadt, Germany), were used for mineralisation. Hydrogen peroxide assisted oxidation of the organic matrix, in accordance with established wet-digestion procedures for biological and food samples [15,27]. Before use, the digestion vessels and other reusable equipment were washed with laboratory detergent, rinsed with tap and Milli-Q water, treated with 5% *v*/*v* nitric acid and thoroughly rinsed again.

Once digestion was complete and the vessels had cooled, the digestates were clarified using Whatman™ Grade 42 ashless quantitative filter paper (Cytiva, Marlborough, MA, USA) and made up to 25 mL with Milli-Q ultrapure water. A separate aliquot was subsequently passed through a 0.45 µm PTFE syringe filter (Agilent Technologies, Cheadle, Cheshire, UK) immediately before instrumental analysis. Paper filtration was therefore used for clarification and final-volume preparation, whereas PTFE filtration served as an additional measure to prevent residual particles from entering the ICP-MS sample-introduction system.

The concentrations of As, Be, Cd, Co, Cr, Cu, Mn, Ni, Pb, V and Zn were measured at De Montfort University using an Agilent 7900 ICP-MS with an integrated autosampler (Agilent Technologies, Santa Clara, CA, USA). The isotopes monitored were ^9^Be, ^51^V, ^52^Cr, ^55^Mn, ^59^Co, ^60^Ni, ^63^Cu, ^66^Zn, ^75^As, ^111^Cd and ^208^Pb. Instrumental settings were an RF power of 1548 W, an argon plasma gas flow of 13.9 L min^−1^, a nebuliser gas flow of 1.0 L min^−1^, an integration time of 0.3 s per point, three acquisitions of 50 s and pulse detector operation. Helium collision mode and ammonia reaction mode were not operational during the measurements.

### 2.6. Contextual Topsoil Dataset and Link with the Companion Soil Study

The soil dataset was used in the present manuscript only as environmental context for exploratory soil–mushroom and mushroom–soil physicochemical correlations. The full topsoil sampling design, composite preparation, pseudo-total digestion procedure, quality assurance/quality control, geochemical interpretation, enrichment assessment and screening-level human-health calculations are reported in the companion Leicester/Leicestershire topsoil manuscript [13].

The associated topsoil dataset was designed to characterise whole parks and green-space areas rather than the immediate microsite of each fruiting body. Briefly, that study was based on 850 surface-topsoil field increments collected across the Leicester/Leicestershire monitoring framework, comprising 18 urban parks/open spaces and eight rural Leicestershire sites. Field increments were pooled into 26 homogenised site-level composites, from which 102 analytical/subsampling observations were generated. Accordingly, the soil data represent area-level composite summaries rather than independent point-scale field samples.

Wild mushrooms were collected within these same monitored parks and green-space areas when naturally present. However, no dedicated surface-soil sample was collected immediately adjacent to each individual fruiting body at the time of mushroom collection. Therefore, the soil data provide robust park-/area-level environmental context, but they should not be interpreted as exact paired rhizosphere/substrate samples or as direct evidence of individual soil-to-mushroom uptake.

For the present mushroom-focused manuscript, the soil dataset was not used to calculate individual uptake coefficients or mechanistic soil-to-mushroom transfer factors. Instead, park-/area-level soil metal(loid) concentrations and physicochemical properties were used only to contextualise broad environmental patterns and explore whether mushroom multielement profiles were associated with site-level soil chemistry or soil properties. This distinction is important because mushroom fruiting bodies were collected opportunistically and reflect taxon occurrence, substrate, mycelial distribution and local microhabitat conditions, whereas the soil data were based on homogenised area-level composites. This contextual framing is consistent with evidence that substrate–mushroom relationships are taxon- and element-dependent and that soil or substrate concentrations should not be treated as direct predictors of individual fruiting-body composition [31,34].

Topsoil aliquots were digested using microwave-assisted acid digestion with 6 mL of nitric acid, 69%, and 2 mL of hydrochloric acid, 37%, both Suprapur^®^ grade (Merck, Darmstadt, Germany), in a Multiwave GO microwave digestion system (Anton Paar GmbH, Graz, Austria). After cooling, digestates were filtered through Whatman™ Grade 42 ashless quantitative filter paper and diluted to 50 mL with Milli-Q ultrapure water. Before ICP-MS analysis, mineralised samples were diluted in 5% *v*/*v* nitric acid and filtered through 0.45 µm syringe filters. The HNO_3_/HCl digestion was interpreted as an aqua-regia-like pseudo-total extraction of the acid-extractable/recoverable soil metal pool and not as complete silicate dissolution, as described in the companion topsoil study [13].

### 2.7. Soil Physicochemical Characterisation

Soil physicochemical properties and texture were determined in homogenised composite topsoil samples at IMIDRA, Madrid, Spain, using Spanish official soil analytical protocols [36]. The parameters measured were pH, electrical conductivity (EC), moisture content, organic matter (OM) and soil texture. These variables were included as contextual edaphic descriptors because pH, OM and texture can influence metal sorption/desorption, solubility, fine-particle retention and apparent soil-to-biota transfer [37,38]. They were not treated as independent point-level field measurements.

After air-drying and sieving through a 2 mm mesh, pH and EC were measured in soil–water suspensions using a 1:2.5 *w*/*v* ratio of topsoil to deionised water. Briefly, 10 g of homogenised topsoil was mixed with 25 mL of deionised water and agitated for 20 min before measurement with calibrated probes. Moisture content was determined gravimetrically after drying at 105 °C to constant weight. Organic matter was determined using the Walkley–Black dichromate oxidation method. Soil texture was determined on the homogenised <2 mm fraction using the Bouyoucos hydrometer method, allowing estimation of sand, silt and clay fractions [36].

### 2.8. Quality Assurance and Quality Control

Quality-control measures were implemented during sample handling, mineralisation, dilution, storage and ICP-MS measurement. Disposable polypropylene bags, tubes and syringe filters were used where appropriate. Reusable utensils and digestion vessels were washed with laboratory detergent, rinsed with deionised water, exposed to 5% *v*/*v* nitric acid and subsequently rinsed several times with Milli-Q ultrapure water. The mushroom mineralisation batches included 27 procedural acid blanks to assess background contributions introduced during preparation, digestion and dilution.

Element-specific method detection limits were calculated as 3.3 times the standard deviation of the procedural blank measurements, whereas limits of quantification were calculated using ten blank standard deviations. Concentrations were corrected for the corresponding blank contribution. Results below the relevant LoD were recorded as left-censored observations, and their frequency is reported in the descriptive tables.

Each digest was measured in triplicate, and the mean of the three ICP-MS determinations was used as the reported concentration. The isolated extreme records were acquired using the same procedure as the remainder of the dataset. However, the individual replicate values and sample-specific relative standard deviations were not available for retrospective assessment, and no targeted blank-before/blank-after carryover checks had been performed around those particular measurements. The records were therefore retained for analytical traceability but interpreted cautiously rather than as conclusive evidence of taxon-specific enrichment. Calibration standards were prepared from certified TraceCERT^®^ stock solutions (Merck, Darmstadt, Germany), and calibration functions were accepted when r^2^ was ≥0.99.

For mushroom analysis, NIST SRM 1570a Trace Elements in Spinach Leaves was used as a botanical reference material because no mushroom-specific certified reference material was available. This SRM is intended for evaluating analytical methods for major, minor and trace elements in botanical materials, agricultural food products and similar matrices (National Institute of Standards and Technology, NIST; Gaithersburg, MD, USA). Recoveries in SRM 1570a were 79.8% for V, 91.6% for Mn, 91.9% for Co, 120.2% for Ni, 102.4% for Cu, 97.5% for Zn, 104.4% for As, 89.7% for Cd and 120.0% for Pb. Most recoveries were within or close to the commonly accepted 80–120% range for trace-element analysis in environmental and biological matrices. V recovery was marginally below 80%, while Ni was marginally above 120%. The Pb value in SRM 1570a is an information value rather than a certified value and was therefore used only as an additional performance check. No certified Be or Cr value was available in SRM 1570a. However, the good recoveries obtained for Be and total Cr in the principal soil CRM, reported below, provided complementary evidence that major calibration or instrumental bias for these analytes was unlikely. Because this CRM was a soil rather than a botanical matrix and was processed using the soil digestion procedure, these results do not constitute matrix-matched validation of Be or Cr in mushrooms and cannot exclude fungal-matrix effects or polyatomic interferences, particularly for ^52^Cr.

For soil analysis, CRM059-50G Trace Metals–Loamy Clay 2, lot LRAA8361 (Sigma-Aldrich/Merck, Darmstadt, Germany; CRM certificate issued from Laramie, WY, USA), was used as the principal certified reference material. CRM059 is certified for multiple target elements on a dry-weight basis, including As, Be, Cd, Co, Cr, Cu, Mn, Ni, Pb, V and Zn. Recoveries for CRM059 were 102.8% for Be, 103.5% for V, 106.9% for Cr, 94.9% for Mn, 108.6% for Co, 102.1% for Ni, 106.3% for Cu, 104.6% for Zn, 106.3% for As, 105.2% for Cd and 99.2% for Pb. These results support the suitability of the soil digestion and ICP-MS workflow for contextual soil metal(loid) interpretation.

NCS DC87101 was also analysed as an additional soil reference material. Recoveries were 104.6% for As, 70.4% for Be, 100.3% for Co, 87.1% for Cr, 102.5% for Cu, 100.5% for Mn after conversion from MnO, 108.0% for Ni, 74.2% for Pb and 90.4% for V. Cadmium in NCS DC87101 was treated as reference-only, Fe was not directly reportable as elemental Fe because the reference value was oxide-based, and Zn recovery was low. Therefore, NCS DC87101 was used as supplementary QA/QC evidence for the soil dataset, whereas CRM059 was treated as the principal soil CRM for the target elements (China National Analysis Centre for Iron and Steel: Beijing, China). These soil QA/QC details are reported more fully in the companion topsoil study and are summarised here only because the present manuscript uses the soil dataset as environmental context [13].

Helium collision mode and ammonia reaction mode were not operational during the mushroom measurements. Instrumental performance was monitored using a tuning solution containing Ce, Li, Y, Co and Ti, with the instrument accepted for sample analysis at <5% RSD, together with external multi-element calibration, procedural blanks, triplicate acquisition and co-processed reference materials. These controls supported general instrumental and calibration performance but could not fully eliminate potential polyatomic interferences, particularly for ^52^Cr and, to a lesser extent, ^75^As and ^51^V. Although the HNO_3_/H_2_O_2_ digestion did not deliberately introduce chloride, matrix-derived carbon- and chlorine-based interferences cannot be completely excluded. Accordingly, Cr was interpreted with additional analytical caution and was excluded from the primary quantitative dietary screening, while As and V were retained with appropriate qualification. Total Cr concentrations were not considered evidence of Cr(VI) occurrence.

### 2.9. Data Treatment, Multivariate Analysis and Soil–Mushroom Correlations

Concentrations of As, Be, Cd, Co, Cr, Cu, Mn, Ni, Pb, V and Zn were expressed as mg kg^−1^ dry weight (dw). Values below the limit of detection (LoD) were treated as left-censored observations. At the reconstructed fruiting-body level, an element was considered detected when at least one of its original analytical component records was detected. Paired cap/stem units in which only one component was detected were classified as partially censored, whereas units with no detected component were classified as fully censored. Partially censored reconstructed values were retained as explicitly flagged working estimates, while fully censored units were treated as detection-limit-based observations and were not interpreted as measured concentrations.

Distributional summaries were selected using fixed operational criteria. For uncensored groups, the observed median and interquartile range were reported, and ROS was not applied. For censored groups, regression on order statistics was fitted using NADA::cenros [18,19,39] only when at least three observations were detected, censoring was <80%, the observed values and censoring limits were technically compatible with the procedure, and the fit converged without error. ROS estimates for groups with ≥50%, but <80% censoring were explicitly labelled exploratory and interpreted cautiously. Groups with fewer than three detections or ≥80% censoring were classified as not estimable and were described only by detection frequency, censoring percentage and detected range or maximum. Be, with only 1/121 detections, was therefore treated as not estimable/upper-bound only and excluded from biological interpretation.

LoD-capped values were used only as working inputs for procedures requiring a complete numerical dataset and were not interpreted as measured concentrations. P95 was treated exclusively as a high-end sensitivity estimate and was reported only when all of the following criteria were met: *n* ≥ 20, at least 10 detected observations, censoring <50%, availability of a valid observed or convergent ROS distribution, and 2000 valid nonparametric bootstrap estimates for calculation of its 95% confidence interval. When any of these criteria were not met, the detected range or maximum was reported instead of a P95. These rules were applied consistently to the overall, pooled edible-relevant, taxon, area, quadrant and tissue summaries.

For comparison with studies reporting fresh-weight concentrations, approximate fresh-weight equivalents were calculated using a dry-matter fraction of 0.10. These values were used only for contextual comparison and were not treated as moisture-specific measurements. The influence of alternative dry-matter fractions of 0.08 and 0.12 on the dietary-screening estimates was evaluated separately, as described in Section 2.10.

PCA was used to explore multielement fingerprints in the reconstructed fruiting-body-level dataset. The primary PCA was restricted to Cd, Cu, Mn, Pb, V and Zn and included all 121 fruiting-body-level units. These elements provided the most interpretable combination of detection frequency and analytical reliability. Concentrations were log10-transformed, centred and scaled to unit variance. The primary LoD-capped reconstruction was compared with alternative LoD/2 and ROS-based treatments of censored component records, applied before cap/stem reconstruction. Additional sensitivity analyses comprised 100 censored-value imputations, individual and joint exclusion of the flagged extreme records, and 2000 nonparametric bootstrap replicates. Similarity between the primary and sensitivity PCA score configurations was quantified using the Procrustes correlation coefficient, which measures concordance between configurations after optimal geometric alignment; values closer to 1 indicate greater similarity. Component signs were aligned with the primary solution before summarising bootstrap loadings. LoD-capped and imputed values were treated as working estimates rather than measured concentrations. A broader ten-element PCA excluding Be was retained as a supplementary sensitivity analysis, whereas the PCA including Be was diagnostic only because Be was almost entirely censored.

Before joining the mushroom and soil datasets, park names were standardised by removing extraneous spaces and reconciling verified naming variants, specifically Braunstone Park, Castle Garden/Castle Gardens and Stokeswood Park/Stokes Wood Park. This procedure produced a fixed overlap of eight parks: Abbey Park, Bradgate Park, Braunstone Park, Castle Gardens, Knighton Park, Stokes Wood Park, Victoria Park and Western Park. For each park and element, mushroom concentrations were summarised from the reconstructed fruiting-body-level units using the same observed-median or ROS criteria described above, whereas soil concentrations represented the corresponding homogenised site-level composite. The park-level mushroom summaries comprised 3–23 reconstructed fruiting-body units and one to eight taxa per park, and taxonomic composition was markedly unbalanced; for example, Bradgate Park was represented exclusively by *Coprinopsis atramentaria*, Victoria Park exclusively by *Mycena citrinomarginata*, whereas Abbey Park included eight taxa. The primary environmental panel comprised six same-element Spearman correlations for Cd, Cu, Mn, Pb, V and Zn. Spearman coefficients were accompanied by exact permutation *p*-values and Benjamini–Hochberg adjustment across the six primary tests [40]. Stability was examined using a leave-one-park-out analysis comprising eight omissions, with *n* = 7 parks in each repetition. A broader panel of same-element, cross-element and soil-physicochemical correlations was retained as exploratory supplementary analysis, with both within-family and global false-discovery-rate adjustments. Because the effective sample size was small, taxonomic composition differed among parks and the soil measurements represented homogenised park-level composites rather than substrate collected immediately adjacent to individual fruiting bodies, all associations were interpreted as contextual and not as evidence of prediction, mechanistic transfer or individual uptake.

The working hypothesis was evaluated through three complementary evidence blocks: comparison of taxon-specific dietary-screening estimates with a harmonised pooled edible-relevant reference population (*n* = 43); paired cap–stem analysis of 22 *Agaricus bitorquis* fruiting bodies; and assessment of the stability of the primary PCA under alternative censored-value and extreme-record treatments. Park-level soil–mushroom associations were evaluated separately as complementary environmental context and were not treated as a formal test of the working hypothesis.

Data processing and statistical analyses were conducted in R version 4.3.1 [41]. ROS estimation was performed using NADA version 1.6-1.1, following the approach described by Lee and Helsel [39].

### 2.10. Adult Dietary Exposure Screening

Adult dietary screening was performed for taxa retained in the edible-relevant screening block using two consumption scenarios: 100 and 300 g of fresh mushrooms week^−1^. Calculations assumed an adult body weight of 70 kg and a dry-matter fraction of 0.10. Because taxon-specific moisture content was not measured, additional sensitivity calculations were performed using dry-matter fractions of 0.08 and 0.12. The calculations assumed 100% bioaccessibility and should therefore be interpreted as screening estimates rather than estimates of absorbed dose.

The main screening scenario used the central concentration estimate selected according to the censored-data framework described in Section 2.9. For uncensored groups, the observed median was used. For censored groups with at least three detections and less than 80% censoring, the median estimated from the complete distribution modelled using regression on order statistics was used; estimates based on ≥50% censoring were explicitly treated as exploratory. No quantitative screening was performed when the criteria for central estimation were not met. A high-end concentration scenario was calculated only when the P95 met all eligibility criteria specified in Section 2.9. When these criteria were not met, detected ranges or maxima were reported descriptively rather than as P95 values.

The primary dietary-screening block included only reconstructed fruiting-body-level units from edible and edible-with-caution taxa. Single-tissue records for which a whole-fruiting-body-level unit could not be reconstructed were excluded from quantitative dietary screening. Edibility classifications were harmonised at taxon level before constructing the pooled edible-relevant reference population, which comprised 43 reconstructed fruiting-body-level units.

As and Pb were evaluated using margin-of-exposure approaches, for which lower MOE values indicate greater potential concern [21,42]. Total As was conservatively treated as inorganic As and compared with the EFSA reference point of 0.06 µg kg^−1^ bw day^−1^ [42]. This assumption was used for screening purposes only and does not indicate that inorganic As was measured or that all As occurred in inorganic form. Pb was evaluated against the EFSA BMDL01 for developmental neurotoxicity [21].

Cd exposure was expressed as a percentage of the EFSA tolerable weekly intake of 2.5 µg kg^−1^ bw week^−1^ [20]. Ni was compared with the EFSA tolerable daily intake of 13 µg kg^−1^ bw day^−1^ [43]. Cu, Mn, V and Zn were compared with the health-based or screening reference values reported in Supplementary Table S2 [44,45,46,47]. The comparator used for V was the ATSDR oral minimal risk level and not an EPA oral reference dose [46].

Be and Co were excluded from quantitative dietary screening. Be was detected in only one fruiting-body-level unit, precluding defensible exposure estimation, whereas Co was excluded because a sufficiently well-established toxicological benchmark for the intended screening application could not be confirmed.

Total Cr was excluded from the primary quantitative dietary screening because Cr speciation was not measured and total Cr cannot distinguish Cr(III), Cr(VI) or organically complexed Cr species [32,33]. A supplementary precautionary sensitivity analysis was performed using hypothetical fractions of 1%, 10%, 16.7% and 100% of total Cr as Cr(VI). The 16.7% scenario is mathematically equivalent to total Cr/6. These fractions were arbitrary and were not intended to represent measured or literature-derived Cr(VI) proportions. The scenarios were used solely to illustrate the dependence of Cr(VI)-equivalent estimates on unverified speciation assumptions and should not be interpreted as evidence that Cr(VI) was present in the mushroom samples.

Non-edible, toxic, uncertain-edibility and unidentified taxa were retained separately as hypothetical-ingestion scenarios only. These calculations do not constitute dietary-safety assessments because they do not account for intrinsic fungal toxins, psychoactive compounds, gastrointestinal toxicity, misidentification, preparation-related hazards or other non-metal risks. This distinction is particularly relevant to *Panaeolina foenisecii*, for which the available toxicological and pharmacological literature remains context-dependent [48,49].

## 3. Results

### 3.1. Overall Fruiting-Body-Level Metal(loid) Profile

The main descriptive dataset comprised 121 reconstructed fruiting-body-level units. Using the predefined fruiting-body-level detection rule, Cu, Mn and Zn were detected in all units, Cd in 117/121, Pb in 106/121, V in 84/121, Cr in 70/121 and Ni in 64/121. As and Co were detected in 37/121 and 34/121 units, respectively, corresponding to censoring of 69.4% and 71.9%. Be was detected in only one unit, corresponding to 99.2% censoring, and was therefore considered not estimable/upper-bound only.

The central estimates selected under the censored-data framework were 1.856 mg kg^−1^ for Cd, 1.356 mg kg^−1^ for Cr, 71.142 mg kg^−1^ for Cu, 14.318 mg kg^−1^ for Mn, 3.874 mg kg^−1^ for Ni, 2.171 mg kg^−1^ for Pb, 0.104 mg kg^−1^ for V and 97.506 mg kg^−1^ for Zn. Cu, Mn and Zn were represented by their observed medians, whereas the estimates for Cd, Cr, Ni, Pb and V were obtained from the complete distributions modelled using NADA::cenros. As and Co were retained only as high-censoring exploratory ROS summaries, and Be was not interpreted distributionally. Cr was retained for descriptive purposes, but Cr speciation was not measured, and Cr was excluded from the primary quantitative dietary screening. The complete overall fruiting-body-level summary is presented in Table 1.

Several concentration distributions were strongly right-skewed and included isolated high-concentration records. These observations were retained because they were traceable to individual sample identifiers, but upper-tail values were interpreted cautiously and were not treated as definitive evidence of biological enrichment.

Detections of As were unevenly distributed across taxa. Seventeen of the 37 detected fruiting-body-level units belonged to the Unknown/Unidentified taxon group. Total As was therefore interpreted cautiously as a signal requiring chemical speciation rather than as evidence of taxon-specific inorganic-As accumulation.

For contextual comparison with the UK dataset reported by Weeks et al. [11], application of the assumed dry-matter fraction of 0.10 produced approximate fresh-weight central estimates of 0.186 mg kg^−1^ for Cd, 0.217 mg kg^−1^ for Pb, 7.114 mg kg^−1^ for Cu and 9.751 mg kg^−1^ for Zn. These values were broadly comparable to the fresh-weight medians of 0.17, 0.22, 6.0 and 11.4 mg kg^−1^, respectively, reported by Weeks et al. [11]. The present values are approximate conversions and not moisture-specific measurements.

### 3.2. Taxon-Level Fruiting-Body Profiles

Taxon-level summaries, reported in Table 2, showed marked heterogeneity. Interpretation was based primarily on detection frequency, central estimates selected under the censored-data framework and detected ranges, rather than on arithmetic means that could be strongly influenced by isolated high-concentration observations.

*Agaricus bitorquis* (*N* = 22) showed relatively consistent concentrations for the well-detected elements. Observed median concentrations were 2.968 mg kg^−1^ for Cd, 105.603 mg kg^−1^ for Cu, 9.983 mg kg^−1^ for Mn, 2.082 mg kg^−1^ for Pb and 63.363 mg kg^−1^ for Zn. The ROS median for V was 0.083 mg kg^−1^. As was not detected; Co did not meet the criteria for central estimation, and Cr and Ni were retained only as high-censoring exploratory summaries.

*Coprinopsis atramentaria* (*N* = 8) had central estimates of 0.784 mg kg^−1^ for As, 0.183 mg kg^−1^ for Cd, 4.141 mg kg^−1^ for Cr, 80.824 mg kg^−1^ for Cu, 40.493 mg kg^−1^ for Mn, 10.872 mg kg^−1^ for Ni, 6.621 mg kg^−1^ for Pb, 0.422 mg kg^−1^ for V and 109.423 mg kg^−1^ for Zn. These results characterise the sampled taxon but should not be interpreted as a general rural baseline because all rural fruiting-body-level units belonged to this species.

*Marasmius oreades* (*N* = 5) had central estimates of 0.277 mg kg^−1^ for Cd, 62.809 mg kg^−1^ for Cu, 14.318 mg kg^−1^ for Mn, 1.251 mg kg^−1^ for Pb, 0.192 mg kg^−1^ for V and 76.631 mg kg^−1^ for Zn. As, Cr and Ni did not have sufficient detections for central estimation. Because of the small number of units, detected ranges and maxima were used descriptively, and no P95 values were reported.

*Mycena citrinomarginata* (*N* = 22) had central estimates of 0.556 mg kg^−1^ for Cd, 1.997 mg kg^−1^ for Cr, 50.781 mg kg^−1^ for Cu, 29.032 mg kg^−1^ for Mn, 11.796 mg kg^−1^ for Ni, 3.012 mg kg^−1^ for Pb, 0.211 mg kg^−1^ for V and 112.666 mg kg^−1^ for Zn. As did not meet the criteria for central estimation, whereas Co was retained only as a high-censoring exploratory summary. Isolated high Cu and Ni observations contributed to pronounced upper-tail variability in this taxon and were therefore interpreted cautiously.

*Panaeolina foenisecii* (*N* = 16) had central estimates of 2.566 mg kg^−1^ for Cd, 57.788 mg kg^−1^ for Cu, 162.809 mg kg^−1^ for Mn, 5.515 mg kg^−1^ for Ni, 3.895 mg kg^−1^ for Pb, 0.112 mg kg^−1^ for V and 86.031 mg kg^−1^ for Zn. As and Co did not meet the criteria for central estimation, whereas Cr was retained only as a high-censoring exploratory ROS summary.

*Pholiota adiposa* (*N* = 5) had observed median concentrations of 2.520 mg kg^−1^ for Cd, 60.954 mg kg^−1^ for Cu, 183.960 mg kg^−1^ for Mn and 84.631 mg kg^−1^ for Zn. This taxon included an isolated record with concentrations of 101.054 mg kg^−1^ for Cd, 2676.159 mg kg^−1^ for Mn and 3064.543 mg kg^−1^ for Zn. Because these maxima originated from a single fruiting-body record and the taxon-level sample size was small, they were retained descriptively but were not interpreted as definitive evidence of taxon-specific enrichment.

Overall, isolated upper-tail observations were retained in the analytical dataset because they were traceable to individual records, but conclusions were based primarily on censored-data-aware central estimates, detection frequencies and broader taxon-level patterns rather than on arithmetic means or individual maxima.

The Unknown/Unidentified taxon group (*N* = 17) had observed median concentrations of 1.941 mg kg^−1^ for As and 475.986 mg kg^−1^ for Zn. Because this analytical group lacked taxonomic resolution, its elemental profile was not interpreted as a species-specific biological pattern. The concentration of As detections in this group also supported cautious interpretation of total As and reinforced the need for chemical speciation.

**Figure 2:**
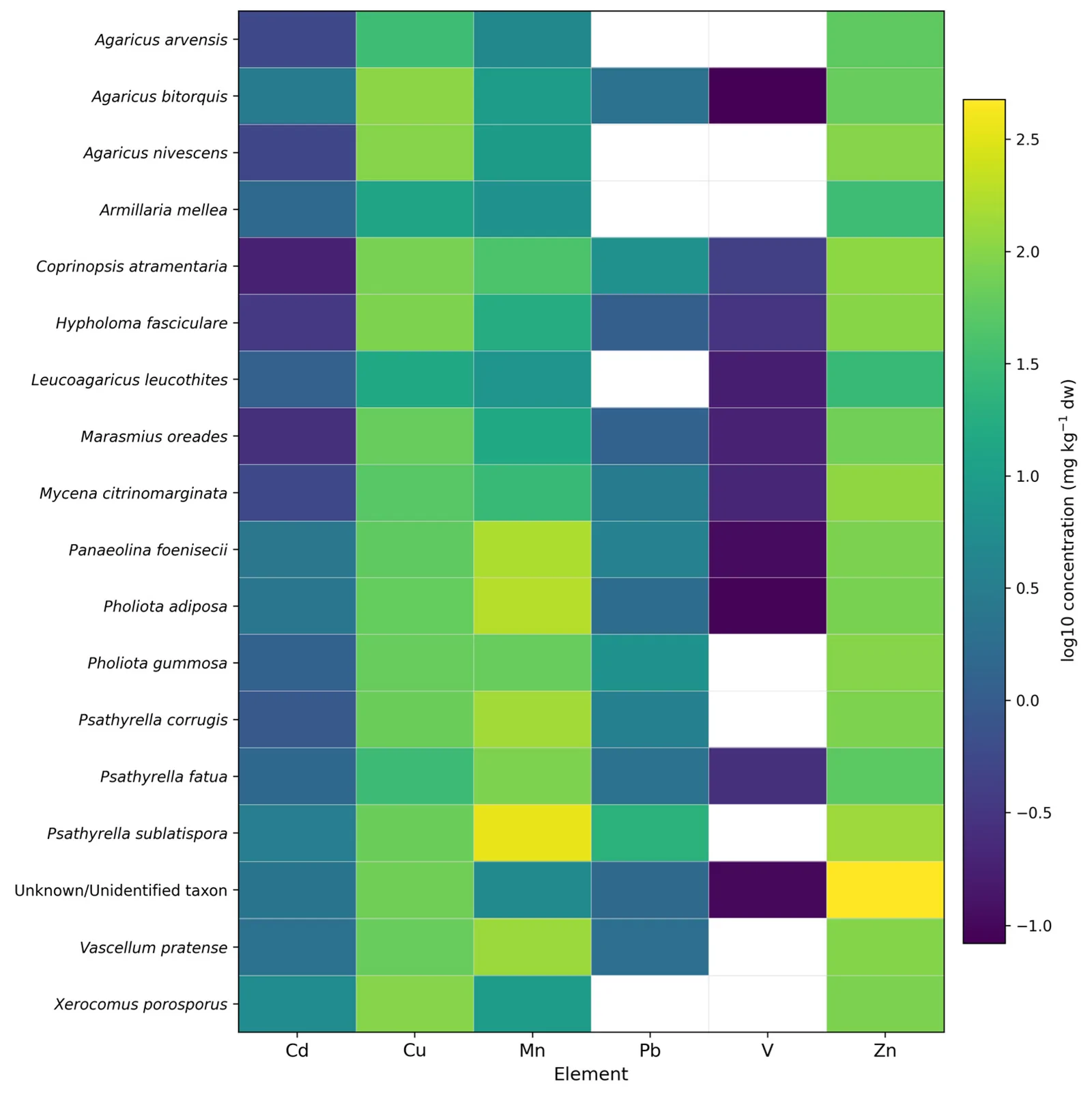
Taxon-level multielement heatmap for Cd, Cu, Mn, Pb, V and Zn in the reconstructed fruiting-body-level dataset. Colours represent log_10_-transformed central estimates selected under the censored-data framework. Blank cells indicate that a central estimate was not considered defensible because of insufficient detections. ROS-derived values represent modelled distributional summaries and should not be interpreted as directly measured concentrations for censored observations.

**Figure 3:**
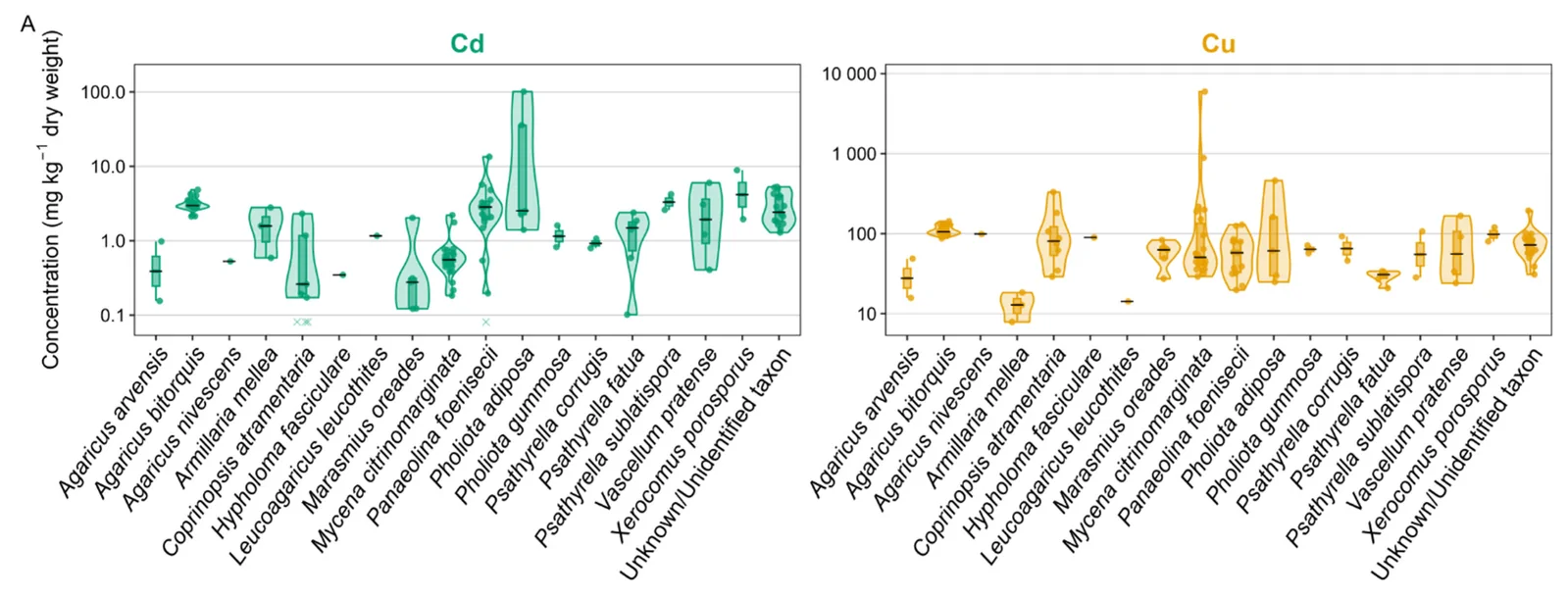

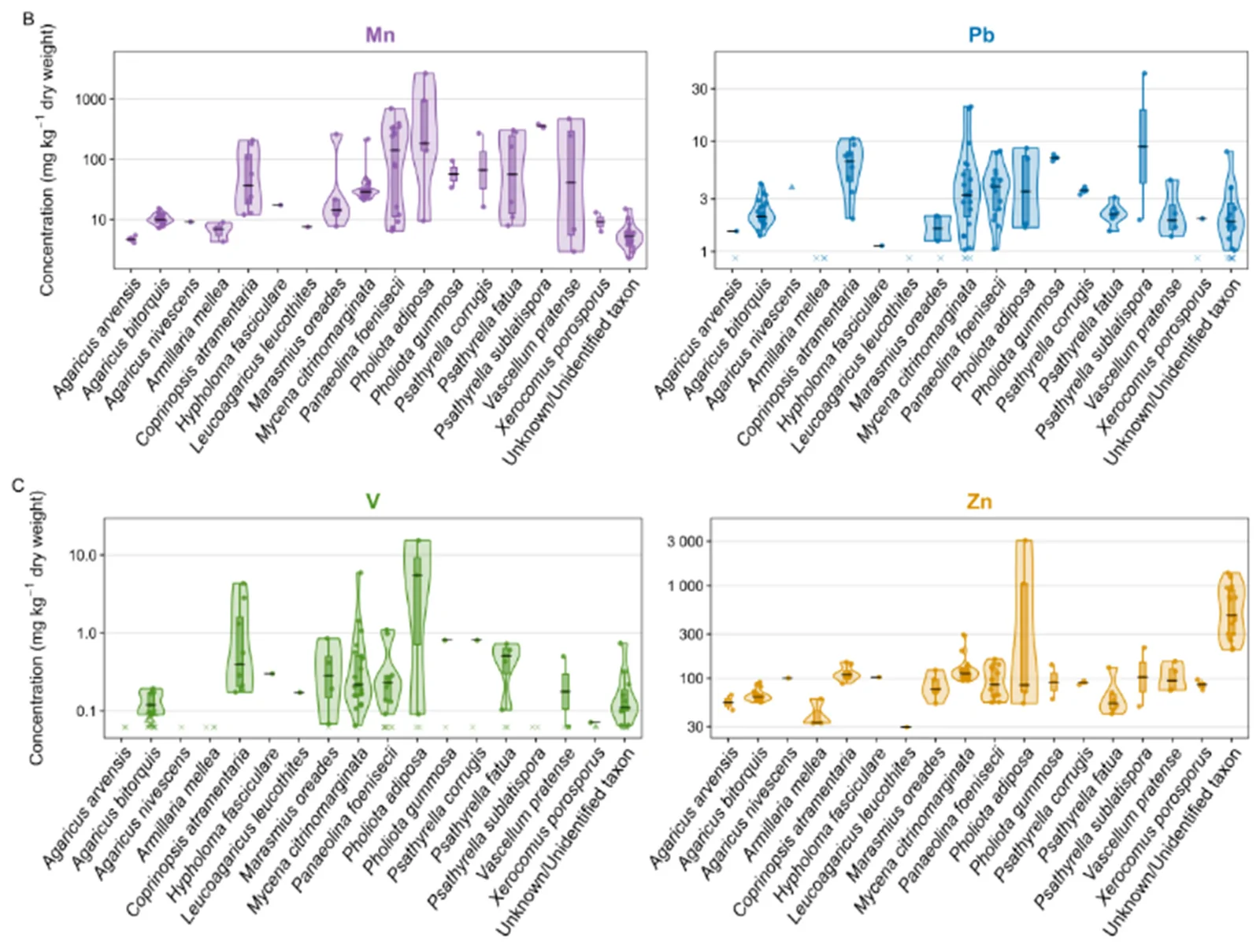
Taxon-level distributions of Cd, Cu, Mn, Pb, V and Zn in the reconstructed fruiting-body-level dataset (*N* = 121): (**A**) Cd and Cu; (**B**) Mn and Pb; and (**C**) V and Zn. Violin envelopes and internal boxplots summarise the distribution, median and interquartile range of detected/measured observations. Superimposed symbols represent individual fruiting-body-level units: circles indicate detected/measured observations, triangles indicate partially censored observations, and crosses indicate fully censored observations. For visualisation only, fully censored observations are plotted at the element-specific limit of detection, whereas partially censored observations are shown at their reconstructed LoD-capped positions; these plotting positions should not be interpreted as measured concentrations. Concentrations are expressed as mg kg^−1^ dry weight and displayed on logarithmic scales.

The taxon-level profiles of Cd, Cu, Mn, Pb, V and Zn are visualised in Figure 2, while selected element distributions across retained taxa are shown in Figure 3.

### 3.3. Cap–Stem Comparison in Agaricus bitorquis

*Agaricus bitorquis* was the only taxon with sufficient paired cap/stem replication for a formal main-text tissue comparison (*n* = 22), as summarised in Table 3. Caps showed higher median concentrations than stems for Cd, Cu, Pb and Zn. Median cap and stem concentrations were 3.878 and 2.068 mg kg^−1^ for Cd, 114.963 and 93.365 mg kg^−1^ for Cu, 2.462 and 1.580 mg kg^−1^ for Pb, and 75.369 and 47.549 mg kg^−1^ for Zn, respectively. The corresponding ratios of cap to stem medians were 1.87 for Cd, 1.23 for Cu, 1.56 for Pb and 1.59 for Zn.

Paired Wilcoxon signed-rank tests confirmed significantly higher concentrations in caps after FDR correction for Cd, Cu, Pb and Zn. The FDR-adjusted *p*-values were <0.001 for Cd, Cu and Zn and 0.004 for Pb. Mn and V did not show statistically significant cap–stem differences after FDR correction.

The sensitivity analysis presented in Table 3 compared hypothetical cap:stem dry-mass contributions of 30:70, 50:50 and 70:30. For elements enriched in caps, the unweighted 50:50 reconstruction would underestimate whole-fruiting-body concentrations if the actual cap dry-mass contribution exceeded 50% and would overestimate them if it were below 50%. These scenarios quantify the direction and approximate magnitude of reconstruction uncertainty but cannot determine the original cap and stem mass proportions.

These findings are consistent with previous studies reporting element- and species-dependent partitioning between mushroom caps and stems [10,14,15,16,17]. Tissue differences should nevertheless not be generalised to other taxa without adequate same-species replication. A broader mixed-taxon tissue-basis overview is provided in Supplementary Table S3, and same-taxon tissue-separated summaries for other taxa are provided in Supplementary Table S4.

### 3.4. Spatial Context: Area Type and Urban Quadrant

Area-type and quadrant summaries were retained as descriptive spatial context only. The dataset comprised 113 urban and eight rural fruiting-body-level units. All eight rural units corresponded to *Coprinopsis atramentaria*, resulting in complete confounding between rural area type and taxonomic composition. Apparent urban–rural differences should therefore not be interpreted as independent area-type effects or as representative of a general rural mushroom baseline.

The urban quadrant summary included 30 NE, 51 NW, 27 SE and five SW fruiting-body-level units. Quadrant composition was also markedly unbalanced. The SE subset was dominated by *Mycena citrinomarginata*, which accounted for 22 of the 27 units, while all five SW units corresponded to *Panaeolina foenisecii*. The NE and NW subsets contained a broader mixture of taxa.

Selected quadrant-level upper-tail records reflected individual observations or taxonomic composition rather than robust spatial gradients. In particular, the high Cd, Mn and Zn records in the NE subset were associated with *Pholiota adiposa*, whereas the high Cu and Ni maxima in the SE subset were associated with *Mycena citrinomarginata*. Consequently, quadrant-level comparisons were not interpreted as independent geographical effects.

Area-type and quadrant concentration summaries are presented in Supplementary Tables S5 and S6 to document the spatial structure and taxonomic imbalance of the dataset. Taxon-level and tissue-level analyses provide the more defensible basis for biological interpretation.

### 3.5. Principal Component Analysis

The primary PCA was restricted to the six sufficiently detected and analytically interpretable elements Cd, Cu, Mn, Pb, V and Zn and included all 121 reconstructed fruiting-body-level units. PC1 explained 32.92% of total variance, PC2 explained 25.31%, and PC3 explained 15.69%, giving a cumulative explained variance of 73.92% across the first three components.

PC1 was associated mainly with Pb, V and Mn, with loadings of 0.588, 0.551 and 0.503, respectively. PC2 was associated mainly with Zn, Cu and Cd, with loadings of 0.566, 0.540 and 0.507, respectively. PC3 was dominated by Cd, with a loading of 0.757, followed by Mn, with a loading of 0.440. Because the signs of PCA loadings are arbitrary, interpretation focused on relative element groupings and separation among components rather than on loading direction alone.

**Figure 4:**
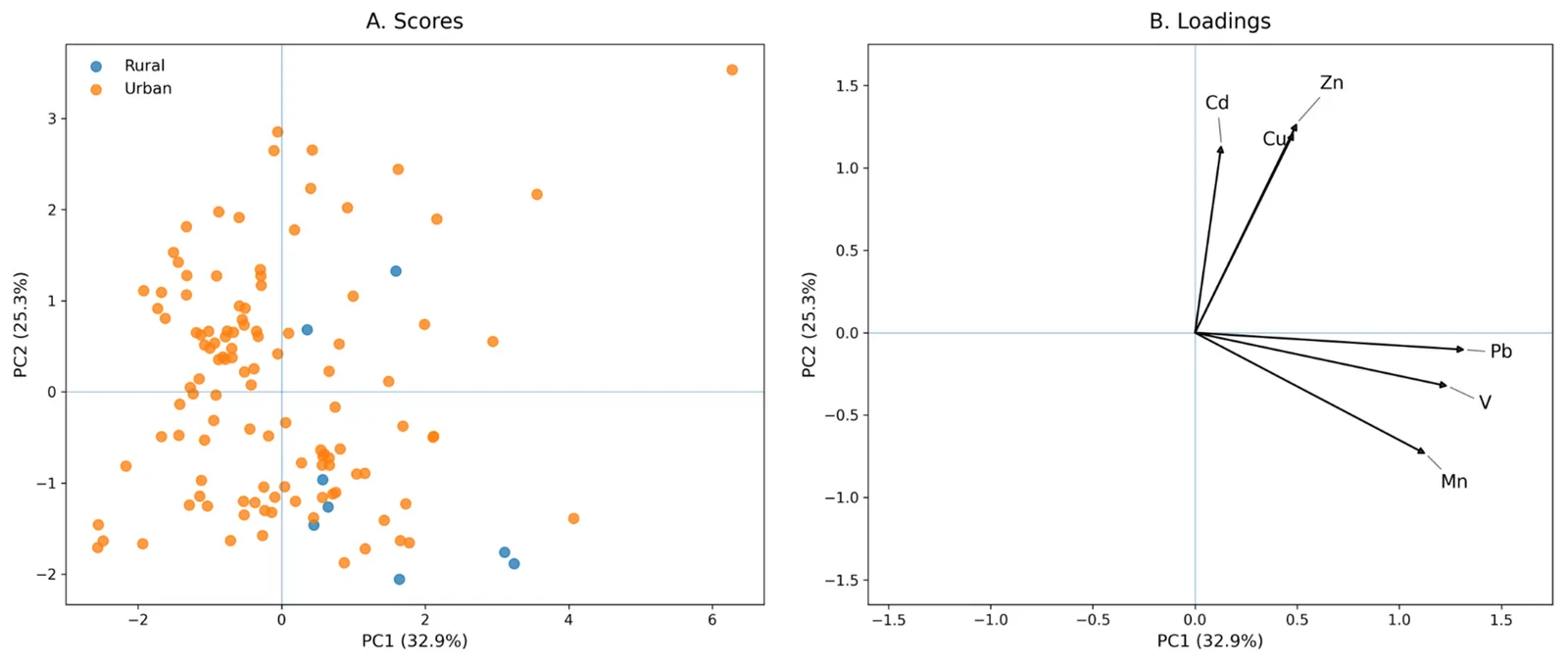
Primary principal component analysis of Cd, Cu, Mn, Pb, V and Zn in the reconstructed fruiting-body-level mushroom dataset (*N* = 121). (**A**) Scores plot, with area type shown for descriptive visualisation only. (**B**) Loadings plot for the six elements. The rural subset was small and taxonomically confounded and should not be interpreted as evidence of an independent urban–rural effect.

The primary ordination therefore distinguished a Pb–V–Mn-associated pattern from a Zn–Cu–Cd-associated pattern, while PC3 captured additional Cd-related variability. The broader ten-element PCA excluding Be was retained only as a supplementary sensitivity analysis because As, Co, Cr and Ni were more strongly affected by censoring and/or analytical uncertainty. The PCA including Be was retained as diagnostic only and was not used to support biological interpretation. The primary scores and loadings are shown in Figure 4, and the complete PCA reporting summary is provided in Supplementary Table S7, Panel A.

Sensitivity analyses supported the stability of the primary PCA under alternative treatments of censored observations. Relative to the primary LoD-capped solution, the LoD/2 and ROS-based reconstructions retained the principal loading structure, with Pb, V and Mn contributing predominantly to PC1, Cd, Cu and Zn to PC2, and Cd and Mn to PC3. As shown in Supplementary Figure S1, the direction and relative grouping of the element loadings were broadly consistent across the three censored-value treatments, although modest differences in loading magnitude were observed. Procrustes correlations with the primary solution were 0.993 for LoD/2 and 0.985 for ROS, and the cumulative variance explained by PC1–PC3 remained similar at 73.51% and 73.84%, respectively, compared with 73.92% in the primary analysis. The same dominant loading structure was retained across all 100 censored-value imputations (Supplementary Table S7, Panel B).

Exclusion of the flagged extreme records produced only minor changes in the global configuration of the PCA scores, with Procrustes correlations ranging from 0.990 to 0.998. Supplementary Figure S2 shows that the overall separation of the Pb–V–Mn and Cd–Cu–Zn loading groups was retained, but that the magnitude and relative ordering of selected PC1 and PC2 loadings changed moderately when the multielement extreme record was excluded, either alone or jointly with the second flagged record. The corresponding loading-congruence coefficients ranged from 0.823 to 0.866 for PC1 and PC2, whereas PC3 was comparatively more stable. Bootstrap analysis further supported Pb, V and Mn as the principal contributors to PC1, Cd, Cu and Zn to PC2, and Cd and Mn to PC3. The global PCA configuration was therefore robust, but detailed interpretation of individual PC1 and PC2 loading magnitudes requires caution (Supplementary Table S7, Panel B).

### 3.6. Contextual Soil Physicochemical Properties and Soil–Mushroom Associations

The contextual topsoil dataset was used only to support interpretation of mushroom multielement patterns at park/area level. Soil physicochemical properties differed between urban and rural composites. Urban soils were less acidic than rural soils, with mean pH values of 6.59 ± 0.82 and 5.83 ± 0.90, respectively. Rural soils showed higher electrical conductivity than urban soils (0.45 ± 0.13 vs. 0.34 ± 0.09 dS m^−1^) and higher clay content (28.56 ± 10.35% vs. 22.23 ± 4.24%). In contrast, urban soils had higher sand content than rural soils (26.40 ± 6.56% vs. 17.31 ± 11.36%). Organic matter and moisture content did not differ significantly between urban and rural composites.

These physicochemical contrasts provide useful environmental context for interpreting mushroom data, but they do not constitute direct predictors of fruiting-body composition. Soil pH, texture, organic matter and moisture may influence metal sorption, solubility, fine-particle retention and bioavailability; however, mushroom fruiting bodies also reflect taxon-specific uptake, mycelial distribution, substrate type, microhabitat and natural fruiting patterns. Therefore, the soil physicochemical dataset was interpreted as contextual information rather than as a mechanistic uptake model.

After harmonisation of the park identifiers, the final soil–mushroom correlation analysis used a fixed set of eight overlapping parks. None of the six primary same-element correlations for Cd, Cu, Mn, Pb, V and Zn remained statistically significant after Benjamini–Hochberg adjustment of the exact permutation *p*-values. The primary analysis therefore provided no statistically supported evidence that higher park-level soil concentrations of these elements were associated with corresponding increases or decreases in the park-level mushroom summaries.

The broader exploratory panel included additional same-element, cross-element and soil-physicochemical associations. None remained statistically significant after either the applicable within-family adjustment or the global false-discovery-rate correction. Nominally large coefficients were interpreted cautiously because the analysis included only eight parks and because both the number and taxonomic composition of the mushroom units differed markedly among parks. The complete primary and exploratory correlation results are reported in Supplementary Table S8.

Leave-one-park-out analysis comprised eight repetitions, each based on the remaining seven parks. The magnitude and, for some associations, the direction of the coefficients varied according to the omitted park, and no consistently supported association emerged across the repetitions. These findings indicate that the apparent park-level relationships were sensitive to individual parks and to the taxonomically unbalanced sampling structure. The complete leave-one-out results are provided in Supplementary Table S9.

Overall, the soil dataset was retained as environmental context rather than as evidence of direct soil-to-mushroom transfer. This interpretation reflects the mismatch in spatial scale between homogenised park-level soil composites and opportunistically collected mushrooms, together with the influence of fungal taxonomy, substrate, mycelial distribution, microhabitat and tissue-specific element handling. This cautious interpretation is consistent with previous evidence that mineral soils and substrates may be only moderate-to-weak predictors of element concentrations in sporocarps [34].

### 3.7. Adult Dietary Exposure Screening for Edible and Edible-with-Caution Taxa

Adult dietary screening based on the central concentration estimates is summarised in Table 4. The primary screening block comprised edible and edible-with-caution taxa represented by reconstructed fruiting-body-level units. No quantitative result was reported for taxon–element combinations that did not meet the censored-data estimation criteria. Single-tissue records for which a whole-fruiting-body-level unit could not be reconstructed, including *Macrolepiota procera*, were excluded from the quantitative screening. The corresponding hypothetical-ingestion screening for non-edible, toxic, uncertain-edibility and unidentified taxa is summarised separately in Table 5.

**Figure 5:**
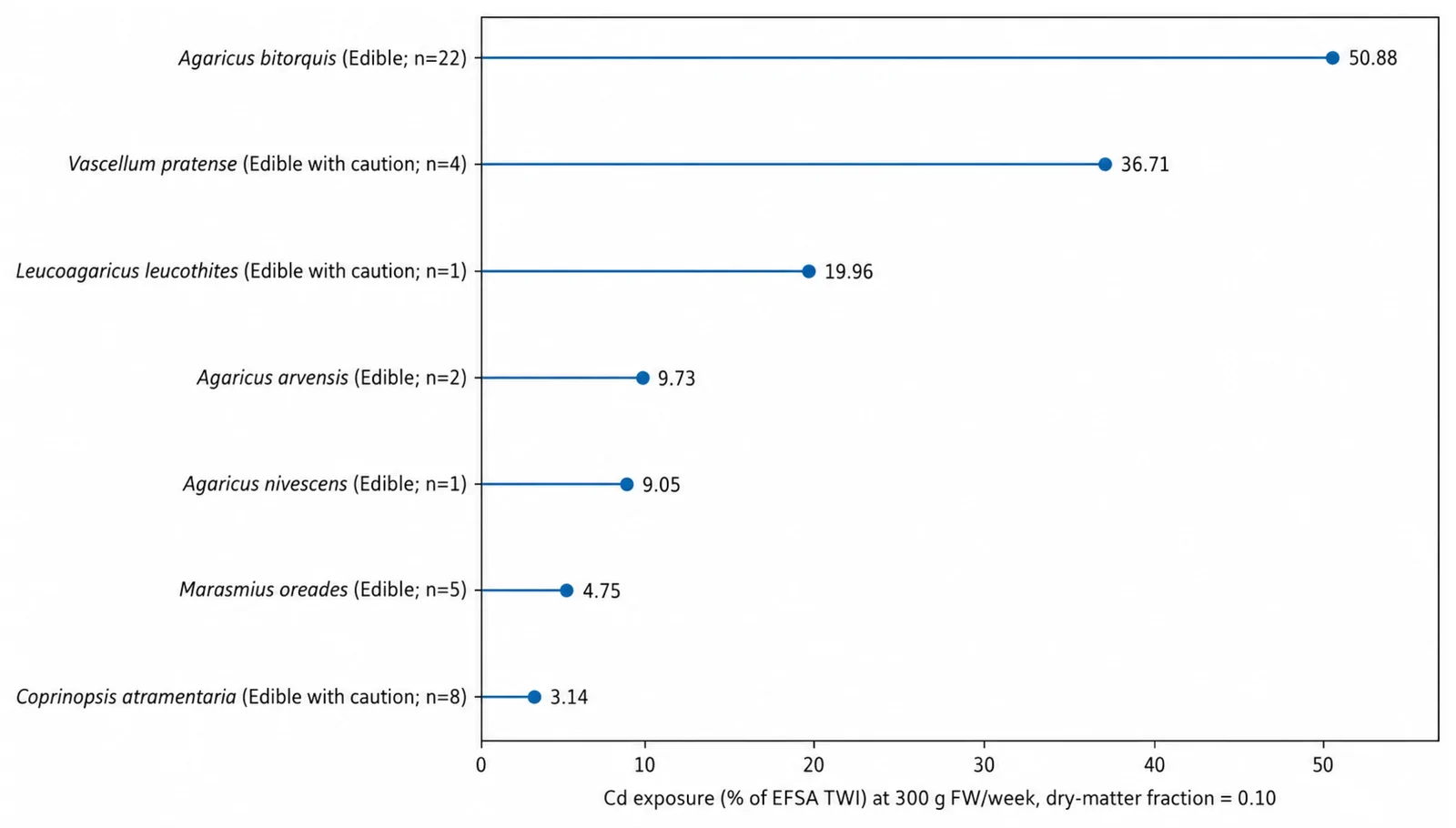
Cadmium exposure expressed as a percentage of the EFSA tolerable weekly intake under the 300 g fresh-weight week^−1^ scenario and an assumed dry-matter fraction of 0.10 for edible and edible-with-caution taxa. Bars represent central concentration estimates selected under the censored-data framework. Results assume 100% bioaccessibility and represent screening estimates rather than food-safety determinations.

Under the 300 g fresh-weight week^−1^ scenario and a dry-matter fraction of 0.10, Cd was the main threshold-based screening driver for several taxa. Cd exposure corresponded to 50.88% of the EFSA TWI for *Agaricus bitorquis*, 36.71% for *Vascellum pratense*, 19.96% for *Leucoagaricus leucothites*, 9.73% for *Agaricus arvensis* and 9.05% for *Agaricus nivescens*. For *Marasmius oreades*, Cu represented the largest percentage-based result, at 5.49% of the selected safe level, followed by Cd at 4.75% of the TWI. For *Coprinopsis atramentaria*, Cu represented 7.07% of the selected safe level, Ni 5.12% of the TDI and Cd 3.14% of the TWI. These central Cd screening estimates are visualised in Figure 5.

The harmonised pooled edible-relevant reference population (*N* = 43) identified Cd as the principal threshold-based driver under the 300 g fresh-weight week^−1^ scenario, corresponding to 47.33% of the TWI; the corresponding Cu estimate was 8.70% of the EFSA safe level, and the Pb MOE was 3.88. Taxon-specific screening did not, however, reproduce this pooled profile uniformly. Cd estimates among taxa with reportable central values ranged from 3.14% of the TWI for *Coprinopsis atramentaria* to 50.88% for *Agaricus bitorquis*, while reportable Pb MOEs ranged from 1.23 for *C. atramentaria* to 6.53 for *Marasmius oreades*. Moreover, the principal threshold-based driver changed from Cd in the pooled reference population to Cu in *C. atramentaria* and *M. oreades*. These differences show that the pooled edible-relevant summary did not adequately represent the magnitude or ranking of the screening outputs for all individual taxa and therefore support the taxon-specific component of the working hypothesis.

Among the MOE-based outputs, *C. atramentaria* had an As MOE of 1.25 and a Pb MOE of 1.23 under the 300 g scenario. The corresponding Pb MOEs were 3.92 for *A. bitorquis*, 4.21 for *V. pratense* and 6.53 for *M. oreades*. No As MOE was calculated for *A. bitorquis* because As was not detected in this taxon and no defensible central concentration estimate was available.

Among the individual edible-relevant taxa, a high-end scenario could be calculated only for *A. bitorquis*, because it was the only taxon meeting all prespecified P95 eligibility criteria. At 300 g fresh weight week^−1^, the P95-based Cd estimate corresponded to 72.52% of the TWI, compared with 50.88% using the central estimate, while the Pb MOE decreased from 3.92 to 2.41. The complete central and high-end comparisons are reported in Supplementary Table S10.

Sensitivity calculations using dry-matter fractions of 0.08, 0.10 and 0.12 are presented in Supplementary Table S11. These scenarios demonstrate the dependence of the screening results on the assumed moisture conversion but do not resolve the absence of taxon-specific moisture measurements. All results assume 100% bioaccessibility and should be interpreted as screening estimates rather than absorbed-dose estimates or food-safety determinations.

### 3.8. Hypothetical Ingestion Screening for Non-Edible, Uncertain-Edibility and Unidentified Taxa

Taxa classified as non-edible, toxic, of uncertain edibility or unidentified were retained separately as hypothetical-ingestion scenarios, as summarised in Table 5. These calculations indicate potential metal(loid) drivers in the event of accidental ingestion, misidentification or deliberate consumption, but they do not constitute dietary-safety assessments. They do not account for intrinsic fungal toxins, psychoactive compounds, gastrointestinal toxicity, preparation-related hazards or other non-metal risks.

Under the central-estimate 300 g fresh-weight week^−1^ scenario, *Panaeolina foenisecii* had a Cd estimate corresponding to 43.99% of the TWI, Mn corresponding to 8.72% of the selected safe level and a Pb MOE of 2.10. No As result was reported for this taxon because the criteria for central estimation were not met. *Pholiota adiposa* had Cd and Mn estimates corresponding to 43.19% and 9.85% of their respective reference values. *Psathyrella sublatispora* had a Cd estimate of 58.46% of the TWI and a Pb MOE of 0.38. The Unknown/Unidentified taxon group had an As MOE of 0.50, Cd corresponding to 41.51% of the TWI and Zn corresponding to 8.16% of the UL. *Xerocomus porosporus* had a Cd estimate corresponding to 92.80% of the TWI.

High-end estimates were calculated only for hypothetical-ingestion groups meeting all prespecified P95 eligibility criteria. Among these groups, only *Mycena citrinomarginata* (*N* = 22) was eligible. Under the 300 g fresh-weight week^−1^ scenario, its P95-based Pb MOE was 0.42, while the corresponding high-end Cu and Ni estimates represented 74.47% of the EFSA safe level and 35.39% of the TDI, respectively. No P95-based screening result was reported for *Panaeolina foenisecii* or the Unknown/Unidentified taxon group because their sample sizes did not meet the predefined eligibility threshold. These results illustrate upper-tail sensitivity within a hypothetical ingestion scenario but should not be interpreted as evidence of the safety, toxicity or likely consumption of this taxon. The complete central and high-end outputs are reported in Supplementary Table S10.

The separation of these taxa from the edible-relevant block is essential. In particular, *P. foenisecii* was classified as non-edible/uncertain-edibility and retained for hypothetical ingestion only. Reports of limited clinical effects following small accidental ingestions do not constitute evidence of edibility or justify its inclusion in a dietary-screening table [48,49].

## 4. Discussion

### 4.1. Wild Mushrooms as Taxon-Specific Multielement Food Matrices in Comparison with Previous Studies

The present study confirms that wild mushrooms from Leicester/Leicestershire are chemically heterogeneous food matrices. Cu, Mn and Zn were consistently detected, Cd and Pb were broadly detected, and As, Co and Be showed substantial censoring. This profile is consistent with the wider literature showing that wild mushrooms may contain both nutritionally relevant elements and potentially toxic metal(loid)s, but that their concentrations are strongly taxon-, tissue- and environment-dependent [5,6,7,8,9,50].

The most relevant UK comparator remains the survey by Weeks et al. [11]. Using the same approximate 10% dry-matter conversion applied in the present screening, our fresh-weight median estimates for Cd and Pb were close to the UK medians reported by Weeks et al. [11], supporting the interpretation that the central tendency of these two major toxicological elements is broadly compatible with previous UK wild-fungi data. Approximate fresh-weight Cu and Zn medians in the present dataset were also broadly comparable with the UK medians reported [11]. However, the present dataset showed pronounced upper-tail variability for Cu, Zn, Mn, Ni and Cd, indicating that screening-level interpretation should not rely only on pooled medians. Taxon-specific summaries and, where the prespecified eligibility criteria were met, high-end estimates were informative because relatively small numbers of fruiting bodies could influence the upper tail of the distributions.

Comparison with wild edible mushrooms from Greece further supports this interpretation. Ouzouni et al. [4] reported dry-weight ranges of 0.12–5.34 mg kg^−1^ for Cr, 7.19–62.63 mg kg^−1^ for Mn, 0.05–7.22 mg kg^−1^ for Co, 0.76–9.93 mg kg^−1^ for Ni, 7.38–75.06 mg kg^−1^ for Cu, 34.43–98.99 mg kg^−1^ for Zn, not detected–1.16 mg kg^−1^ for Pb and 0.07–1.80 mg kg^−1^ for Cd in ten wild edible mushroom species. The present overall medians for Cr, Mn, Ni, Cu and Zn fall within or near these reported ranges, whereas Pb and Cd were higher than the upper end of that Greek edible-mushroom dataset. This difference should not be interpreted as a direct geographical contrast because the present study included urban/peri-urban green spaces, non-edible and unknown taxa, reconstructed fruiting-body units and pronounced upper-tail values. Nevertheless, it reinforces the need for site- and taxon-aware interpretation when wild mushrooms are considered as food matrices.

The Polish edible-fungi study by Gałgowska and Pietrzak-Fiećko [22] reported mean Cd concentrations of 0.370–2.151 mg kg^−1^ dry weight and Pb concentrations of 0.243–0.424 mg kg^−1^ dry weight in selected edible fungi. The present overall Cd median of 1.856 mg kg^−1^ dry weight is therefore within a comparable range, whereas the overall Pb median of 2.171 mg kg^−1^ dry weight is higher. This contrast may reflect differences in species composition, environmental setting, urban influence, analytical-unit definition and the inclusion of taxa not intended for edible screening in the present overall mushroom dataset.

The Turkish studies by Demirbaş [7] and Doğan et al. [51] also demonstrate the broad variability of metal concentrations in wild mushrooms. Demirbaş [7] reported maximum dry-weight concentrations of 4.91 mg kg^−1^ for Pb, 3.48 mg kg^−1^ for Cd, 92.5 mg kg^−1^ for Cu, 44.4 mg kg^−1^ for Mn, 176 mg kg^−1^ for Zn, 0.72 mg kg^−1^ for Co, 1.76 mg kg^−1^ for As, 145 mg kg^−1^ for Ni and 1.68 mg kg^−1^ for Cr among 18 mushroom species from the East Black Sea region. Doğan et al. [51] reported maximum concentrations of 39 mg kg^−1^ for Pb, 3.72 mg kg^−1^ for Cd, 467 mg kg^−1^ for Mn, 326 mg kg^−1^ for Cu, 69.4 mg kg^−1^ for Ni and 84.5 mg kg^−1^ for Cr. In the present dataset, the upper-tail values for Cd, Cu, Mn, Ni, Pb and Zn were similar to or higher than those reported in these earlier surveys, while Cr remained below the maximum reported by Doğan et al. [51]. These comparisons indicate that the Leicester/Leicestershire dataset is not unusual in showing strong interspecific and upper-tail variability, but it does contain selected high-concentration records that are important for screening interpretation.

The Italian survey by Cocchi et al. [8], which included 1194 samples from 60 edible mushroom species, is particularly useful because it demonstrates that extreme accumulation can be highly taxon-specific. That study showed generally modest As in most samples but very high concentrations in selected taxa, and it highlighted Cd accumulation in some *Agaricus* and *Boletus* groups. The present finding of elevated Cd in *Agaricus bitorquis* and selected upper-tail records in other taxa is consistent with the broader conclusion that edible or potentially edible mushrooms cannot be treated as a chemically uniform food category.

Cultivated mushrooms provide a useful but limited comparator. Rashid et al. [27] reported mean concentrations of 0.51 mg kg^−1^ for As, 0.38 mg kg^−1^ for Cd, 0.28 mg kg^−1^ for Cr, 13.7 mg kg^−1^ for Cu, 0.31 mg kg^−1^ for Pb, 11.7 mg kg^−1^ for Mn, 0.28 mg kg^−1^ for Ni and 53.5 mg kg^−1^ for Zn in mushrooms from Bangladesh. The present wild-mushroom medians were generally higher for Cd, Cu, Pb, Ni and Zn. However, this comparison should be interpreted cautiously because cultivated mushrooms differ from wild fruiting bodies in species composition, substrate control, environmental exposure, management and post-harvest handling.

Overall, comparison with previous studies supports three main conclusions. First, the central tendency of Cd, Cu, Pb and Zn in the present study was broadly consistent with published wild-mushroom datasets, particularly the UK survey by Weeks et al. [11]. Second, the pronounced upper-tail variability for selected elements and taxa supports reporting detection frequencies, taxon-level distributions and high-end estimates only where the prespecified P95 eligibility criteria were met, rather than extrapolating unstable percentiles from small or highly censored groups. Third, comparison with the pooled edible-relevant reference population showed that taxon-specific screening could alter both the magnitude and the ranking of the principal exposure drivers. Although Cd was the main threshold-based driver in the pooled population, Cu became the principal percentage-based driver for *Coprinopsis atramentaria* and *Marasmius oreades*, while Cd estimates also varied substantially among individual edible-relevant taxa. These findings support the taxon-specific component of the working hypothesis, but they do not imply that the available taxa constitute a balanced representation of wild mushrooms generally.

### 4.2. Fruiting-Body-Level Reconstruction Improves Dietary Interpretability

A major methodological strength of this study is the reconstruction of fruiting-body-level units for the main descriptive and dietary-screening analyses. In field mushroom datasets, cap/stem separation is analytically valuable, but it creates a risk of pseudo-replication if tissue records are treated as independent food units. The present approach avoids this problem by averaging paired cap/stem records without tissue-mass weighting.

This strategy is especially relevant for dietary screening. A consumer normally eats a fruiting body or a portion of a fruiting body, not a statistical mixture of independently counted caps and stems. Although mass-weighted reconstruction would be preferable, the unweighted paired-tissue average provides a transparent compromise that avoids double counting and preserves information from paired tissues.

The *Agaricus bitorquis* tissue comparison showed higher cap medians for Cd, Cu, Pb and Zn. Similar cap-enrichment patterns have been reported for some metals in other wild mushroom studies [14,15,16]. However, tissue partitioning is not universal and should not be generalised across taxa. The present study therefore appropriately restricts main-text tissue interpretation to *A. bitorquis*. The cap:stem sensitivity analysis further demonstrated that the direction of reconstruction bias depends on the true dry-mass contribution of each tissue. For cap-enriched elements, the unweighted 50:50 reconstruction would underestimate whole-fruiting-body concentrations when the cap contributed more than 50% of the dry mass and would overestimate them when the cap contribution was lower. The 30:70 and 70:30 scenarios quantify this uncertainty but cannot retrospectively determine the actual tissue-mass proportions.

### 4.3. PCA Supports a Multielement Fingerprint Interpretation

The primary PCA, restricted to Cd, Cu, Mn, Pb, V and Zn, provided a compact multielement summary of the reconstructed fruiting-body-level dataset. The first three components explained 73.92% of the total variance, with PC1 accounting for 32.92%, PC2 for 25.31% and PC3 for 15.69%. This cumulative variance indicates that a substantial proportion of the multielement structure was captured by the first three axes, despite the heterogeneous taxonomic composition of the dataset. Restricting the primary ordination to these six elements reduced the potential influence of strongly censored or analytically uncertain variables.

PC1 was mainly associated with Pb, V and Mn, with loadings of 0.588, 0.551 and 0.503, respectively. This axis therefore reflected a combined Pb–V–Mn-associated pattern rather than a single-element gradient. Although these elements differed in their toxicological and nutritional interpretation, their covariance contributed to the overall differentiation of fruiting-body-level units. This supports the value of PCA as a multielement fingerprinting tool complementary to the univariate concentration summaries and dietary-screening outputs.

PC2 was characterised mainly by Zn, Cu and Cd, with loadings of 0.566, 0.540 and 0.507, respectively. This pattern indicates that the consistently detected nutritionally relevant elements Cu and Zn covaried with Cd within the second component, despite their different biological and toxicological significance. The distinction between the Pb–V–Mn-associated PC1 pattern and the Zn–Cu–Cd-associated PC2 pattern suggests that fungal elemental profiles were shaped by multiple taxon-, tissue- and environment-related processes rather than by a single nutritional or contamination axis.

PC3 was dominated by Cd, with a loading of 0.757, followed by Mn with a loading of 0.440. The strong Cd contribution to PC3 is relevant because Cd was also the main threshold-based dietary-screening driver in several edible or edible-with-caution taxa. Its additional contribution to PC3 indicates that Cd-related variability was not fully represented by the dominant PC1 or PC2 patterns. This reinforces the need to interpret Cd both as an exposure-relevant element and as part of a broader multielement fungal fingerprint.

Overall, the primary PCA supports interpretation of wild mushroom chemistry as a multielement fingerprint rather than as a set of isolated single-element distributions or a simple soil-contamination gradient. The PCA was performed on the same reconstructed fruiting-body-level dataset used for the main descriptive and dietary-screening analyses, thereby avoiding double counting of paired cap/stem records. The resulting ordination should nevertheless be interpreted as an exploratory summary of covariance at fruiting-body level and not as evidence of mechanistic soil-to-mushroom transfer or common contamination sources.

The additional sensitivity analyses strengthen, but do not make absolute, this interpretation. The high Procrustes correlations, indicating close agreement between the aligned PCA score configurations, obtained for the LoD/2 and ROS-based reconstructions, together with retention of the dominant loading structure across all 100 censored-value imputations, indicate that the principal Pb–V–Mn and Cd–Cu–Zn groupings were not primarily artefacts of the selected censored-value treatment. Similarly, exclusion of the flagged extreme records produced only minor changes in the global score configuration. However, the moderate PC1 and PC2 loading-congruence coefficients observed when the multielement-extreme record was removed indicate that the precise magnitude and ordering of individual loadings were partly influenced by isolated upper-tail observations. The PCA therefore provides reasonably stable evidence of broad multielement covariance patterns, while the detailed hierarchy of individual PC1 and PC2 contributions should be interpreted cautiously. This multivariate evidence supports the fingerprinting component of the working hypothesis, but the PCA should not be interpreted as a predictive model of dietary exposure or environmental source.

The broader ten-element PCA excluding Be was retained as a supplementary sensitivity analysis and explained 67.04% of the variance across PC1–PC3. Its first component was associated mainly with Co, Cr, Ni and V, while PC2 was associated principally with As and Zn and PC3 with Cd. However, As, Co, Cr and Ni were more strongly affected by censoring and/or analytical uncertainty, and these loadings were therefore not used as primary biological evidence. The full PCA including Be explained 61.48% of the variance across PC1–PC3 and was retained for diagnostic purposes only, because Be was 99.2% censored but nevertheless contributed strongly to PC3. These sensitivity analyses support the decision to use the six-element PCA as the primary ordination. Because PCA loading signs are orientation-dependent, interpretation should focus on the magnitude and grouping of element loadings rather than on their signs alone.

### 4.4. Soil Context Does Not Equal Individual Uptake

This sampling design means that the study is environmentally contextualised but not microsite-paired. The contextual topsoil dataset characterises the broader geochemical setting of the parks and green spaces where mushrooms were collected, based on homogenised composite samples derived from 850 field increments. However, because soil was not sampled directly from the immediate surface/substrate zone of each fruiting body, taxon-level differences in mushroom chemistry are interpreted as field-observed multielement profiles rather than intrinsic accumulation capacities or mechanistic soil-to-fruiting-body transfer relationships. Therefore, even when soil and mushroom data are matched at park or area level, they do not represent paired measurements of the precise substrate explored by each mycelial network.

The companion topsoil dataset showed clear but element-specific environmental structure [13]. Urban Leicester topsoils had significantly higher median Cr, Cu and Zn than rural Leicestershire topsoils, whereas rural topsoils had significantly higher As and V. Specifically, urban medians were 82.37 mg kg^−1^ for Cr, 43.79 mg kg^−1^ for Cu and 125.9 mg kg^−1^ for Zn, compared with 30.64, 32.68 and 102.4 mg kg^−1^ in rural topsoils, respectively. Conversely, rural medians were higher for As and V, with 18.27 and 61.40 mg kg^−1^ compared with 14.38 and 46.28 mg kg^−1^ in urban topsoils. These patterns indicate that the soil environment of the mushroom sampling framework included both urban enrichment-sensitive elements and rural matrix-related elements. However, they do not imply that mushroom concentrations should mirror the same urban–rural structure.

The contextual soil dataset showed area-level differences in pH, electrical conductivity and texture between urban and rural composites. These contrasts may influence metal mobility, but they do not represent the microsite conditions surrounding individual fruiting bodies.

The harmonised eight-park soil–mushroom analysis reinforced this cautious interpretation. None of the six primary same-element correlations for Cd, Cu, Mn, Pb, V and Zn remained statistically significant after Benjamini–Hochberg adjustment of the exact permutation *p*-values. Likewise, none of the broader same-element, cross-element or soil-physicochemical associations remained significant after either the applicable within-family adjustment or the global false-discovery-rate correction. The analysis therefore provided no statistically supported evidence that park-level soil concentrations or physicochemical properties were consistently associated with the corresponding park-level mushroom summaries.

The leave-one-park-out analysis further showed that correlation magnitudes, and in some cases their direction, varied according to the omitted park. This instability was consistent with the small effective sample size and the taxonomically unbalanced park-level summaries, because the number and composition of the mushroom taxa differed markedly among parks. Nominally large coefficients were therefore treated only as hypothesis-generating environmental context and not as evidence of prediction, individual uptake or mechanistic soil-to-mushroom transfer. Accordingly, the park-level analyses were not used as a formal test of the working hypothesis.

Overall, these results show why soil context should not be conflated with individual uptake. Total or pseudo-total soil metal(loid) concentrations do not capture metal speciation, bioaccessibility, mycelial depth, substrate heterogeneity, microsite pH, fungal developmental stage, tissue partitioning, atmospheric deposition or taxon-specific uptake and sequestration mechanisms. This cautious interpretation is consistent with Hanley et al. [34], who reported that substrates and mineral soils were only moderate-to-weak predictors of element concentrations in foraged mushrooms. It is also consistent with the broader fungal literature showing that taxon, tissue, substrate biology and local microhabitat may be as important as total soil chemistry.

For the present mushroom-focused manuscript, the key message is therefore not that soil is irrelevant, but that soil data must be used at the correct interpretative scale. The composite topsoil dataset provides valuable environmental context for Leicester/Leicestershire green spaces, including urban enrichment-sensitive and rural matrix-related patterns. However, mushroom food chemistry was not reducible to soil concentration or soil physicochemical properties. The most defensible interpretation is that soil chemistry helps frame environmental conditions, whereas mushroom multielement profiles reflect the combined influence of taxonomy, tissue, substrate, microsite conditions and element-specific biological handling.

### 4.5. Dietary Screening: Nutritionally Relevant Elements and Cd as the Main Threshold-Based Driver

The adult dietary screening should be interpreted within the dual nutritional–toxicological nature of wild mushrooms. Cu, Mn and Zn are essential elements and were consistently detected in the present dataset. Their presence contributes to the mineral fingerprint of the mushrooms, but nutritional interpretation cannot be based on concentration alone because dietary benefit depends on portion size, bioavailability, total diet, physiological requirements and upper-intake considerations [4,5,44,45,47]. Therefore, the present study does not attempt to classify the sampled mushrooms as nutritionally beneficial or nutritionally superior foods. Instead, Cu, Mn and Zn are interpreted as nutritionally relevant elements whose concentrations also require screening against upper reference values when consumption is repeated or when concentrations are high.

Cd was the principal threshold-based screening driver for several edible and edible-with-caution taxa. This result is consistent with the wider European literature identifying Cd as one of the main toxicological priorities in wild edible mushrooms [8,15,16,22,23,24]. Under the central-estimate 300 g fresh-weight week^−1^ scenario, Cd exposure corresponded to 50.88% of the EFSA TWI for *Agaricus bitorquis*, 36.71% for *Vascellum pratense*, 19.96% for *Leucoagaricus leucothites*, 9.73% for *Agaricus arvensis* and 9.05% for *Agaricus nivescens*. For *A. bitorquis*, which was the only individual edible-relevant taxon meeting all prespecified P95 eligibility criteria, the P95-based high-end scenario increased the Cd estimate to 72.52% of the TWI. These results do not imply that the edible-relevant taxa are safe or unsafe under occasional consumption, because the calculations are screening estimates based on assumed dry-matter content, 100% bioaccessibility and simplified adult intake scenarios. They do, however, indicate that Cd is the element most likely to drive prioritisation when high seasonal consumption, repeated foraging or Cd-accumulating taxa are considered.

Although the present manuscript is based primarily on dietary screening rather than regulatory compliance assessment, Cd can also be contextualised against the European maximum level for wild fungi. Commission Regulation (EU) 2023/915 [52] establishes a maximum level of 0.50 mg kg^−1^ fresh weight for Cd in wild fungi, applied after washing and separating the edible portion. Using the same approximate 10% dry-matter conversion adopted in the present screening calculations, this corresponds to an indicative dry-weight equivalent of approximately 5.0 mg kg^−1^ dw. This comparison should be interpreted only as a screening approximation because sample-specific moisture contents were not measured. Under this assumption, both the central and P95 concentration estimates for *A. bitorquis*, 2.968 and approximately 4.230 mg kg^−1^ dw, respectively, remained below this approximate dry-weight equivalent. However, individual high-Cd tissue-specific records reported in the companion Cd–Cu–Zn study indicate that selected cap samples may exceed this approximate benchmark. Therefore, the regulatory comparison supports the same conclusion as the dietary screening: Cd remains the priority element for cautious interpretation of high-Cd taxa, cap tissues and mushrooms collected from urban verge or roadside environments [15,52].

The comparison with Gałgowska and Pietrzak-Fiećko [22] is informative because their highest Cd exposure estimate reached 30.87% of the TWI for *Boletus edulis*. The central and P95-based *A. bitorquis* estimates in the present 300 g week^−1^ scenario were higher, although direct comparison is limited by differences in species, environmental setting, intake assumptions and analytical design. The Slovak study by Árvay et al. [23] also identified Cd as a relevant contributor to weekly intake in selected wild edible mushrooms from a mining-influenced region. Together, these studies support the conclusion that Cd exposure from wild mushrooms can be non-negligible in specific taxa or environments and should be assessed taxon-specifically.

### 4.6. Arsenic and Lead MOEs: Conservative Screening Rather than Definitive Risk Characterisation

As and Pb were evaluated using MOE-based screening rather than threshold-based percentage metrics. This approach is appropriate because both elements require toxicological frameworks that differ from those applied to nutritionally relevant elements or other substances with intake-based reference values. For Pb, the MOE approach reflects the absence of a clear safe exposure threshold for key endpoints, particularly developmental neurotoxicity, and supports interpretation as a screening-priority metric rather than a binary safe/unsafe classification [21]. Under the central-estimate 300 g fresh-weight week^−1^ scenario, the pooled edible-relevant reference population had a Pb MOE of 3.88. The corresponding taxon-specific MOEs ranged from 1.23 for *Coprinopsis atramentaria* to 6.53 for *Marasmius oreades*, with intermediate values of 3.92 for *Agaricus bitorquis* and 4.21 for *Vascellum pratense*. This variation indicates that the pooled estimate did not represent all individual edible-relevant taxa equally and supports the taxon-specific component of the working hypothesis. The approximate fresh-weight central Pb concentration for the overall dataset was close to the UK wild-fungi median reported by Weeks et al. [11]; nevertheless, the taxon-specific MOEs indicate that Pb should remain part of wild-mushroom monitoring even when overall central concentrations appear broadly comparable with previous UK data.

For As, the principal limitation is speciation. Total As was treated as inorganic As for conservative screening, but this is not equivalent to measured inorganic As. Within the edible-relevant block, *C. atramentaria* had a defensible central concentration estimate and an As MOE of 1.25 under the 300 g fresh-weight week^−1^ scenario. No defensible As MOE could be estimated for *A. bitorquis* because As was not detected in this taxon. Mushroom studies have shown that As species can include inorganic As, arsenobetaine, arsenosugars, dimethylarsinic acid and monomethylarsonic acid, with the relative contribution of each species varying among taxa and environmental settings [25,26,27,28,29,30]. In cultivated and market mushrooms, total As and As localisation can also vary between species and fruiting-body tissues [28,31]. Therefore, the As MOEs retained in the present study should be interpreted as conservative triggers for further As speciation work rather than as definitive evidence of inorganic-As risk.

### 4.7. Chromium, Nickel and Other Screening Elements

Cr screening also requires explicit chemical-form caution. Total Cr was excluded from the primary quantitative dietary screening because Cr speciation was not measured and total Cr cannot distinguish Cr(III), Cr(VI) or organically complexed Cr species [32,33]. A 16.7% scenario, mathematically equivalent to total Cr/6, was retained only within the supplementary sensitivity analysis alongside assumed Cr(VI) fractions of 1%, 10% and 100%. These scenarios are not measured or literature-derived fractions and should not be interpreted as evidence that Cr(VI) was present. The corresponding sensitivity results are reported in Supplementary Table S12.

Ni was moderately censored in the overall fruiting-body-level dataset but showed high upper-tail values in selected records. Nickel is relevant because dietary exposure may be of concern for sensitive individuals, and EFSA has updated its risk assessment for nickel in food and drinking water [43]. However, because Ni detection and concentration patterns were strongly taxon-dependent in the present dataset, Ni should be interpreted as a taxon-specific screening element rather than as a uniform dietary driver across all wild mushrooms.

Be and Co were highly censored and therefore unsuitable for strong dietary interpretation in this dataset. Their inclusion in the analytical panel remains useful for multielement screening, but the high censoring for Be and Co means that absence of reported medians should not be interpreted as nutritional or toxicological absence. Instead, it reflects insufficient quantifiable information for robust distributional summaries. This reinforces the value of explicit censoring-aware reporting in wild-mushroom exposure-screening studies.

### 4.8. Non-Edible, Uncertain-Edibility and Unknown Taxa

The separate hypothetical-ingestion block is essential. *Panaeolina foenisecii* should not be combined with edible taxa in a dietary-screening table. Although small accidental ingestions have not consistently produced clinically relevant effects, this does not establish edibility [48,49]. Accordingly, the taxon was classified as non-edible/uncertain-edibility and retained only for hypothetical-ingestion calculations.

For all non-edible, uncertain-edibility and unknown taxa, metal-based calculations do not capture intrinsic mushroom toxicity, psychoactive compounds, gastrointestinal toxins, misidentification risk or preparation-related risks. The purpose of the block is only to quantify metal-based screening values under hypothetical ingestion. It is not a food-safety endorsement.

### 4.9. Strengths and Limitations

The main strengths of this study include the transparent distinction between the present analysis and the companion mushroom and topsoil studies; reconstruction of the main dataset at fruiting-body level to avoid cap/stem pseudo-replication; explicit reporting of detection frequency and censoring; prespecified eligibility criteria for ROS-derived central estimates and P95 values; taxon- and tissue-aware dietary screening; and the same-species cap/stem comparison for *Agaricus bitorquis*. The primary PCA was restricted to sufficiently detected elements and was evaluated using alternative censored-value treatments, repeated imputations, exclusion of flagged extreme records and bootstrap assessment of the loading structure. Environmental context was also evaluated using a fixed eight-park overlap, exact permutation tests, false-discovery-rate correction and leave-one-park-out sensitivity analysis. The separation of edible-relevant taxa from non-edible, toxic, uncertain-edibility and unidentified taxa further reduced the risk of conflating dietary screening with hypothetical ingestion.

Several limitations constrain the generalisability of the findings. Opportunistic collection resulted in uneven replication among taxa, parks and edibility categories, and the rural mushroom component was represented primarily by Bradgate Park. The pooled edible-relevant population comprised 43 reconstructed fruiting-body-level units but was introduced only as an analytical comparator for evaluation of the working hypothesis; it was not taxonomically or spatially balanced and should not be interpreted as a representative population estimate. Individual cap and stem dry masses were unavailable, preventing mass-weighted reconstruction of paired fruiting bodies. The study also lacked taxon-specific moisture measurements, As and Cr speciation, and in vitro bioaccessibility data. Consequently, the dietary calculations rely on simplified assumptions regarding adult consumption, body weight, dry matter and complete bioaccessibility; thus, they constitute screening estimates rather than a probabilistic dietary risk assessment or a determination of food safety.

The environmental-context analysis was limited to eight overlapping parks, with only 3–23 reconstructed fruiting-body units and one to eight taxa contributing to the mushroom summary for each park. The marked differences in taxonomic composition among parks, together with the use of homogenised park-level soil composites rather than soil collected from the immediate fruiting-body microsites, limited both statistical power and biological attribution. Although exact permutation tests, false-discovery-rate corrections and leave-one-park-out analyses were used, the resulting correlations remained exploratory and cannot establish prediction, individual uptake or mechanistic soil-to-mushroom transfer.

Although the contextual soil dataset was supported by two soil reference materials, CRM059 and NCS DC87101, the latter showed element-specific limitations, including reference-only Cd, oxide-based Fe and low Zn recovery. Soil–mushroom correlations were therefore interpreted cautiously and only at park/area level. For the mushroom matrix, NIST SRM 1570a provided botanical-matrix validation for several elements, but not for Be or Cr; this limitation is particularly relevant for Be, which was almost entirely censored and was excluded from the main PCA.

A further analytical limitation was that collision/reaction modes were not operational during ICP-MS analysis. Although tuning performance, external calibration, procedural blanks, triplicate acquisition and reference-material recoveries supported the general analytical workflow, they could not fully exclude matrix-dependent polyatomic interferences. The Cr results, particularly those based on ^52^Cr, therefore carry additional uncertainty, while As and V require similar but more moderate caution. The isolated extreme records also could not be retrospectively confirmed using individual triplicate readings or sample-specific carryover checks and were consequently not interpreted as definitive evidence of biological enrichment.

Remaining frozen mushroom material is available and could support future speciation or in vitro bioaccessibility studies, subject to sample integrity and sufficient residual mass. Aliquots of the archived digests also remain available, but these could be used only to verify total-element concentrations; they are not suitable for retrospective speciation or bioaccessibility testing.

## 5. Conclusions

Wild mushrooms from Leicester/Leicestershire exhibited heterogeneous multielement profiles influenced by taxon, tissue basis, censoring pattern and upper-tail elemental concentrations. Cu, Mn and Zn were detected in all reconstructed fruiting-body-level units, Cd and Pb were broadly quantifiable, whereas Be was almost entirely censored and was not interpreted distributionally. Reconstruction of the main dataset at fruiting-body level avoided double counting of paired cap/stem records and provided a more robust basis for descriptive interpretation, multivariate analysis and adult exposure screening.

The primary PCA, restricted to Cd, Cu, Mn, Pb, V and Zn, explained 73.92% of the total variance across PC1–PC3. PC1 was associated mainly with Pb, V and Mn, PC2 with Zn, Cu and Cd, and PC3 was dominated by Cd. The overall PCA score configuration remained stable across alternative treatments of censored observations and after exclusion of the flagged extreme records, although the precise magnitude and ordering of some PC1 and PC2 loadings showed moderate sensitivity. The broader ten-element PCA excluding Be and the full PCA including Be were therefore retained only as supplementary sensitivity or diagnostic analyses because of censoring and analytical uncertainty. Collectively, these results support the presence of broad multielement fingerprints but do not identify a single contamination gradient, environmental source or predictive exposure model.

Adult exposure screening identified Cd as the principal threshold-based driver in the pooled edible-relevant reference population, corresponding to 47.33% of the EFSA TWI under the 300 g fresh-weight week^−1^ scenario. Taxon-specific screening nevertheless altered both the magnitude and ranking of the principal drivers. The corresponding Cd estimate reached 50.88% of the TWI for *Agaricus bitorquis*, whereas Cu was the principal threshold-based driver for *Coprinopsis atramentaria* and *Marasmius oreades*. Paired tissue analysis in *A. bitorquis* also showed higher cap than stem concentrations for Cd, Cu, Pb and Zn. These findings support the taxon- and tissue-specific component of the working hypothesis, while demonstrating that the pooled edible-relevant population did not represent all individual taxa equally.

The As and Pb results require conservative and speciation-aware interpretation, and the dietary calculations should be regarded as screening estimates rather than determinations of food safety. The separate hypothetical-ingestion assessment for non-edible, uncertain-edibility, toxic and unidentified taxa, including *Panaeolina foenisecii*, remained essential because metal-based calculations do not capture intrinsic mushroom toxicity, psychoactive potential, gastrointestinal effects, misidentification risk or other non-metal hazards.

After harmonisation of the park identifiers, the environmental-context analysis included eight overlapping parks. None of the six primary same-element correlations for Cd, Cu, Mn, Pb, V and Zn, or the broader cross-element and soil-physicochemical associations, remained statistically significant after the applicable false-discovery-rate corrections. The leave-one-park-out results were also sensitive to the omitted park. The soil data therefore provided useful environmental context but no statistically supported evidence of prediction, individual uptake or mechanistic soil-to-mushroom transfer.

Overall, the working hypothesis was partially supported. Fruiting-body-level reconstruction, taxon- and tissue-aware interpretation, prespecified censored-data rules and sensitivity-tested multivariate analysis improved identification of exposure-relevant patterns, whereas the park-level environmental associations were not supported as robust predictors. This framework provides a transparent basis for integrating multielement mushroom data and adult exposure-screening scenarios without overinterpreting censored observations, upper-tail records or contextual soil measurements. Future studies should prioritise balanced taxon-specific sampling, microsite-matched substrates, measured moisture content, elemental speciation, bioaccessibility testing and analytically targeted confirmation of extreme observations.

## Figures and Tables

**Table 1:** Overall metal(loid) concentrations in wild mushroom fruiting-body-level units from Leicestershire, UK (mg kg^−1^ dry weight).

Element	*N*	*N* Detected	Censoring (%)	LoD	Detected Range	Central Estimate	IQR	P95	Basis
As	121	37	69.42	0.309	0.326–3.689	0.233	0.093–0.458	/	ROS, exploratory
Be	121	1	99.17	0.185	0.285–0.285	/	/	/	Not estimable
Cd	121	117	3.31	0.081	0.102–101.054	1.856	0.573–2.935	5.310	ROS
Co	121	34	71.90	0.104	0.104–12.745	0.016	0.003–0.115	/	ROS, exploratory
Cr	121	70	42.15	1.012	1.109–52.649	1.356	0.490–2.283	10.082	ROS
Cu	121	121	0.00	2.224	7.885–5956.478	71.142	37.391–102.534	194.022	Observed
Mn	121	121	0.00	0.529	2.291–2676.159	14.318	8.322–42.684	350.634	Observed
Ni	121	64	47.11	3.400	3.724–1387.741	3.874	0.912–9.092	50.935	ROS
Pb	121	106	12.40	0.872	1.026–41.355	2.171	1.545–3.868	8.669	ROS
V	121	84	30.58	0.062	0.063–15.299	0.104	0.033–0.261	1.314	ROS
Zn	121	121	0.00	5.335	29.646–3064.543	97.506	64.854–133.969	907.435	Observed

*N* detected was defined using the reconstructed fruiting-body-level detection rule. Detected ranges include detected observations only. For uncensored elements (Cu, Mn and Zn), the observed median, IQR and P95 are reported. For censored elements meeting the predefined criteria, the median, IQR and P95 were estimated from the complete distribution modelled using NADA::cenros. As and Co estimates are exploratory because of high censoring. Be, with 1/121 detections, was not estimable and is reported as detection-frequency/upper-bound information only. P95 was reported only when all prespecified eligibility criteria were met: *n* ≥ 20, at least 10 detected observations, censoring <50%, availability of a valid observed or convergent ROS distribution, and 2000 valid nonparametric bootstrap estimates. When any criterion was not met, P95 was not reported. LoD, limit of detection; ROS, regression on order statistics; /, not reported.

**Table 2:** Taxon-level metal(loid) concentrations in fruiting-body-level units for retained wild mushroom taxa (mg kg^−1^ dry weight).

Taxon	Element	*N* Detected	Fully Censored (%)	LoD	Detected Range	Central Estimate	IQR	P95	Basis
*Agaricus bitorquis* (*n* = 22)	As	0	100	0.309	/	/	/	/	Not estimable
	Be	1	95.45	0.185	0.285–0.285	/	/	/	Not estimable
	Cd	22	0	0.081	2.127–4.846	2.968	2.813–3.385	4.230	Observed median
	Co	4	81.82	0.104	0.160–0.246	/	/	/	Not estimable
	Cr	8	63.64	1.012	1.134–17.920	0.318	0.068–1.510	/	ROS, exploratory
	Cu	22	0	2.224	87.453–142.878	105.603	99.623–126.178	132.024	Observed median
	Mn	22	0	0.529	7.321–15.298	9.983	9.233–11.522	12.644	Observed median
	Ni	5	77.27	3.400	4.922–15.794	1.259	0.536–2.934	/	ROS, exploratory
	Pb	22	0	0.872	1.397–4.141	2.082	1.872–2.593	3.390	Observed median
	V	16	27.27	0.062	0.066–0.194	0.083	0.058–0.096	0.174	ROS
	Zn	22	0	5.335	54.802–90.282	63.363	59.535–67.416	84.477	Observed median
*Coprinopsis atramentaria* (*n* = 8)	As	7	12.50	0.309	0.386–1.364	0.784	0.417–0.929	/	ROS
	Be	0	100	0.185	/	/	/	/	Not estimable
	Cd	5	37.50	0.081	0.172–2.312	0.183	0.035–0.489	/	ROS
	Co	6	25	0.104	0.117–1.080	0.150	0.097–0.471	/	ROS
	Cr	6	25	1.012	2.033–11.419	4.141	1.835–6.673	/	ROS
	Cu	8	0	2.224	29.042–330.580	80.824	55.135–125.430	/	Observed median
	Mn	8	0	0.529	12.068–207.495	40.493	18.361–123.952	/	Observed median
	Ni	8	0	3.400	3.825–169.724	10.872	7.030–58.873	/	ROS
	Pb	8	0	0.872	1.985–10.569	6.621	4.374–8.062	/	Observed median
	V	8	0	0.062	0.175–4.338	0.422	0.205–1.693	/	Observed median
	Zn	8	0	5.335	87.909–149.401	109.423	100.579–120.859	/	Observed median
*Marasmius oreades* (*n* = 5)	As	1	80	0.309	1.313–1.313	/	/	/	Not estimable
	Be	0	100	0.185	/	/	/	/	Not estimable
	Cd	5	0	0.081	0.122–2.034	0.277	0.123–0.313	/	Observed median
	Co	3	40	0.104	0.149–0.292	0.149	0.094–0.251	/	ROS
	Cr	1	80	1.012	1.317–1.317	/	/	/	Not estimable
	Cu	5	0	2.224	27.383–83.318	62.809	49.000–68.810	/	Observed median
	Mn	5	0	0.529	7.745–262.264	14.318	11.944–21.601	/	Observed median
	Ni	2	60	3.400	3.874–4.776	/	/	/	Not estimable
	Pb	4	20	0.872	1.250–2.104	1.251	1.250–2.099	/	ROS
	V	4	20	0.062	0.068–0.852	0.192	0.068–0.417	/	ROS
	Zn	5	0	5.335	53.469–122.701	76.631	69.631–96.922	/	Observed median
*Mycena citrinomarginata* (*n* = 22)	As	2	90.91	0.309	0.419–1.881	/	/	/	Not estimable
	Be	0	100	0.185	/	/	/	/	Not estimable
	Cd	22	0	0.081	0.183–2.208	0.556	0.449–0.676	1.718	Observed median
	Co	8	63.64	0.104	0.112–11.943	0.015	0.002–0.172	/	ROS, exploratory
	Cr	19	13.64	1.012	1.194–10.656	1.997	1.295–3.326	5.723	ROS
	Cu	22	0	2.224	29.208–5956.478	50.781	41.209–137.339	851.426	Observed median
	Mn	22	0	0.529	21.154–217.163	29.032	26.231–31.653	47.327	Observed median
	Ni	20	9.09	3.400	3.778–1387.741	11.796	5.781–22.605	75.154	ROS
	Pb	19	13.64	0.872	1.036–20.689	3.012	1.379–4.864	19.259	ROS
	V	21	4.55	0.062	0.065–5.939	0.211	0.157–0.499	1.410	ROS
	Zn	22	0	5.335	93.710–295.003	112.666	102.832–123.393	195.722	Observed median
*Panaeolina foenisecii* (*n* = 16)	As	3	81.25	0.309	0.328–0.472	/	/	/	Not estimable
	Be	0	100	0.185	/	/	/	/	Not estimable
	Cd	15	6.25	0.081	0.197–13.446	2.566	1.726–3.333	/	ROS
	Co	2	87.50	0.104	0.104–0.131	/	/	/	Not estimable
	Cr	7	56.25	1.012	1.306–2.680	1.105	0.807–1.560	/	ROS, exploratory
	Cu	16	0	2.224	19.747–129.366	57.788	32.580–79.632	/	Observed median
	Mn	16	0	0.529	6.474–699.757	162.809	11.298–335.020	/	Observed median
	Ni	11	31.25	3.400	4.387–26.420	5.515	2.722–8.517	/	ROS
	Pb	16	0	0.872	1.054–8.179	3.895	2.363–4.641	/	Observed median
	V	9	43.75	0.062	0.091–1.098	0.112	0.035–0.238	/	ROS
	Zn	16	0	5.335	54.889–161.629	86.031	64.456–127.714	/	Observed median
*Pholiota adiposa* (*n* = 5)	As	2	60	0.309	0.570–0.900	/	/	/	Not estimable
	Be	0	100	0.185	/	/	/	/	Not estimable
	Cd	5	0	0.081	1.404–101.054	2.520	2.256–35.717	/	Observed median
	Co	3	40	0.104	0.114–12.745	0.114	0.005–4.486	/	ROS
	Cr	3	40	1.012	1.114–52.649	1.114	0.079–19.466	/	ROS
	Cu	5	0	2.224	24.891–460.309	60.954	29.861–161.472	/	Observed median
	Mn	5	0	0.529	9.496–2676.159	183.960	140.300–952.135	/	Observed median
	Ni	3	40	3.400	6.716–68.568	6.716	1.625–56.585	/	ROS
	Pb	5	0	0.872	1.654–8.669	1.783	1.778–6.922	/	ROS
	V	3	40	0.062	0.091–15.299	0.091	0.003–5.528	/	ROS
	Zn	5	0	5.335	53.575–3064.543	84.631	71.390–1052.542	/	Observed median
*Psathyrella fatua* (*n* = 6)	As	3	50	0.309	0.326–1.593	0.229	0.084–1.033	/	ROS, exploratory
	Be	0	100	0.185	/	/	/	/	Not estimable
	Cd	6	0	0.081	0.102–2.391	1.490	0.795–1.783	/	Observed median
	Co	2	66.67	0.104	0.176–0.310	/	/	/	Not estimable
	Cr	5	16.67	1.012	1.317–2.482	1.638	1.375–2.144	/	ROS
	Cu	6	0	2.224	20.914–34.018	30.860	27.782–32.896	/	Observed median
	Mn	6	0	0.529	7.916–303.474	90.846	13.194–252.807	/	Observed median
	Ni	2	66.67	3.400	4.395–4.590	/	/	/	Not estimable
	Pb	6	0	0.872	1.535–3.097	2.191	2.036–2.438	/	Observed median
	V	4	33.33	0.062	0.103–0.727	0.268	0.070–0.561	/	ROS
	Zn	6	0	5.335	41.385–130.487	54.002	46.552–66.767	/	Observed median
Unknown/Unidentified taxon (*n* = 17)	As	17	0	0.309	0.623–3.689	1.941	1.277–2.546	/	Observed median
	Be	0	100	0.185	/	/	/	/	Not estimable
	Cd	17	0	0.081	1.290–5.311	2.421	1.824–4.065	/	Observed median
	Co	1	94.12	0.104	0.213–0.213	/	/	/	Not estimable
	Cr	15	11.76	1.012	1.109–3.019	1.738	1.467–2.564	/	ROS
	Cu	17	0	2.224	30.950–194.022	72.288	57.573–88.318	/	Observed median
	Mn	17	0	0.529	2.291–15.055	5.214	3.969–6.036	/	Observed median
	Ni	6	64.71	3.400	3.724–12.304	2.002	0.983–3.943	/	ROS, exploratory
	Pb	12	29.41	0.872	1.026–8.053	1.641	0.723–2.171	/	ROS
	V	11	35.29	0.062	0.065–0.738	0.100	0.034–0.133	/	ROS
	Zn	17	0	5.335	203.310–1375.726	475.986	301.291–907.435	/	Observed median

Taxon sample sizes are given in parentheses. *N* detected was defined using the reconstructed fruiting-body-level detection rule. Fully censored (%) denotes fruiting-body-level units for which none of the contributing analytical component records was detected. Detected ranges include detected observations only. Central estimates are observed medians for uncensored groups or NADA::cenros medians for censored groups meeting the prespecified eligibility criteria: at least three detected observations, censoring <80%, technical compatibility of the observed values and censoring limits, and convergence of the ROS fit. ROS estimates for groups with ≥50% but <80% censoring are labelled exploratory. Groups with fewer than three detections or ≥80% censoring were classified as not estimable. P95 was reported only when all prespecified eligibility criteria were met: *n* ≥ 20, at least 10 detected observations, censoring <50%, availability of a valid observed or convergent ROS distribution, and 2000 valid nonparametric bootstrap estimates. When any criterion was not met, P95 was not reported. LoD, limit of detection; ROS, regression on order statistics; /, not estimable or not reported.

**Table 3:** Paired cap–stem comparison and reconstruction sensitivity for *Agaricus bitorquis* (*n* = 22 paired fruiting bodies). Panel A: Paired cap–stem concentrations and Wilcoxon signed-rank tests. Panel B: Sensitivity of reconstructed whole-fruiting-body concentrations to hypothetical cap:stem dry-mass contributions.

Element	*n* Paired	Officially Detected Units	Fully Censored Units	Cap Median (mg kg^−1^ dw)	Stem Median (mg kg^−1^ dw)	Cap/Stem Ratio	Wilcoxon *p*	FDR-Adjusted *p*	Direction
Cd	22	22	0	3.878	2.068	1.87	<0.001	<0.001	cap > stem
Cu	22	22	0	114.963	93.365	1.23	<0.001	<0.001	cap > stem
Mn	22	22	0	10.541	9.617	1.10	0.144	0.198	cap > stem
Pb	22	22	0	2.462	1.580	1.56	0.002	0.004	cap > stem
V	22	16	6	0.062	0.069	0.89	0.436	0.533	stem ≥ cap
Zn	22	22	0	75.369	47.549	1.59	<0.001	<0.001	cap > stem
**Element**		**30:70 Cap:Stem** **(mg kg^−1^ dw)**		**50:50 Cap:Stem** **(mg kg^−1^ dw)**		**70:30 Cap:Stem** **(mg kg^−1^ dw)**		**Direction of Potential Bias**	
Cd		2.574		2.968		3.348		For cap-enriched elements, the 50:50 reconstruction may underestimate the whole-fruiting-body concentration when the cap dry-mass fraction exceeds 50%; the reverse applies to stem-enriched elements.	
Cu		101.824		105.603		110.996			
Mn		9.668		9.983		10.152			
Pb		1.954		2.082		2.216			
V		0.080		0.083		0.078			
Zn		56.926		63.363		68.502			

Paired comparisons used the Wilcoxon signed-rank test; *p*-values were adjusted using the Benjamini–Hochberg false-discovery-rate procedure. For V, six reconstructed units were fully censored, and cap/stem medians use LoD-capped working values; the remaining elements in Panel A were detected in all 22 units. The 30:70, 50:50 and 70:30 scenarios are hypothetical dry-mass contributions because original cap and stem masses were unavailable. They quantify reconstruction sensitivity and do not estimate the true tissue proportions.

**Table 4:** Central-estimate adult dietary-screening summary for the pooled edible-relevant reference population and individual edible or edible-with-caution wild mushroom taxa.

Edibility Category	Taxon	Scenario (g FW Week^−1^)	As MOE	Pb MOE	Cd % TWI	Cu % Safe Level	Mn % Safe Level	Ni % TDI	V % MRL	Zn % UL	Main Threshold-Based Driver	Lowest MOE
Pooled reference	Pooled edible-relevant (*N* = 43)	100	26.90 †	11.64	15.78	2.90	0.19	0.21 †	0.02	0.40	Cd (15.78%)	Pb (11.64)
Pooled reference	Pooled edible-relevant (*N* = 43)	300	8.97 †	3.88	47.33	8.70	0.56	0.63 †	0.06	1.19	Cd (47.33%)	Pb (3.88)
Edible	*Agaricus arvensis*	100	—	—	3.24	0.94	0.09	—	—	0.32	Cd (3.24%)	—
Edible	*Agaricus arvensis*	300	—	—	9.73	2.82	0.26	—	—	0.95	Cd (9.73%)	—
Edible	*Agaricus bitorquis*	100	—	11.77	16.96	3.08	0.18	0.20	0.02	0.36	Cd (16.96%)	Pb (11.77)
Edible	*Agaricus bitorquis*	300	—	3.92	50.88	9.24	0.53	0.59	0.05	1.09	Cd (50.88%)	Pb (3.92)
Edible	*Agaricus nivescens*	100	—	—	3.02	2.88	0.16	—	—	0.57	Cd (3.02%)	—
Edible	*Agaricus nivescens*	300	—	—	9.05	8.65	0.49	—	—	1.72	Cd (9.05%)	—
Edible with caution	*Coprinopsis atramentaria*	100	3.75	3.70	1.05	2.36	0.72	1.71	0.09	0.63	Cu (2.36%)	Pb (3.70)
Edible with caution	*Coprinopsis atramentaria*	300	1.25	1.23	3.14	7.07	2.17	5.12	0.26	1.88	Cu (7.07%)	Pb (1.23)
Edible with caution	*Leucoagaricus leucothites*	100	—	—	6.65	0.41	0.13	—	0.04	0.17	Cd (6.65%)	—
Edible with caution	*Leucoagaricus leucothites*	300	—	—	19.96	1.24	0.40	—	0.11	0.51	Cd (19.96%)	—
Edible	*Marasmius oreades*	100	—	19.58	1.58	1.83	0.26	—	0.04	0.44	Cu (1.83%)	Pb (19.58)
Edible	*Marasmius oreades*	300	—	6.53	4.75	5.49	0.77	—	0.12	1.31	Cu (5.49%)	Pb (6.53)
Edible with caution	*Vascellum pratense*	100	—	12.63	12.24	1.83	2.28	—	—	0.55	Cd (12.24%)	Pb (12.63)
Edible with caution	*Vascellum pratense*	300	—	4.21	36.71	5.49	6.84	—	—	1.66	Cd (36.71%)	Pb (4.21)

Adult screening assumptions: body weight = 70 kg; dry-matter fraction = 0.10; consumption = 100 or 300 g fresh weight per week; 100% bioaccessibility. As and Pb are expressed as margins of exposure (MOE), for which lower values indicate greater potential concern. Other elements are expressed as percentages of the benchmark indicated. — = indicates that no defensible central estimate or quantitative screening value was available. Be, Co and Cr were excluded from the primary quantitative screening. The pooled edible-relevant reference population comprised 43 reconstructed fruiting-body-level units classified as edible or edible with caution and was included solely as an analytical comparator for evaluation of the working hypothesis; it should not be interpreted as a balanced population estimate. † Exploratory ROS estimate because censoring was ≥50% but <80%. *Vascellum pratense* was classified as edible with caution because edibility depends on consumption of immature specimens with a white internal gleba.

**Table 5:** Central-estimate hypothetical-ingestion screening for non-edible, toxic, uncertain-edibility and unidentified taxa.

Edibility Category	Taxon	Scenario (g FW Week^−1^)	As MOE	Pb MOE	Cd % TWI	Cu % Safe Level	Mn % Safe Level	Ni % TDI	V % MRL	Zn % UL	Main Threshold-Based Driver	Lowest MOE
Inedible	*Armillaria mellea*	100	—	—	9.04	0.37	0.12	—	—	0.19	Cd (9.04%)	—
Inedible	*Armillaria mellea*	300	—	—	27.11	1.12	0.37	—	—	0.57	Cd (27.11%)	—
Toxic	*Hypholoma fasciculare*	100	—	21.67	1.99	2.61	0.31	—	0.06	0.59	Cu (2.61%)	Pb (21.67)
Toxic	*Hypholoma fasciculare*	300	—	7.22	5.96	7.83	0.93	—	0.18	1.76	Cu (7.83%)	Pb (7.22)
Unknown	*Mycena citrinomarginata*	100	—	8.14	3.18	1.48	0.52	1.85	0.04	0.64	Cd (3.18%)	Pb (8.14)
Unknown	*Mycena citrinomarginata*	300	—	2.71	9.53	4.44	1.56	5.56	0.13	1.93	Cd (9.53%)	Pb (2.71)
Non-edible/uncertain-edibility	*Panaeolina foenisecii*	100	—	6.29	14.66	1.68	2.91	0.87	0.02	0.49	Cd (14.66%)	Pb (6.29)
Non-edible/uncertain-edibility	*Panaeolina foenisecii*	300	—	2.10	43.99	5.05	8.72	2.60	0.07	1.47	Cd (43.99%)	Pb (2.10)
Toxic	*Pholiota adiposa*	100	—	13.74	14.40	1.78	3.28	1.05	0.02	0.48	Cd (14.40%)	Pb (13.74)
Toxic	*Pholiota adiposa*	300	—	4.58	43.19	5.33	9.85	3.16	0.06	1.45	Cd (43.19%)	Pb (4.58)
Mixed non-edible/toxic	*Pholiota gummosa*	100	—	3.45	6.94	1.87	1.15	3.27	—	0.57	Cd (6.94%)	Pb (3.45)
Mixed non-edible/toxic	*Pholiota gummosa*	300	—	1.15	20.82	5.60	3.44	9.81	—	1.71	Cd (20.82%)	Pb (1.15)
Mixed toxic/unknown	*Psathyrella corrugis*	100	—	6.84	5.32	2.02	2.55	—	—	0.51	Cd (5.32%)	Pb (6.84)
Mixed toxic/unknown	*Psathyrella corrugis*	300	—	2.28	15.97	6.05	7.66	—	—	1.53	Cd (15.97%)	Pb (2.28)
Mixed toxic/unknown	*Psathyrella fatua*	100	12.86	11.18	8.52	0.90	1.62	—	0.05	0.31	Cd (8.52%)	Pb (11.18)
Mixed toxic/unknown	*Psathyrella fatua*	300	4.29	3.73	25.55	2.70	4.87	—	0.16	0.93	Cd (25.55%)	Pb (3.73)
Mixed non-edible/toxic	*Psathyrella sublatispora*	100	—	1.13	19.49	1.97	6.44	—	—	0.75	Cd (19.49%)	Pb (1.13)
Mixed non-edible/toxic	*Psathyrella sublatispora*	300	—	0.38	58.46	5.90	19.33	—	—	2.26	Cd (58.46%)	Pb (0.38)
Unknown	Unknown/Unidentified taxon	100	1.51	14.93	13.84	2.11	0.09	0.31	0.02	2.72	Cd (13.84%)	As (1.51)
Unknown	Unknown/Unidentified taxon	300	0.50	4.98	41.51	6.32	0.28	0.94	0.06	8.16	Cd (41.51%)	As (0.50)
Unknown	*Xerocomus porosporus*	100	—	—	30.93	2.92	0.17	—	—	0.49	Cd (30.93%)	—
Unknown	*Xerocomus porosporus*	300	—	—	92.80	8.75	0.52	—	—	1.47	Cd (92.80%)	—

These calculations are hypothetical metal-based ingestion scenarios only and do not imply edibility or food safety. They do not account for intrinsic fungal toxins, psychoactive compounds, gastrointestinal toxicity, misidentification, preparation-related hazards or other non-metal risks. — = indicates that no defensible central estimate or quantitative screening value was available. Be, Co and Cr were excluded from the primary quantitative screening; Cr(VI)-equivalent estimates are reported only in a supplementary analysis based on arbitrary speciation-sensitivity scenarios.

## Data Availability

The data and reproducible analysis files supporting this study are publicly available in the Open Science Framework at https://doi.org/10.17605/OSF.IO/XBRD7. The repository contains the curated analytical datasets, censoring indicators, final derived tables, figure-source data and reproducible analysis files. The datasets are released under the Creative Commons Attribution 4.0 International licence (CC BY 4.0).

## Supplementary Materials

The following supporting information can be downloaded at: https://www.mdpi.com/article/10.3390/jox16040141/s1, Table S1: Data provenance and analytical inventory of the wild mushroom dataset (Panel A: data provenance and distinction from companion studies; Panel B: wild mushroom analytical inventory by taxon, edibility category, tissue basis and area type); Table S2: Toxicological benchmarks used for the final main dietary screening; Table S3: Contextual mixed-taxon tissue-basis summary for all original analytical mushroom units; Table S4: Same-taxon cap/stem summaries for tissue-separated taxa; Table S5: Metal(loid) concentrations by area type using fruiting-body-level units; Table S6: Metal(loid) concentrations by urban coordinate quadrant using fruiting-body-level units; Table S7: PCA reporting summary and sensitivity analyses for the reconstructed fruiting-body-level mushroom dataset (Panel A: primary, supplementary and diagnostic PCA designs; Panel B: primary-PCA sensitivity analyses); Table S8: Primary and exploratory park-level soil–mushroom and soil-physicochemical correlations based on the harmonised eight-park dataset; Table S9: Leave-one-park-out sensitivity analysis of the primary same-element park-level correlations based on the harmonised eight-park dataset; Table S10: Central and high-end dietary-screening scenarios at 300 g fresh weight week^−1^ and a dry-matter fraction of 0.10; Table S11: Sensitivity of dietary-screening estimates to assumed dry-matter fractions of 0.08, 0.10 and 0.12; Table S12: Supplementary hypothetical Cr(VI)-fraction sensitivity analysis; Figure S1: Sensitivity of the primary principal component analysis loadings to alternative treatments of censored observations; Figure S2: Sensitivity of the primary principal component analysis loadings to exclusion of flagged extreme fruiting-body records.
