# Supplementary material for: Censored-Data-Aware Screening of Hazardous Metal(loid)s in Wild Mushrooms from Urban and Peri-Urban Green Spaces: Multielement Fingerprints and Adult Exposure Assessment
Source: J Xenobiot. 2026 Jul 30;16(4):141. doi: 10.3390/jox16040141 (PMC13514479; doi:10.3390/jox16040141)

Supplementary Tamerials: Censored-Data-Aware Screening of Hazardous Metal(loid)s in Wild Mushrooms from Urban and Peri-Urban Green Spaces: Multielement Fingerprints and Adult Exposure Assessment

Antonio Peña-Fernández [1,2,*], M. Carmen Lobo [3], M. Ángeles Peña Fernández [4], Guillermo Torrado Durán [4], Mark D. Evans [2], Tiziana Sgamma [2], Tomás Cámara-Pastor [5] and Gurminderjeet S. Jagdev [2]

Figure S1. Sensitivity of the primary principal component analysis loadings to alternative treatments of censored observations. Aligned loadings for PC1, PC2 and PC3 from the primary six-element PCA (Cd, Cu, Mn, Pb, V and Zn; *N* = 121 reconstructed fruiting-body-level units) are compared across the LoD-capped reconstruction used in the primary analysis, LoD/2 substitution and ROS-based working estimates. Alternative censored-value treatments were applied to the original component records before reconstruction of paired cap/stem units. Concentrations were $\log_{10}$-transformed, centred and scaled to unit variance before PCA. Component signs were aligned with the primary solution because PCA loading signs are mathematically arbitrary. The broadly overlapping profiles indicate that the principal loading structure was retained across censored-value treatments, although modest differences in loading magnitude were observed. LoD-capped, LoD/2-based and ROS-derived values represent analytical working estimates and should not be interpreted as directly measured concentrations for censored observations. LoD, limit of detection; ROS, regression on order statistics.

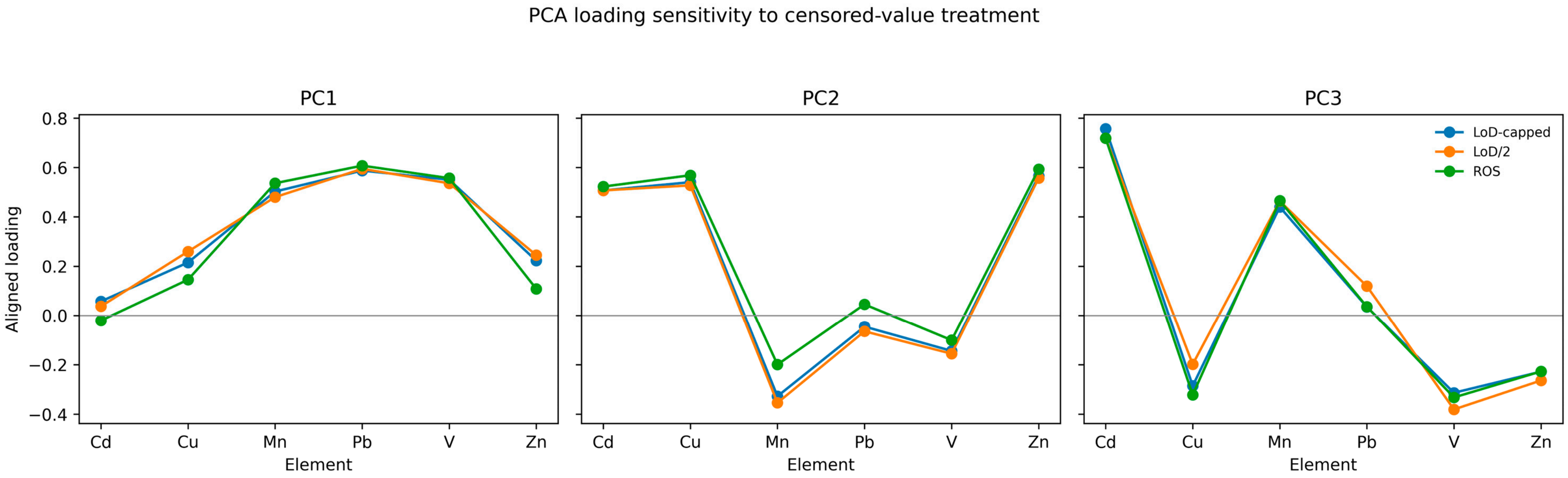

Figure S2. Sensitivity of the primary principal component analysis loadings to the exclusion of flagged extreme fruiting-body records. Aligned loadings for PC1, PC2 and PC3 from the primary six-element PCA (Cd, Cu, Mn, Pb, V and Zn; $N = 121$) are compared with analyses excluding the Cu-extreme record FB070 ($N = 120$), excluding the multielement-extreme record FB025 ($N = 120$), or excluding both records ($N = 119$). FB025 corresponds to a *Pholiota adiposa* fruiting-body-level unit collected from Abbey Park that showed an extreme multielement profile, particularly for Cd, Mn, V and Zn. FB070 corresponds to a *Mycena citrinomarginata* unit collected from Victoria Park that showed an extreme Cu concentration. The corresponding source records can be verified in the fruiting-body-level mushroom dataset deposited in the OSF repository by filtering the record-identifier field for FB025 and FB070. Concentrations were log_{10}-transformed, centred and scaled to unit variance before PCA. Component signs were aligned with the primary solution because PCA loading signs are mathematically arbitrary. The overall element groupings were retained after the exclusions, but the magnitude and relative ordering of selected PC1 and PC2 loadings showed greater sensitivity when the multielement-extreme record FB025 was removed, either alone or jointly with FB070. PC3 was comparatively more stable. Both records were retained in the primary analysis because no objective analytical exclusion criterion was available; the exclusion analyses were performed solely to evaluate their influence on the multivariate interpretation.

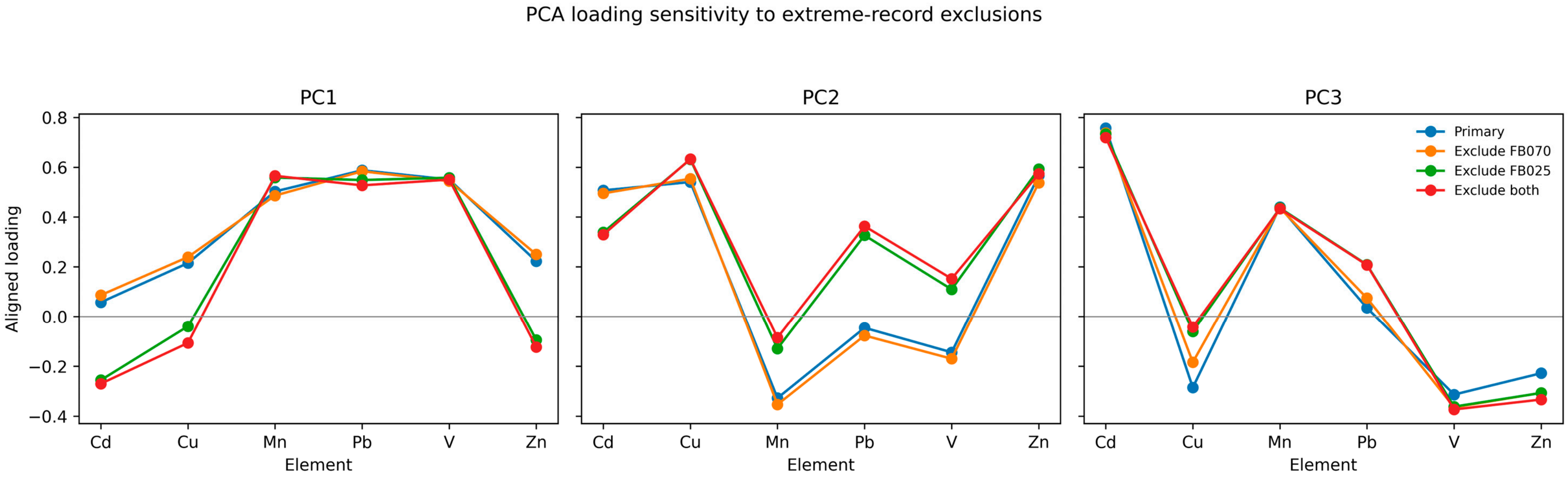

Supplement: Supplementary file 1 [file jox-16-00141-s001.zip › jox-4416931 SM-figure.pdf]
