# Supplementary material for: Censored-Data-Aware Screening of Hazardous Metal(loid)s in Wild Mushrooms from Urban and Peri-Urban Green Spaces: Multielement Fingerprints and Adult Exposure Assessment
Source: J Xenobiot. 2026 Jul 30;16(4):141. doi: 10.3390/jox16040141 (PMC13514479; doi:10.3390/jox16040141)
Supplement: Supplementary file 1 [file jox-16-00141-s001.zip › jox-4416931 SM-table.pdf]

Supplementary Tamerials: Censored-Data-Aware Screening of Hazardous Metal(loid)s in Wild Mushrooms from Urban and Peri-Urban Green Spaces: Multielement Fingerprints and Adult Exposure Assessment

Antonio Peña-Fernández [1,2,*], M. Carmen Lobo [3], M. Ángeles Peña Fernández [4], Guillermo Torrado Durán [4], Mark D. Evans [2], Tiziana Sgamma [2], Tomás Cámara-Pastor [5] and Gurminderjeet S. Jagdev [2]

Supplementary Table S1. Data provenance and analytical inventory of the wild mushroom dataset.

Study	Shared source data	Primary analytical unit and scope	Distinct contribution
Previous Cd–Cu–Zn mushroom–soil study [12]	Cd, Cu and Zn determinations from the same analytical campaign; 153 original analytical component records contributing to the 121 reconstructed fruiting-body-level units used in the present study.	Original whole-fruiting-body and tissue-separated cap/stem records; emphasis on spatial distribution, species- and tissue-related accumulation and apparent area-level soil-to-fungus transfer.	Focused specifically on Cd, Cu and Zn and their spatial and soil–fungus context.
Present mushroom study	The same Cd, Cu and Zn determinations were reprocessed together with As, Be, Co, Cr, Mn, Ni, Pb and V.	121 reconstructed fruiting-body-level units; paired cap/stem records retained separately for tissue analysis; adult dietary screening restricted to reconstructed edible and edible-with-caution units.	Eleven-element, censored-data-aware profiling; restricted primary PCA; taxon- and tissue-aware interpretation; and adult exposure screening.
Companion topsoil study [13]	Contextual soil measurements from the same wider Leicester/Leicestershire field framework.	26 site-level topsoil composites derived from 850 field increments; 102 analytical/subsampling observations used for analytical characterisation and QA/QC.	Soil physicochemical characterisation, geochemical interpretation, enrichment assessment and soil-related screening; these analyses are not repeated in the present manuscript.

Taxon	Edibility category	Fruiting-body-level *N*	Whole-fruiting-body units	Paired cap/stem units	Single-tissue units	Urban inventory *N*	Rural inventory *N*
Agaricus arvensis	Edible	2	0	2	0	2	0
Agaricus bitorquis	Edible	22	0	22	0	22	0
Agaricus nivescens	Edible	1	0	1	0	1	0
Armillaria mellea	Inedible	3	0	3	0	3	0
Coprinopsis atramentaria	Edible with caution	8	6	2	0	0	8

Study		Shared source data		Primary analytical unit and scope		Distinct contribution	
Hypholoma fasciculare	Toxic	1	1	0	0	1	0
Leucoagaricus leucothites	Edible with caution	1	1	0	0	1	0
Macrolepiota procera	Edible	0	0	0	3	0	3
Marasmius oreades	Edible	5	5	0	0	5	0
Mycena citrinomarginata	Unknown	22	22	0	0	22	0
Panaeolina foenisecii	Non-edible/uncertain-edibility	16	16	0	0	16	0
Pholiota adiposa	Toxic	5	4	1	0	5	0
Pholiota gummosa	Mixed non-edible/toxic	2	2	0	0	2	0
Psathyrella corrugis	Mixed toxic/unknown	2	2	0	0	2	0
Psathyrella fatua	Mixed toxic/unknown	6	6	0	0	6	0
Psathyrella sublatispora	Mixed non-edible/toxic	2	2	0	0	2	0
Unknown/Unidentified taxon	Unknown	17	17	0	1	18	0
Vascellum pratense	Edible with caution	4	4	0	0	4	0
Xerocomus porosporus	Unknown	2	1	1	0	2	0

Panel A. Data provenance and distinction from companion studies.

Cd, Cu and Zn are not presented in the current manuscript as newly generated determinations. Their reuse reflects reprocessing within a distinct analytical unit, expanded element panel and research question. The present soil–mushroom analysis uses only harmonised park-level summaries as complementary environmental context and does not repeat the companion topsoil geochemical or health-screening analyses.

Panel B. Wild mushroom analytical inventory by taxon, edibility category, tissue basis and area type.

Fruiting-body-level *N* equals whole-fruiting-body units plus paired cap/stem units and is the basis of the main fruiting-body-level descriptive tables. Paired cap/stem units correspond to complete cap/stem pairs reconstructed as one fruiting-body-level unit. Urban and rural inventory *N* values include the four single-tissue units; consequently, area inventory totals sum to 125 analytical units rather than 121 reconstructed fruiting-body-level units.

Table S2. Toxicological benchmarks used for the final main dietary screening.

Element	Benchmark type	Reference value	Application in this study	Output	Reference
As	Reference point / MOE	0.06 μg kg^{-1} bw day^{-1}	Total As treated conservatively as inorganic As for screening only.	MOE	[42]
Cd	TWI	2.5 μg kg^{-1} bw $week^{-1}$	EFSA tolerable weekly intake for cadmium.	% TWI	[20]
Cu	EFSA safe level	0.07 mg kg^{-1} bw day^{-1} (approximately 5 mg day^{-1} for a 70 kg adult)	Weekly intake compared with the daily safe level × 7.	% safe level	[44]
Mn	EFSA safe level	8 mg day^{-1} for adults	Weekly intake compared with 8 mg day^{-1} × 7; this is not a formal UL.	% safe level	[45]
Ni	TDI	13 μg kg^{-1} bw day^{-1}	EFSA tolerable daily intake for nickel.	% TDI	[43]
Pb	$BMDL_{01}$ / MOE	0.50 μg kg^{-1} bw day^{-1}	Developmental-neurotoxicity reference point; evaluated using MOE.	MOE	[21]
V	ATSDR oral MRL	0.01 mg kg^{-1} bw day^{-1}	ATSDR minimal risk level; not described as an EPA/IRIS RfD.	% MRL-equivalent	[46]
Zn	UL	25 mg day^{-1} for adults	Weekly intake compared with 25 mg day^{-1} × 7.	% UL	[47]

Be and Co were excluded from quantitative dietary screening. Total Cr was excluded from the main screening because speciation was not measured; Cr(VI)-equivalent values were retained only in a supplementary sensitivity analysis using arbitrary assumed fractions of total Cr (1%, 10%, 16.7% and 100%) and should not be interpreted as measured Cr(VI).

Table S3. Contextual mixed-taxon tissue-basis summary for all original analytical mushroom units.

Element	LoD	Tissue basis	N	N detected	Censoring (%)	Detected range	Central estimate	IQR	P95	Method
As	0.309	Cap	33	4	87.9	0.854-4.870	/	/	/	Not estimable
		Stem	35	3	91.4	0.968-1.535	/	/	/	Not estimable
		Whole fruiting body	89	34	61.8	0.326-3.689	0.302	0.126-0.957	/	Exploratory ROS median
Be	0.185	Cap	33	0	100.0	/	/	/	/	Not estimable
		Stem	35	1	97.1	0.386-0.386	/	/	/	Not estimable
		Whole fruiting body	89	0	100.0	/	/	/	/	Not estimable
Cd	0.081	Cap	33	33	0.0	0.216-6.598	3.696	2.740-4.016	5.668	Observed median
		Stem	35	35	0.0	0.094-3.679	1.929	0.877-2.177	2.820	Observed median
		Whole fruiting body	89	85	4.5	0.102-101.054	1.418	0.485-2.598	5.883	ROS median
Co	0.104	Cap	33	5	84.8	0.119-0.233	/	/	/	Not estimable
		Stem	35	5	85.7	0.115-0.276	/	/	/	Not estimable
		Whole fruiting body	89	28	68.5	0.104-12.745	0.018	0.003-0.149	/	Exploratory ROS median
Cr	1.012	Cap	33	5	84.8	1.216-18.152	/	/	/	Not estimable
		Stem	35	8	77.1	1.184-17.829	0.090	0.014-0.574	/	Exploratory ROS median
		Whole fruiting body	89	61	31.5	1.109-52.649	1.597	0.753-2.564	8.339	ROS median
Cu	2.224	Cap	33	33	0.0	10.639-366.376	111.423	106.463-133.062	191.301	Observed median
		Stem	35	35	0.0	2.715-294.785	89.258	74.429-106.786	175.953	Observed median
		Whole fruiting body	89	89	0.0	14.212-5956.478	57.656	34.577-87.233	196.973	Observed median
Mn	0.529	Cap	33	33	0.0	4.312-22.755	9.935	8.960-12.353	14.449	Observed median
		Stem	35	35	0.0	3.665-25.420	9.454	7.132-10.709	17.326	Observed median
		Whole fruiting body	89	89	0.0	2.291-2676.159	26.268	8.322-162.176	391.152	Observed median
Ni	3.400	Cap	33	8	75.8	4.251-20.797	1.387	0.580-3.293	/	Exploratory ROS median

Element	LoD	Tissue basis	N	N detected	Censoring (%)	Detected range	Central estimate	IQR	P95	Method
		Stem	35	7	80.0	6.444-14.784	/	/	/	Not estimable
		Whole fruiting body	89	53	40.4	3.724-1387.741	4.628	1.206-12.147	63.774	ROS median
Pb	0.872	Cap	33	28	15.2	1.806-9.735	2.400	2.045-3.042	6.717	ROS median
		Stem	35	28	20.0	0.988-5.544	1.502	1.014-1.888	3.760	ROS median
		Whole fruiting body	89	78	12.4	1.026-41.355	2.415	1.388-4.506	9.509	ROS median
V	0.062	Cap	33	13	60.6	0.084-0.320	0.069	0.044-0.127	/	Exploratory ROS median
		Stem	35	18	48.6	0.063-0.888	0.063	0.024-0.101	0.217	ROS median
		Whole fruiting body	89	65	27.0	0.063-15.299	0.158	0.045-0.432	2.269	ROS median
Zn	5.335	Cap	33	33	0.0	41.362-1174.286	76.761	73.226-94.556	157.777	Observed median
		Stem	35	35	0.0	7.876-125.712	48.013	44.069-53.960	95.352	Observed median
		Whole fruiting body	89	89	0.0	29.646-3064.543	111.359	84.631-161.629	971.688	Observed median

Concentrations are expressed as mg kg^{-1} dry weight. This table summarises original analytical units by tissue basis and therefore mixes taxa within cap, stem and whole-fruiting-body categories; it should not be interpreted as a same-species cap–stem comparison. Central estimates are observed medians for uncensored groups or NADA::cenros medians for censored groups with at least three detections and <80% censoring. ROS estimates with ≥50% censoring are labelled exploratory. P95 was reported only when all prespecified eligibility criteria were met: $n \geq 20$, at least 10 detected observations, censoring <50%, availability of a valid observed or convergent ROS distribution, and 2000 valid nonparametric bootstrap estimates. When any criterion was not met, P95 was not reported. Arithmetic and geometric means based on simple LoD substitution are omitted. / = not estimable or not reported.

Table S4. Same-taxon cap/stem summaries for tissue-separated taxa.

Taxon	Element	LoD	Tissue	N	N detected	Censoring (%)	Detected range	Central estimate	IQR	P95	Method
Agaricus arvensis	As	0.309	Cap	2	0	100.0	/	/	/	/	Not estimable
			Stem	2	0	100.0	/	/	/	/	Not estimable
	Be	0.185	Cap	2	0	100.0	/	/	/	/	Not estimable
			Stem	2	0	100.0	/	/	/	/	Not estimable
	Cd	0.081	Cap	2	2	0.0	0.216-1.446	0.831	0.524-1.139	/	Observed median
			Stem	2	2	0.0	0.094-0.513	0.304	0.199-0.408	/	Observed median
	Co	0.104	Cap	2	0	100.0	/	/	/	/	Not estimable
			Stem	2	0	100.0	/	/	/	/	Not estimable
	Cr	1.012	Cap	2	0	100.0	/	/	/	/	Not estimable
			Stem	2	0	100.0	/	/	/	/	Not estimable
	Cu	2.224	Cap	2	2	0.0	23.537-64.526	44.032	33.784-54.279	/	Observed median
			Stem	2	2	0.0	7.997-32.941	20.469	14.233-26.705	/	Observed median
	Mn	0.529	Cap	2	2	0.0	4.422-6.141	5.282	4.852-5.711	/	Observed median
			Stem	2	2	0.0	3.837-4.783	4.310	4.074-4.547	/	Observed median
	Ni	3.400	Cap	2	0	100.0	/	/	/	/	Not estimable
			Stem	2	1	50.0	14.784-14.784	/	/	/	Not estimable
	Pb	0.872	Cap	2	1	50.0	2.045-2.045	/	/	/	Not estimable
			Stem	2	1	50.0	1.020-1.020	/	/	/	Not estimable
	V	0.062	Cap	2	0	100.0	/	/	/	/	Not estimable
			Stem	2	0	100.0	/	/	/	/	Not estimable
	Zn	5.335	Cap	2	2	0.0	59.685-86.924	73.304	66.495-80.114	/	Observed median
			Stem	2	2	0.0	31.181-44.844	38.012	34.597-41.428	/	Observed median
Agaricus bitorquis	As	0.309	Cap	22	0	100.0	/	/	/	/	Not estimable
			Stem	22	0	100.0	/	/	/	/	Not estimable
	Be	0.185	Cap	22	0	100.0	/	/	/	/	Not estimable
			Stem	22	1	95.5	0.386-0.386	/	/	/	Not estimable

Taxon	Element	LoD	Tissue	N	N detected	Censoring (%)	Detected range	Central estimate	IQR	P95	Method
	Cd	0.081	Cap	22	22	0.0	2.213-6.598	3.878	3.628-4.139	5.880	Observed median
			Stem	22	22	0.0	0.899-3.679	2.068	1.935-2.496	3.071	Observed median
	Co	0.104	Cap	22	3	86.4	0.224-0.233	/	/	/	Not estimable
			Stem	22	3	86.4	0.216-0.268	/	/	/	Not estimable
	Cr	1.012	Cap	22	4	81.8	1.811-18.152	/	/	/	Not estimable
			Stem	22	7	68.2	1.255-17.829	0.262	0.053-1.772	/	Exploratory ROS median
	Cu	2.224	Cap	22	22	0.0	101.072-186.215	114.963	108.567-131.615	173.520	Observed median
			Stem	22	22	0.0	73.833-146.929	93.365	86.015-107.006	125.185	Observed median
	Mn	0.529	Cap	22	22	0.0	6.877-14.158	10.541	9.440-11.716	13.863	Observed median
			Stem	22	22	0.0	3.665-17.154	9.617	8.651-10.713	13.196	Observed median
	Ni	3.400	Cap	22	3	86.4	6.733-20.797	/	/	/	Not estimable
			Stem	22	4	81.8	6.444-14.324	/	/	/	Not estimable
	Pb	0.872	Cap	22	22	0.0	1.806-6.664	2.462	2.154-2.815	3.886	Observed median
			Stem	22	22	0.0	0.988-4.223	1.580	1.447-1.895	3.463	Observed median
	V	0.062	Cap	22	10	54.5	0.084-0.187	0.078	0.057-0.122	/	Exploratory ROS median
			Stem	22	13	40.9	0.063-0.202	0.069	0.041-0.101	0.198	ROS median
	Zn	5.335	Cap	22	22	0.0	64.254-129.948	75.369	73.502-83.306	103.112	Observed median
			Stem	22	22	0.0	39.344-97.067	47.549	44.561-52.823	67.921	Observed median
Agaricus nivescens	As	0.309	Cap	1	0	100.0	/	/	/	/	Not estimable
			Stem	1	0	100.0	/	/	/	/	Not estimable
	Be	0.185	Cap	1	0	100.0	/	/	/	/	Not estimable
			Stem	1	0	100.0	/	/	/	/	Not estimable
	Cd	0.081	Cap	1	1	0.0	0.710-0.710	0.710	0.710-0.710	/	Observed median
			Stem	1	1	0.0	0.347-0.347	0.347	0.347-0.347	/	Observed median
	Co	0.104	Cap	1	0	100.0	/	/	/	/	Not estimable
			Stem	1	0	100.0	/	/	/	/	Not estimable

Taxon	Element	LoD	Tissue	N	N detected	Censoring (%)	Detected range	Central estimate	IQR	P95	Method
	Cr	1.012	Cap	1	0	100.0	/	/	/	/	Not estimable
			Stem	1	0	100.0	/	/	/	/	Not estimable
	Cu	2.224	Cap	1	1	0.0	122.999-122.999	122.999	122.999-122.999	/	Observed median
			Stem	1	1	0.0	74.905-74.905	74.905	74.905-74.905	/	Observed median
	Mn	0.529	Cap	1	1	0.0	8.960-8.960	8.960	8.960-8.960	/	Observed median
			Stem	1	1	0.0	9.320-9.320	9.320	9.320-9.320	/	Observed median
	Ni	3.400	Cap	1	0	100.0	/	/	/	/	Not estimable
			Stem	1	0	100.0	/	/	/	/	Not estimable
	Pb	0.872	Cap	1	1	0.0	6.790-6.790	6.790	6.790-6.790	/	Observed median
			Stem	1	0	100.0	/	/	/	/	Not estimable
	V	0.062	Cap	1	0	100.0	/	/	/	/	Not estimable
			Stem	1	0	100.0	/	/	/	/	Not estimable
	Zn	5.335	Cap	1	1	0.0	117.345-117.345	117.345	117.345-117.345	/	Observed median
			Stem	1	1	0.0	83.088-83.088	83.088	83.088-83.088	/	Observed median
Armillaria mellea	As	0.309	Cap	3	0	100.0	/	/	/	/	Not estimable
			Stem	3	0	100.0	/	/	/	/	Not estimable
	Be	0.185	Cap	3	0	100.0	/	/	/	/	Not estimable
			Stem	3	0	100.0	/	/	/	/	Not estimable
	Cd	0.081	Cap	3	3	0.0	0.933-5.381	2.525	1.729-3.953	/	Observed median
			Stem	3	3	0.0	0.206-0.638	0.243	0.224-0.440	/	Observed median
	Co	0.104	Cap	3	0	100.0	/	/	/	/	Not estimable
			Stem	3	0	100.0	/	/	/	/	Not estimable
	Cr	1.012	Cap	3	0	100.0	/	/	/	/	Not estimable
			Stem	3	0	100.0	/	/	/	/	Not estimable
	Cu	2.224	Cap	3	3	0.0	10.639-22.991	22.529	16.584-22.760	/	Observed median
			Stem	3	3	0.0	2.715-14.218	5.131	3.923-9.675	/	Observed median
	Mn	0.529	Cap	3	3	0.0	4.312-13.205	6.194	5.253-9.700	/	Observed median

Taxon	Element	LoD	Tissue	N	N detected	Censoring (%)	Detected range	Central estimate	IQR	P95	Method
			Stem	3	3	0.0	4.255-7.747	4.730	4.492-6.238	/	Observed median
	Ni	3.400	Cap	3	2	33.3	4.920-13.747	/	/	/	Not estimable
			Stem	3	0	100.0	/	/	/	/	Not estimable
	Pb	0.872	Cap	3	0	100.0	/	/	/	/	Not estimable
			Stem	3	0	100.0	/	/	/	/	Not estimable
	V	0.062	Cap	3	0	100.0	/	/	/	/	Not estimable
			Stem	3	0	100.0	/	/	/	/	Not estimable
	Zn	5.335	Cap	3	3	0.0	41.362-111.999	48.262	44.812-80.131	/	Observed median
			Stem	3	3	0.0	7.876-24.580	17.578	12.727-21.079	/	Observed median
Coprinopsis atramentaria	As	0.309	Cap	2	2	0.0	0.854-0.863	0.859	0.857-0.861	/	Observed median
			Stem	2	2	0.0	0.968-0.977	0.973	0.970-0.975	/	Observed median
	Be	0.185	Cap	2	0	100.0	/	/	/	/	Not estimable
			Stem	2	0	100.0	/	/	/	/	Not estimable
	Cd	0.081	Cap	2	2	0.0	1.837-2.983	2.410	2.123-2.697	/	Observed median
			Stem	2	2	0.0	0.506-1.640	1.073	0.789-1.356	/	Observed median
	Co	0.104	Cap	2	1	50.0	0.119-0.119	/	/	/	Not estimable
			Stem	2	1	50.0	0.115-0.115	/	/	/	Not estimable
	Cr	1.012	Cap	2	0	100.0	/	/	/	/	Not estimable
			Stem	2	0	100.0	/	/	/	/	Not estimable
	Cu	2.224	Cap	2	2	0.0	198.930-366.376	282.653	240.792-324.514	/	Observed median
			Stem	2	2	0.0	164.560-294.785	229.672	197.116-262.229	/	Observed median
	Mn	0.529	Cap	2	2	0.0	14.886-22.755	18.820	16.853-20.788	/	Observed median
			Stem	2	2	0.0	10.557-17.728	14.143	12.350-15.935	/	Observed median
	Ni	3.400	Cap	2	2	0.0	4.251-5.682	4.966	4.608-5.324	/	Observed median
			Stem	2	0	100.0	/	/	/	/	Not estimable
	Pb	0.872	Cap	2	2	0.0	5.075-9.735	7.405	6.240-8.570	/	Observed median

Taxon	Element	LoD	Tissue	N	N detected	Censoring (%)	Detected range	Central estimate	IQR	P95	Method
			Stem	2	2	0.0	1.866-5.544	3.705	2.785-4.624	/	Observed median
	V	0.062	Cap	2	2	0.0	0.182-0.320	0.251	0.217-0.286	/	Observed median
			Stem	2	2	0.0	0.188-0.253	0.221	0.204-0.237	/	Observed median
	Zn	5.335	Cap	2	2	0.0	147.567-173.091	160.329	153.948-166.710	/	Observed median
			Stem	2	2	0.0	80.477-125.712	103.094	91.785-114.403	/	Observed median
Macrolepiota procera	As	0.309	Stem	3	1	66.7	1.535-1.535	/	/	/	Not estimable
	Be	0.185	Stem	3	0	100.0	/	/	/	/	Not estimable
	Cd	0.081	Stem	3	3	0.0	0.557-2.703	0.854	0.705-1.779	/	Observed median
	Co	0.104	Stem	3	1	66.7	0.276-0.276	/	/	/	Not estimable
	Cr	1.012	Stem	3	1	66.7	1.184-1.184	/	/	/	Not estimable
	Cu	2.224	Stem	3	3	0.0	11.634-202.536	14.617	13.126-108.577	/	Observed median
	Mn	0.529	Stem	3	3	0.0	6.275-25.420	15.472	10.874-20.446	/	Observed median
	Ni	3.400	Stem	3	2	33.3	10.062-13.095	/	/	/	Not estimable
	Pb	0.872	Stem	3	1	66.7	3.562-3.562	/	/	/	Not estimable
	V	0.062	Stem	3	2	33.3	0.098-0.888	/	/	/	Not estimable
	Zn	5.335	Stem	3	3	0.0	26.847-94.618	51.433	39.140-73.026	/	Observed median
Pholiota adiposa	As	0.309	Cap	1	1	0.0	1.492-1.492	1.492	1.492-1.492	/	Observed median
			Stem	1	0	100.0	/	/	/	/	Not estimable
	Be	0.185	Cap	1	0	100.0	/	/	/	/	Not estimable
			Stem	1	0	100.0	/	/	/	/	Not estimable
	Cd	0.081	Cap	1	1	0.0	2.740-2.740	2.740	2.740-2.740	/	Observed median
			Stem	1	1	0.0	1.773-1.773	1.773	1.773-1.773	/	Observed median
	Co	0.104	Cap	1	1	0.0	0.124-0.124	0.124	0.124-0.124	/	Observed median
			Stem	1	0	100.0	/	/	/	/	Not estimable
	Cr	1.012	Cap	1	1	0.0	1.216-1.216	1.216	1.216-1.216	/	Observed median
			Stem	1	0	100.0	/	/	/	/	Not estimable

Taxon	Element	LoD	Tissue	N	N detected	Censoring (%)	Detected range	Central estimate	IQR	P95	Method
	Cu	2.224	Cap	1	1	0.0	47.796-47.796	47.796	47.796-47.796	/	Observed median
			Stem	1	1	0.0	74.113-74.113	74.113	74.113-74.113	/	Observed median
	Mn	0.529	Cap	1	1	0.0	12.416-12.416	12.416	12.416-12.416	/	Observed median
			Stem	1	1	0.0	6.576-6.576	6.576	6.576-6.576	/	Observed median
	Ni	3.400	Cap	1	1	0.0	10.031-10.031	10.031	10.031-10.031	/	Observed median
			Stem	1	0	100.0	/	/	/	/	Not estimable
	Pb	0.872	Cap	1	0	100.0	/	/	/	/	Not estimable
			Stem	1	1	0.0	2.684-2.684	2.684	2.684-2.684	/	Observed median
	V	0.062	Cap	1	0	100.0	/	/	/	/	Not estimable
			Stem	1	0	100.0	/	/	/	/	Not estimable
	Zn	5.335	Cap	1	1	0.0	57.037-57.037	57.037	57.037-57.037	/	Observed median
			Stem	1	1	0.0	50.114-50.114	50.114	50.114-50.114	/	Observed median
Unknown/Unidentified taxon	As	0.309	Cap	1	1	0.0	4.870-4.870	4.870	4.870-4.870	/	Observed median
	Be	0.185	Cap	1	0	100.0	/	/	/	/	Not estimable
	Cd	0.081	Cap	1	1	0.0	4.780-4.780	4.780	4.780-4.780	/	Observed median
	Co	0.104	Cap	1	0	100.0	/	/	/	/	Not estimable
	Cr	1.012	Cap	1	0	100.0	/	/	/	/	Not estimable
	Cu	2.224	Cap	1	1	0.0	101.260-101.260	101.260	101.260-101.260	/	Observed median
	Mn	0.529	Cap	1	1	0.0	5.382-5.382	5.382	5.382-5.382	/	Observed median
	Ni	3.400	Cap	1	0	100.0	/	/	/	/	Not estimable
	Pb	0.872	Cap	1	1	0.0	6.669-6.669	6.669	6.669-6.669	/	Observed median
	V	0.062	Cap	1	1	0.0	0.160-0.160	0.160	0.160-0.160	/	Observed median
	Zn	5.335	Cap	1	1	0.0	1174.286-1174.286	1174.286	1174.286-1174.286	/	Observed median
Xerocomus porosporus	As	0.309	Cap	1	0	100.0	/	/	/	/	Not estimable
			Stem	1	0	100.0	/	/	/	/	Not estimable

Taxon	Element	LoD	Tissue	N	N detected	Censoring (%)	Detected range	Central estimate	IQR	P95	Method
	Be	0.185	Cap	1	0	100.0	/	/	/	/	Not estimable
			Stem	1	0	100.0	/	/	/	/	Not estimable
	Cd	0.081	Cap	1	1	0.0	2.709-2.709	2.709	2.709-2.709	/	Observed median
			Stem	1	1	0.0	1.191-1.191	1.191	1.191-1.191	/	Observed median
	Co	0.104	Cap	1	0	100.0	/	/	/	/	Not estimable
			Stem	1	0	100.0	/	/	/	/	Not estimable
	Cr	1.012	Cap	1	0	100.0	/	/	/	/	Not estimable
			Stem	1	0	100.0	/	/	/	/	Not estimable
	Cu	2.224	Cap	1	1	0.0	141.620-141.620	141.620	141.620-141.620	/	Observed median
			Stem	1	1	0.0	98.392-98.392	98.392	98.392-98.392	/	Observed median
	Mn	0.529	Cap	1	1	0.0	7.646-7.646	7.646	7.646-7.646	/	Observed median
			Stem	1	1	0.0	5.010-5.010	5.010	5.010-5.010	/	Observed median
	Ni	3.400	Cap	1	0	100.0	/	/	/	/	Not estimable
			Stem	1	0	100.0	/	/	/	/	Not estimable
	Pb	0.872	Cap	1	1	0.0	2.606-2.606	2.606	2.606-2.606	/	Observed median
			Stem	1	1	0.0	1.367-1.367	1.367	1.367-1.367	/	Observed median
	V	0.062	Cap	1	0	100.0	/	/	/	/	Not estimable
			Stem	1	1	0.0	0.064-0.064	0.064	0.064-0.064	/	Observed median
	Zn	5.335	Cap	1	1	0.0	100.243-100.243	100.243	100.243-100.243	/	Observed median
			Stem	1	1	0.0	49.974-49.974	49.974	49.974-49.974	/	Observed median

Concentrations are expressed as mg kg^{-1} dry weight. This table includes only tissue-separated analytical units and excludes whole-fruiting-body records. Most taxa have small n and are descriptive only; *Agaricus bitorquis* is the principal interpretable cap–stem subset. Central estimates are observed medians for uncensored groups or NADA::cenros medians for censored groups with at least three detections and <80% censoring. ROS estimates with ≥50% censoring are labelled exploratory. P95 was reported only when all prespecified eligibility criteria were met: $n \geq 20$, at least 10 detected observations, censoring <50%, availability of a valid observed or convergent ROS distribution, and 2000 valid nonparametric bootstrap estimates. When any criterion was not met, P95 was not reported. Arithmetic and geometric means based on simple LoD substitution are omitted. / = not estimable or not reported.

Table S5. Metal(loid) concentrations by area type using fruiting-body-level units.

Metal	LoD	Area type	*N*	*N* detected	Censoring (%)	Detected range	Central estimate	IQR	P95	Basis
As	0.309	Urban	113	30	73.5	0.326–3.689	0.213	0.083–0.455	/	ROS, exploratory
		Rural	8	7	12.5	0.386–1.364	0.784	0.417–0.929	/	ROS median
Be	0.185	Urban	113	1	99.1	0.285–0.285	/	/	/	Not estimable
		Rural	8	0	100.0	/	/	/	/	Not estimable
Cd	0.081	Urban	113	112	0.9	0.102–101.054	2.034	0.629–2.988	5.460	ROS median
		Rural	8	5	37.5	0.172–2.312	0.183	0.035–0.489	/	ROS median
Co	0.104	Urban	113	28	75.2	0.104–12.745	0.010	0.002–0.062	/	ROS, exploratory
		Rural	8	6	25.0	0.117–1.080	0.150	0.097–0.471	/	ROS median
Cr	1.012	Urban	113	64	43.4	1.109–52.649	1.317	0.464–2.180	9.390	ROS median
		Rural	8	6	25.0	2.033–11.419	4.141	1.835–6.673	/	ROS median
Cu	2.224	Urban	113	113	0.0	7.885–5956.478	70.081	37.391–102.047	191.488	Observed median
		Rural	8	8	0.0	29.042–330.580	80.824	55.135–125.430	/	Observed median
Mn	0.529	Urban	113	113	0.0	2.291–2676.159	12.651	7.916–38.345	364.641	Observed median
		Rural	8	8	0.0	12.068–207.495	40.493	18.361–123.952	/	Observed median
Ni	3.400	Urban	113	56	50.4	3.724–1387.741	2.703	0.859–8.556	/	ROS, exploratory
		Rural	8	8	0.0	3.825–169.724	10.872	7.030–58.873	/	ROS median
Pb	0.872	Urban	113	98	13.3	1.026–41.355	2.106	1.532–3.763	8.104	ROS median
		Rural	8	8	0.0	1.985–10.569	6.621	4.374–8.062	/	Observed median
V	0.062	Urban	113	76	32.7	0.063–15.299	0.096	0.030–0.219	1.018	ROS median

Metal	LoD	Area type	*N*	*N* detected	Censoring (%)	Detected range	Central estimate	IQR	P95	Basis
		Rural	8	8	0.0	0.175–4.338	0.422	0.205–1.693	/	Observed median
Zn	5.335	Urban	113	113	0.0	29.646–3064.543	96.217	64.302–133.969	924.586	Observed median
		Rural	8	8	0.0	87.909–149.401	109.423	100.579–120.859	/	Observed median

Central estimates are observed medians for uncensored groups or medians from the complete NADA::cenros modelled distribution for censored groups with at least three detections and <80% censoring. ROS estimates with ≥50% censoring are exploratory. P95 was reported only when all prespecified eligibility criteria were met: $n \geq 20$, at least 10 detected observations, censoring <50%, availability of a valid observed or convergent ROS distribution, and 2000 valid nonparametric bootstrap estimates. When any criterion was not met, P95 was not reported. Censoring (%) denotes fully censored fruiting-body-level units, and detected ranges include detected observations only. Arithmetic and geometric means based on simple LoD substitution are not reported. / = not estimable/not reported. The rural subset comprised eight fruiting-body-level units, all belonging to *Coprinopsis atramentaria*; therefore, urban–rural differences are completely confounded by taxonomic composition and are presented only as descriptive context, not as independent area-type effects or as a representative rural baseline.

Table S6. Metal(loid) concentrations by urban coordinate quadrant using fruiting-body-level units.

Metal	LoD	Quadrant	N	N detected	Censoring (%)	Detected range	Central estimate	IQR	P95	Basis
As	0.309	NE	30	8	73.3	0.326–2.861	0.065	0.021–0.289	/	ROS, exploratory
		NW	51	20	60.8	0.623–3.689	0.713	0.431–1.348	/	ROS, exploratory
		SE	27	2	92.6	0.419–1.881	/	/	/	Not estimable
		SW	5	0	100.0	/	/	/	/	Not estimable
Be	0.185	NE	30	0	100.0	/	/	/	/	Not estimable
		NW	51	1	98.0	0.285–0.285	/	/	/	Not estimable
		SE	27	0	100.0	/	/	/	/	Not estimable
		SW	5	0	100.0	/	/	/	/	Not estimable
Cd	0.081	NE	30	30	0.0	0.122–101.054	2.046	0.605–2.860	22.351	Observed median
		NW	51	51	0.0	0.102–8.876	2.826	1.849–3.458	5.065	Observed median
		SE	27	27	0.0	0.183–2.793	0.573	0.451–0.756	2.076	Observed median
		SW	5	4	20.0	1.481–13.446	4.814	1.481–5.685	/	ROS median
Co	0.104	NE	30	9	70.0	0.104–12.745	0.005	0.000–0.112	/	ROS, exploratory
		NW	51	10	80.4	0.114–0.640	/	/	/	Not estimable
		SE	27	9	66.7	0.112–11.943	0.012	0.002–0.158	/	ROS, exploratory
		SW	5	0	100.0	/	/	/	/	Not estimable
Cr	1.012	NE	30	13	56.7	1.306–52.649	0.460	0.130–1.698	/	ROS, exploratory
		NW	51	30	41.2	1.109–17.920	1.356	0.484–2.202	9.458	ROS median
		SE	27	19	29.6	1.194–10.656	1.815	0.854–2.638	5.717	ROS median
		SW	5	2	60.0	1.543–2.680	/	/	/	Not estimable
Cu	2.224	NE	30	30	0.0	19.747–460.309	52.952	30.299–84.575	164.560	Observed median

Metal	LoD	Quadrant	N	N detected	Censoring (%)	Detected range	Central estimate	IQR	P95	Basis
		NW	51	51	0.0	15.767–194.022	96.186	60.371–106.308	131.869	Observed median
		SE	27	27	0.0	7.885–5956.478	45.143	35.548–92.402	684.470	Observed median
		SW	5	5	0.0	37.391–129.366	79.357	57.919–80.361	/	Observed median
Mn	0.529	NE	30	30	0.0	2.934–2676.159	255.451	17.519–339.358	838.565	Observed median
		NW	51	51	0.0	2.291–335.834	9.299	5.968–11.428	26.794	Observed median
		SE	27	27	0.0	4.284–217.163	28.512	22.851–31.354	46.105	Observed median
		SW	5	5	0.0	6.474–76.644	9.246	7.164–11.982	/	Observed median
Ni	3.400	NE	30	12	60.0	3.874–68.568	1.966	0.553–8.303	/	ROS, exploratory
		NW	51	17	66.7	3.724–25.921	2.142	1.013–4.756	/	ROS, exploratory
		SE	27	22	18.5	3.778–1387.741	8.556	4.024–16.220	68.780	ROS median
		SW	5	5	0.0	4.387–8.311	6.214	5.029–7.006	/	Observed median
Pb	0.872	NE	30	29	3.3	1.250–8.669	2.677	1.928–4.368	7.918	ROS median
		NW	51	44	13.7	1.026–41.355	1.987	1.540–2.515	5.377	ROS median
		SE	27	20	25.9	1.036–20.689	2.415	0.887–4.267	16.724	ROS median
		SW	5	5	0.0	1.054–7.829	2.869	2.441–4.507	/	Observed median
V	0.062	NE	30	15	50.0	0.063–15.299	0.053	0.008–0.385	/	ROS, exploratory
		NW	51	35	31.4	0.063–0.809	0.084	0.036–0.115	0.732	ROS median
		SE	27	23	14.8	0.065–5.939	0.188	0.120–0.410	1.320	ROS median
		SW	5	3	40.0	0.132–0.261	0.132	0.068–0.138	/	ROS median
Zn	5.335	NE	30	30	0.0	49.669–3064.543	74.781	60.553–97.360	647.506	Observed median
		NW	51	51	0.0	41.385–1375.726	75.108	61.382–286.223	968.125	Observed median
		SE	27	27	0.0	29.646–295.003	109.953	99.098–121.508	181.176	Observed median
		SW	5	5	0.0	113.131–161.629	133.155	125.901–141.004	/	Observed median

Quadrant summaries include only urban fruiting-body-level units assigned to NE, NW, SE or SW. Central estimates and censoring rules follow the criteria described for Table S5. P95 was reported only when all prespecified eligibility criteria were met: $n \geq 20$, at least 10 detected observations, censoring <50%, availability of a valid observed or convergent ROS distribution, and 2000 valid nonparametric bootstrap estimates. When any criterion was not met, P95 was not reported. Quadrant comparisons are descriptive and strongly confounded by taxonomic composition: the SE subset was dominated by *Mycena citrinomarginata*, all SW units belonged to *Panaeolina foenisecii*, and selected upper-tail values in the NE and SE subsets reflected individual taxa or observations. These results should not be interpreted as independent spatial gradients. / = not estimable/not reported.

Table S7. PCA reporting summary and sensitivity analyses for the reconstructed fruiting-body-level mushroom dataset.

Analysis	Use	Elements included	*N*	PC1 (%)	PC2 (%)	PC3 (%)	PC1–PC3 (%)	Dominant PC1 loadings	Dominant PC2 loadings	Dominant PC3 loadings
Primary six-element PCA	Main Figure 4	Cd, Cu, Mn, Pb, V and Zn	121	32.92	25.31	15.69	73.92	Pb (+0.588); V (+0.551); Mn (+0.503)	Zn (+0.566); Cu (+0.540); Cd (+0.507)	Cd (+0.757); Mn (+0.440); V (−0.314)
Broader PCA excluding Be	Supplementary sensitivity	As, Cd, Co, Cr, Cu, Mn, Ni, Pb, V and Zn	121	36.53	19.11	11.40	67.04	Co (−0.444); Cr (−0.429); Ni (−0.417); V (−0.384)	As (−0.562); Zn (−0.550); Mn (+0.416)	Cd (+0.783); As (−0.319); Cu (+0.289)
Full PCA including Be	Diagnostic only	As, Be, Cd, Co, Cr, Cu, Mn, Ni, Pb, V and Zn	121	33.20	17.37	10.91	61.48	Co (+0.445); Cr (+0.435); Ni (+0.414); V (+0.382)	As (+0.563); Zn (+0.553); Mn (−0.413)	Be (+0.640); Cu (+0.411); V (−0.329)

Analysis	N	PC1 (%)	PC2 (%)	PC3 (%)	PC1–PC3 (%)	Procrustes correlation vs primary	Loading congruence PC1; PC2; PC3	Dominant loading order PC1 / PC2 / PC3	Stability interpretation
Primary LoD-capped	121	32.92	25.31	15.69	73.92	1.000	1.000; 1.000; 1.000	Pb > V > Mn / Zn > Cu > Cd / Cd > Mn > V	Reference; stable
LoD/2	121	32.08	25.03	16.41	73.51	0.993	0.998; 0.999; 0.989	Pb > V > Mn / Zn > Cu > Cd / Cd > Mn > V	Stable
ROS-imputed	121	32.31	25.62	15.91	73.84	0.985	0.987; 0.986; 0.998	Pb > V > Mn / Zn > Cu > Cd / Cd > Mn > V	Stable
Exclude FB070	120	33.02	26.47	15.57	75.06	0.998	0.999; 0.998; 0.989	Pb > V > Mn / Cu > Zn > Cd / Cd > Mn > V	Stable
Exclude FB025	120	31.82	24.31	17.16	73.29	0.992	0.866; 0.860; 0.955	Mn > V > Pb / Cu > Zn > Cd / Cd > Mn > V	Stable overall; moderate PC1/PC2 loading sensitivity
Exclude FB070 and FB025	119	31.92	25.30	17.17	74.38	0.990	0.831; 0.823; 0.947	Mn > V > Pb / Cu > Zn > Pb / Cd > Mn > V	Moderately stable

Panel A. Primary, supplementary and diagnostic PCA reporting summary.

The primary PCA was restricted to Cd, Cu, Mn, Pb, V and Zn because these elements provided the most interpretable combination of detection frequency and analytical reliability. The broader ten-element PCA excluding Be was retained as a supplementary sensitivity analysis; loadings for As, Co, Cr and Ni were not used as primary biological evidence because these elements were more strongly affected by censoring and/or analytical uncertainty. The full PCA including Be was retained for diagnostic purposes only because Be was 99.2% censored. Concentrations were log10-transformed, centred and scaled to unit variance before PCA. Loading signs are orientation-dependent; interpretation should focus on loading magnitude and element grouping rather than sign alone.

Panel B. Sensitivity of the primary six-element PCA to censored-value treatment and exclusion of flagged extreme records.

The primary LoD-capped PCA was the reference solution. Procrustes correlation quantifies agreement between score configurations after optimal geometric alignment; values closer to 1 indicate greater similarity. Loading congruence values compare the aligned loading vectors for each component. Across 100 censored-value imputations, the dominant loading structure was retained. In 2000 bootstrap replicates, the strongest contributors were Pb, V and Mn to PC1; Cd, Cu and Zn to PC2; and Cd and Mn to PC3. FB025 was the multielement-extreme *Pholiota adiposa* unit, whereas FB070 was the Cu-extreme *Mycena citrinomarginata* unit. Both records were retained in the primary analysis because no objective analytical exclusion criterion was available.

Supplementary Table S8. Primary and exploratory park-level soil–mushroom and soil-physicochemical correlations based on the harmonised eight-park dataset.

Element	Overlapping parks (n)	Spearman ρ	Exact permutation p	BH-FDR across six primary tests	Leave-one-out ρ minimum	Leave-one-out ρ maximum	Sign change
Cd	8	0.714	0.058	0.346	0.571	0.857	No
Cu	8	0.238	0.582	0.699	-0.071	0.393	Yes
Mn	8	-0.429	0.299	0.449	-0.714	-0.143	No
Pb	8	0.431	0.285	0.449	0.179	0.721	No
V	8	0.156	0.716	0.716	-0.143	0.607	Yes
Zn	8	0.595	0.132	0.397	0.393	0.821	No

Soil variable	Mushroom element	Parks (n)	Spearman ρ	Exact p	FDR within family	Global FDR	LOO ρ min.	LOO ρ max.	Sign change
Be	As	8	0.109	0.786	0.954	0.967	-0.612	0.612	Yes
	Cd	8	0.643	0.096	0.670	0.753	0.464	0.857	No
	Co	8	-0.436	0.321	0.862	0.916	-0.612	0.000	No
	Cr	8	-0.366	0.379	0.862	0.916	-0.559	-0.037	No
	Cu	8	0.143	0.752	0.954	0.967	0.036	0.714	No
	Mn	8	-0.833	0.015	0.513	0.554	-0.964	-0.750	No
	Ni	8	-0.390	0.337	0.862	0.916	-0.667	-0.185	No
	Pb	8	-0.647	0.092	0.670	0.753	-0.929	-0.468	No
	V	8	-0.108	0.806	0.954	0.967	-0.393	0.342	Yes
	Zn	8	0.548	0.171	0.819	0.846	0.321	0.786	No
V	As	8	0.343	0.393	0.862	0.916	0.204	0.579	No
	Cd	8	-0.190	0.665	0.942	0.967	-0.357	0.143	Yes
	Co	8	0.078	0.857	0.954	0.967	-0.408	0.408	Yes
	Cr	8	-0.220	0.617	0.938	0.967	-0.630	0.072	Yes
	Cu	8	0.238	0.582	0.938	0.967	0.107	0.857	No
	Mn	8	-0.381	0.360	0.862	0.916	-0.679	-0.071	No
	Ni	8	0.220	0.617	0.938	0.967	-0.054	0.519	Yes
	Pb	8	-0.240	0.564	0.938	0.967	-0.631	0.071	Yes
	Zn	8	0.119	0.793	0.954	0.967	-0.071	0.679	Yes
Cr	As	8	0.078	0.857	0.954	0.967	-0.408	0.408	Yes

Element	Overlapping parks (n)	Spearman ρ	Exact permutation p	BH-FDR across six primary tests	Leave-one-out ρ minimum	Leave-one-out ρ maximum	Sign change		
	Cd	8	0.881	0.007	0.513	0.554	0.821	0.929	No
	Co	8	-0.187	0.679	0.942	0.967	-0.408	0.204	Yes
	Cr	8	-0.366	0.379	0.612	0.916	-0.593	-0.111	No
	Cu	8	0.071	0.882	0.954	0.967	-0.071	0.464	Yes
	Mn	8	-0.524	0.197	0.819	0.846	-0.679	-0.393	No
	Ni	8	-0.439	0.279	0.862	0.904	-0.704	-0.148	No
	Pb	8	-0.359	0.379	0.862	0.916	-0.571	-0.108	No
	V	8	-0.180	0.672	0.942	0.967	-0.306	0.198	Yes
	Zn	8	0.357	0.389	0.862	0.916	0.071	0.571	No
Mn	As	8	0.218	0.607	0.938	0.967	-0.408	0.612	Yes
	Cd	8	0.524	0.197	0.819	0.846	0.321	0.714	No
	Co	8	-0.296	0.500	0.938	0.967	-0.535	0.000	No
	Cr	8	-0.317	0.449	0.898	0.951	-0.450	-0.037	No
	Cu	8	-0.524	0.197	0.819	0.846	-0.750	-0.429	No
	Ni	8	-0.708	0.064	0.631	0.701	-0.937	-0.556	No
	Pb	8	-0.455	0.258	0.861	0.877	-0.679	-0.252	No
	V	8	-0.707	0.060	0.631	0.701	-0.847	-0.595	No
	Zn	8	-0.190	0.665	0.942	0.967	-0.786	-0.036	No
Co	As	8	0.109	0.786	0.954	0.967	-0.612	0.612	Yes
	Cd	8	0.714	0.058	0.631	0.701	0.571	0.929	No
	Co	8	-0.327	0.429	0.612	0.936	-0.612	0.204	Yes
	Cr	8	-0.342	0.410	0.873	0.934	-0.519	0.000	No
	Cu	8	-0.071	0.882	0.954	0.967	-0.214	0.393	Yes
	Mn	8	-0.786	0.028	0.559	0.701	-0.857	-0.679	No
	Ni	8	-0.317	0.449	0.898	0.951	-0.595	-0.037	No
	Pb	8	-0.611	0.118	0.694	0.753	-0.750	-0.414	No
	V	8	-0.120	0.779	0.954	0.967	-0.393	0.324	Yes
	Zn	8	0.476	0.243	0.861	0.877	0.214	0.679	No
Ni	As	8	-0.031	0.964	1.000	1.000	-0.612	0.408	Yes
	Cd	8	0.714	0.058	0.631	0.701	0.571	0.929	No
	Co	8	-0.109	0.786	0.954	0.967	-0.612	0.612	Yes
	Cr	8	-0.171	0.700	0.954	0.967	-0.519	0.259	Yes

Element	Overlapping parks (n)	Spearman ρ	Exact permutation p	BH-FDR across six primary tests	Leave-one-out ρ minimum	Leave-one-out ρ maximum	Sign change		
	Cu	8	-0.214	0.619	0.938	0.967	-0.464	0.179	Yes
	Mn	8	-0.500	0.216	0.831	0.846	-0.643	-0.357	No
	Ni	8	-0.464	0.255	0.598	0.877	-0.667	-0.222	No
	Pb	8	-0.455	0.258	0.861	0.877	-0.714	-0.180	No
	V	8	-0.252	0.547	0.938	0.967	-0.414	0.126	Yes
	Zn	8	0.190	0.665	0.942	0.967	-0.036	0.464	Yes
Cu	As	8	0.436	0.321	0.862	0.916	0.000	0.612	No
	Cd	8	0.357	0.389	0.862	0.916	0.071	0.571	No
	Co	8	0.094	0.821	0.954	0.967	-0.045	0.267	Yes
	Cr	8	0.244	0.564	0.938	0.967	0.072	0.559	No
	Mn	8	-0.429	0.299	0.862	0.904	-0.643	-0.143	No
	Ni	8	0.244	0.564	0.938	0.967	0.018	0.649	No
	Pb	8	-0.060	0.897	0.954	0.967	-0.357	0.306	Yes
	V	8	0.539	0.176	0.819	0.846	0.306	0.667	No
	Zn	8	0.833	0.015	0.513	0.554	0.750	0.964	No
Zn	As	8	0.109	0.786	0.954	0.967	-0.612	0.612	Yes
	Cd	8	0.690	0.069	0.631	0.701	0.536	0.893	No
	Co	8	-0.327	0.429	0.893	0.936	-0.612	0.204	Yes
	Cr	8	-0.244	0.564	0.938	0.967	-0.408	0.148	Yes
	Cu	8	0.000	1.000	1.000	1.000	-0.179	0.500	Yes
	Mn	8	-0.714	0.058	0.631	0.701	-0.857	-0.571	No
	Ni	8	-0.220	0.617	0.938	0.967	-0.450	0.074	Yes
	Pb	8	-0.503	0.209	0.831	0.846	-0.750	-0.252	No
	V	8	0.012	0.990	1.000	1.000	-0.214	0.523	Yes
As	As	8	0.296	0.500	0.625	0.967	0.000	0.535	No
	Cd	8	-0.024	0.977	1.000	1.000	-0.214	0.214	Yes
	Co	8	-0.452	0.286	0.862	0.904	-0.612	-0.204	No
	Cr	8	-0.195	0.652	0.942	0.967	-0.450	0.111	Yes
	Cu	8	-0.357	0.389	0.862	0.916	-0.786	-0.036	No
	Mn	8	-0.095	0.840	0.954	0.967	-0.321	0.286	Yes
	Ni	8	-0.220	0.617	0.938	0.967	-0.523	-0.036	No
	Pb	8	-0.072	0.872	0.954	0.967	-0.393	0.286	Yes

Element	Overlapping parks (n)	Spearman ρ	Exact permutation p	BH-FDR across six primary tests	Leave-one-out ρ minimum	Leave-one-out ρ maximum	Sign change		
	V	8	-0.407	0.315	0.862	0.916	-0.613	-0.143	No
	Zn	8	-0.095	0.840	0.954	0.967	-0.393	0.357	Yes
Cd	As	8	0.109	0.786	0.954	0.967	-0.612	0.612	Yes
	Co	8	-0.218	0.607	0.938	0.967	-0.612	0.408	Yes
	Cr	8	-0.220	0.617	0.938	0.967	-0.519	0.185	Yes
	Cu	8	-0.119	0.793	0.954	0.967	-0.321	0.321	Yes
	Mn	8	-0.643	0.096	0.670	0.753	-0.786	-0.464	No
	Ni	8	-0.634	0.104	0.670	0.753	-0.778	-0.482	No
	Pb	8	-0.563	0.155	0.814	0.795	-0.857	-0.342	No
	V	8	-0.347	0.397	0.862	0.916	-0.536	-0.018	No
	Zn	8	0.238	0.582	0.938	0.967	-0.143	0.429	Yes
Pb	As	8	0.655	0.107	0.670	0.753	0.408	0.802	No
	Cd	8	0.476	0.243	0.861	0.877	0.250	0.750	No
	Co	8	-0.062	0.893	0.954	0.967	-0.408	0.204	Yes
	Cr	8	0.146	0.743	0.954	0.967	-0.144	0.396	Yes
	Cu	8	0.595	0.132	0.735	0.753	0.393	0.821	No
	Mn	8	-0.024	0.977	1.000	1.000	-0.214	0.464	Yes
	Ni	8	0.000	1.000	1.000	1.000	-0.148	0.360	Yes
	V	8	0.383	0.345	0.862	0.916	0.143	0.541	No
	Zn	8	0.810	0.022	0.544	0.653	0.714	0.964	No
pH	As	8	-0.062	0.893	0.977	0.967	-0.408	0.204	Yes
	Cd	8	0.119	0.793	0.977	0.967	-0.071	0.679	Yes
	Co	8	-0.296	0.500	0.977	0.967	-0.535	0.000	No
	Cr	8	-0.171	0.700	0.977	0.967	-0.595	0.037	Yes
	Cu	8	-0.690	0.069	0.744	0.701	-0.714	-0.607	No
	Mn	8	-0.048	0.935	0.977	0.984	-0.571	0.107	Yes
	Ni	8	-0.708	0.064	0.744	0.701	-0.793	-0.649	No
	Pb	8	-0.299	0.468	0.977	0.967	-0.739	-0.054	No
	V	8	-0.850	0.012	0.403	0.554	-0.883	-0.786	No
	Zn	8	-0.595	0.132	0.744	0.753	-0.929	-0.429	No
EC	As	8	0.094	0.821	0.977	0.967	-0.045	0.267	Yes
	Cd	8	-0.214	0.619	0.977	0.967	-0.643	-0.036	No

Element	Overlapping parks (n)	Spearman ρ	Exact permutation p	BH-FDR across six primary tests	Leave-one-out ρ minimum	Leave-one-out ρ maximum	Sign change		
	Co	8	0.483	0.250	0.875	0.877	0.408	0.757	No
	Cr	8	0.073	0.887	0.977	0.967	-0.148	0.450	Yes
	Cu	8	0.143	0.752	0.977	0.967	-0.214	0.429	Yes
	Mn	8	-0.286	0.501	0.977	0.967	-0.500	-0.071	No
	Ni	8	-0.366	0.379	0.977	0.916	-0.556	-0.037	No
	Pb	8	-0.419	0.301	0.959	0.904	-0.757	-0.179	No
	V	8	-0.192	0.647	0.977	0.967	-0.505	0.036	Yes
	Zn	8	-0.381	0.360	0.977	0.916	-0.607	-0.214	No
OM	As	8	0.016	1.000	1.000	1.000	-0.134	0.178	Yes
	Cd	8	0.429	0.299	0.959	0.904	0.143	0.750	No
	Co	8	0.094	0.821	0.977	0.967	-0.045	0.267	Yes
	Cr	8	-0.171	0.700	0.977	0.967	-0.450	0.126	Yes
	Cu	8	0.500	0.216	0.841	0.846	0.321	0.714	No
	Mn	8	-0.500	0.216	0.841	0.846	-0.714	-0.250	No
	Ni	8	0.049	0.928	0.977	0.983	-0.252	0.252	Yes
	Pb	8	-0.299	0.468	0.977	0.967	-0.643	-0.054	No
	V	8	0.347	0.397	0.977	0.916	0.036	0.523	No
	Zn	8	0.524	0.197	0.841	0.846	0.321	0.643	No
Humidity	As	8	0.156	0.750	0.977	0.967	0.000	0.356	No
	Cd	8	0.500	0.216	0.841	0.846	0.250	0.821	No
	Co	8	-0.016	1.000	1.000	1.000	-0.178	0.134	Yes
	Cr	8	-0.220	0.617	0.977	0.967	-0.523	0.054	Yes
	Cu	8	0.571	0.151	0.756	0.795	0.429	0.786	No
	Mn	8	-0.595	0.132	0.744	0.753	-0.821	-0.393	No
	Ni	8	-0.024	0.979	1.000	1.000	-0.342	0.162	Yes
	Pb	8	-0.323	0.432	0.977	0.936	-0.679	-0.090	No
	V	8	0.323	0.432	0.977	0.936	0.000	0.487	No
	Zn	8	0.643	0.096	0.744	0.753	0.500	0.786	No
Clay	As	8	-0.187	0.679	0.977	0.967	-0.408	0.204	Yes
	Cd	8	-0.214	0.619	0.977	0.967	-0.821	-0.107	No
	Co	8	0.047	0.929	0.977	0.983	-0.204	0.223	Yes
	Cr	8	-0.268	0.528	0.977	0.967	-0.482	0.018	Yes

Element	Overlapping parks (n)	Spearman ρ	Exact permutation p	BH-FDR across six primary tests	Leave-one-out ρ minimum	Leave-one-out ρ maximum	Sign change		
	Cu	8	0.190	0.665	0.977	0.967	0.000	0.607	No
	Mn	8	-0.095	0.840	0.977	0.967	-0.393	0.357	Yes
	Ni	8	0.610	0.122	0.744	0.753	0.468	0.741	No
	Pb	8	-0.060	0.897	0.977	0.967	-0.252	0.234	Yes
	V	8	0.383	0.345	0.977	0.916	0.179	0.786	No
	Zn	8	0.095	0.840	0.977	0.967	-0.143	0.429	Yes
Sand	As	8	-0.063	0.893	0.977	0.967	-0.412	0.206	Yes
	Cd	8	0.132	0.761	0.977	0.967	-0.054	0.703	Yes
	Co	8	-0.078	0.875	0.977	0.967	-0.412	0.412	Yes
	Cr	8	0.123	0.774	0.977	0.967	-0.209	0.486	Yes
	Cu	8	-0.683	0.070	0.744	0.701	-0.775	-0.595	No
	Mn	8	0.096	0.825	0.977	0.967	-0.360	0.288	Yes
	Ni	8	-0.589	0.134	0.744	0.753	-0.721	-0.482	No
	Pb	8	-0.127	0.762	0.977	0.967	-0.482	0.209	Yes
	V	8	-0.639	0.095	0.744	0.753	-0.709	-0.505	No
	Zn	8	-0.479	0.233	0.857	0.877	-0.829	-0.324	No
Silt	As	8	0.166	0.768	0.977	0.967	0.045	0.363	No
	Cd	8	0.217	0.605	0.977	0.967	-0.182	0.436	Yes
	Co	8	0.047	0.893	0.977	0.967	-0.208	0.227	Yes
	Cr	8	-0.111	0.802	0.977	0.967	-0.400	0.211	Yes
	Cu	8	0.855	0.010	0.403	0.554	0.793	0.891	No
	Mn	8	-0.265	0.523	0.977	0.967	-0.468	0.018	Yes
	Ni	8	0.272	0.506	0.977	0.967	0.094	0.587	No
	Pb	8	0.042	0.927	0.977	0.983	-0.234	0.327	Yes
	V	8	0.582	0.138	0.744	0.754	0.378	0.807	No
	Zn	8	0.687	0.067	0.744	0.701	0.527	0.847	No

Panel A. Primary same-element correlations.

The primary panel comprised the six prespecified same-element correlations for Cd, Cu, Mn, Pb, V and Zn. Exact p-values were obtained by enumeration, and the Benjamini–Hochberg false-discovery-rate adjustment was applied across these six tests. The leave-one-out range is based on eight omissions, each retaining seven parks. None of the six primary associations remained statistically significant after adjustment.

Panel B. Broader exploratory correlations.

Panel B contains the remaining 174 correlations from the complete 180-test exploratory matrix; the six prespecified primary correlations shown in Panel A are not repeated. Within-family FDR values were calculated separately for same-element, cross-element and soil-property–mushroom analyses, whereas global FDR values were calculated across the complete 180-test exploratory matrix. The fixed overlap comprised Abbey Park, Bradgate Park, Braunstone Park, Castle Gardens, Knighton Park, Stokes Wood Park, Victoria Park and Western Park. Mushroom values were summarised at park level using the predefined observed-median or ROS rules, and soil values represented homogenised site-level composites. All associations are contextual park-level analyses and should not be interpreted as prediction, mechanistic transfer or individual uptake. None remained statistically significant after the applicable within-family or global FDR adjustment. LOO, leave-one-park-out; ROS, regression on order statistics.

Supplementary Table S9. Leave-one-park-out sensitivity analysis of the primary same-element park-level correlations based on the harmonised eight-park dataset.

Element	Full eight-park ρ	LOO ρ minimum	LOO ρ maximum	Any sign change	Park causing largest change	Largest absolute change
Cd	0.714	0.571	0.857	No	Bradgate Park	0.143
Cu	0.238	-0.071	0.393	Yes	Knighton Park	0.310
Mn	-0.429	-0.714	-0.143	No	Abbey Park	0.286
Pb	0.431	0.179	0.721	No	Castle Gardens	0.290
V	0.156	-0.143	0.607	Yes	Knighton Park	0.451
Zn	0.595	0.393	0.821	No	Bradgate Park	0.226

Element	Omitted park	Remaining parks (n)	Full eight-park ρ	Leave-one-out ρ	Absolute change	Sign changed
Cd	Abbey Park	7	0.714	0.750	0.036	No
	Bradgate Park	7	0.714	0.571	0.143	No
	Braunstone Park	7	0.714	0.857	0.143	No
	Castle Gardens	7	0.714	0.679	0.036	No
	Knighton Park	7	0.714	0.571	0.143	No
	Stokes Wood Park	7	0.714	0.821	0.107	No
	Victoria Park	7	0.714	0.571	0.143	No
	Western Park	7	0.714	0.821	0.107	No
Cu	Abbey Park	7	0.238	0.071	0.167	No
	Bradgate Park	7	0.238	0.393	0.155	No
	Braunstone Park	7	0.238	0.214	0.024	No
	Castle Gardens	7	0.238	0.214	0.024	No
	Knighton Park	7	0.238	-0.071	0.310	Yes
	Stokes Wood Park	7	0.238	0.393	0.155	No
	Victoria Park	7	0.238	0.250	0.012	No
	Western Park	7	0.238	0.393	0.155	No
Mn	Abbey Park	7	-0.429	-0.714	0.286	No
	Bradgate Park	7	-0.429	-0.357	0.071	No
	Braunstone Park	7	-0.429	-0.464	0.036	No
	Castle Gardens	7	-0.429	-0.143	0.286	No
	Knighton Park	7	-0.429	-0.286	0.143	No
	Stokes Wood Park	7	-0.429	-0.464	0.036	No
	Victoria Park	7	-0.429	-0.357	0.071	No
	Western Park	7	-0.429	-0.571	0.143	No

Element	Full eight-park ρ	LOO ρ minimum	LOO ρ maximum	Any sign change	Park causing largest change	Largest absolute change
Pb	Abbey Park	7	0.431	0.432	0.001	No
	Bradgate Park	7	0.431	0.432	0.001	No
	Braunstone Park	7	0.431	0.541	0.109	No
	Castle Gardens	7	0.431	0.721	0.290	No
	Knighton Park	7	0.431	0.179	0.253	No
	Stokes Wood Park	7	0.431	0.414	0.017	No
	Victoria Park	7	0.431	0.432	0.001	No
	Western Park	7	0.431	0.250	0.181	No
V	Abbey Park	7	0.156	-0.143	0.299	Yes
	Bradgate Park	7	0.156	-0.036	0.192	Yes
	Braunstone Park	7	0.156	0.072	0.084	No
	Castle Gardens	7	0.156	0.180	0.024	No
	Knighton Park	7	0.156	0.607	0.451	No
	Stokes Wood Park	7	0.156	0.144	0.012	No
	Victoria Park	7	0.156	0.252	0.097	No
	Western Park	7	0.156	0.180	0.024	No
Zn	Abbey Park	7	0.595	0.571	0.024	No
	Bradgate Park	7	0.595	0.821	0.226	No
	Braunstone Park	7	0.595	0.393	0.202	No
	Castle Gardens	7	0.595	0.393	0.202	No
	Knighton Park	7	0.595	0.571	0.024	No
	Stokes Wood Park	7	0.595	0.679	0.083	No
	Victoria Park	7	0.595	0.571	0.024	No
	Western Park	7	0.595	0.679	0.083	No

Panel A. Summary of leave-one-park-out sensitivity**.**

Each primary same-element correlation was recalculated eight times after omitting one park, leaving $n = 7$ parks in each repetition. The summary identifies the observed coefficient range, whether the sign changed relative to the full eight-park estimate, and the omission producing the largest absolute change.

Panel B. Detailed leave-one-park-out results.

Leave-one-park-out coefficients were used as diagnostics of influence and stability only; p-values were not interpreted for the $n = 7$ repetitions. Sign changes occurred for Cu and V under selected omissions, while the magnitude of the coefficients varied for all elements. These results support cautious interpretation of the eight-park correlations because of the small effective sample size and taxonomically unbalanced mushroom sampling.

Table S10. Central and high-end dietary-screening scenarios at 300 g fresh weight week^{-1} and a dry-matter fraction of 0.10.

Screening block	Taxon	Element	N	Central conc. (mg kg^{-1} dw)	Central screening	P95 conc. (mg kg^{-1} dw)	P95 screening	Output
Edible-relevant	*Agaricus bitorquis*	Cd	22	2.968	50.88	4.230	72.52	% TWI
		Cu	22	105.603	9.24	132.024	11.55	% EFSA safe level
		Mn	22	9.983	0.53	12.644	0.68	% EFSA safe level
		Ni	22	1.259	0.59	/	/	% TDI
		Pb	22	2.082	3.92	3.390	2.41	MOE
		V	22	0.083	0.05	0.174	0.11	% MRL-equivalent
		Zn	22	63.363	1.09	84.477	1.45	% UL
Hypothetical ingestion	*Mycena citrinomarginata*	Cd	22	0.556	9.53	1.718	29.46	% TWI
		Cu	22	50.781	4.44	851.426	74.47	% EFSA safe level
		Mn	22	29.032	1.56	47.327	2.54	% EFSA safe level
		Ni	22	11.796	5.56	75.154	35.39	% TDI
		Pb	22	3.012	2.71	19.259	0.42	MOE
		V	22	0.211	0.13	1.410	0.86	% MRL-equivalent
		Zn	22	112.666	1.93	195.722	3.36	% UL
	Panaeolina foenisecii	Cd	16	2.566	43.99	/	/	% TWI

Screening block	Taxon	Element	N	Central conc. (mg kg⁻¹ dw)	Central screening	P95 conc. (mg kg⁻¹ dw)	P95 screening	Output
		Cu	16	57.788	5.05	/	/	% EFSA safe level
		Mn	16	162.809	8.72	/	/	% EFSA safe level
		Ni	16	5.515	2.60	/	/	% TDI
		Pb	16	3.895	2.10	/	/	MOE
		V	16	0.112	0.07	/	/	% MRL-equivalent
		Zn	16	86.031	1.47	/	/	% UL
	Unknown/Unidentified taxon	As	17	1.941	0.50	/	/	MOE
		Cd	17	2.421	41.51	/	/	% TWI
		Cu	17	72.288	6.32	/	/	% EFSA safe level
		Mn	17	5.214	0.28	/	/	% EFSA safe level
		Ni	17	2.002	0.94	/	/	% TDI
		Pb	17	1.641	4.98	/	/	MOE
		V	17	0.100	0.06	/	/	% MRL-equivalent
		Zn	17	475.986	8.16	/	/	% UL

High-end results were reported only when all prespecified P95 eligibility criteria were met: $n \geq 20$, at least 10 detected observations, censoring <50%, availability of a valid observed or convergent ROS distribution, and 2000 valid nonparametric bootstrap estimates. When any criterion was not met, the P95 concentration and screening fields are shown as /. For MOE outputs, a lower value indicates greater potential concern; for percentage outputs, a higher value indicates a larger fraction of the selected benchmark.

Table S11. Sensitivity of the central dietary-screening estimates to assumed dry-matter fractions of 0.08, 0.10 and 0.12.

Taxon	Element	Output	DM = 0.08	DM = 0.10	DM = 0.12
Agaricus arvensis	Cd	% TWI	7.78	9.73	11.67
	Cu	% EFSA safe level	2.26	2.82	3.38
	Mn	% EFSA safe level	0.21	0.26	0.31
	Zn	% UL	0.76	0.95	1.14
Agaricus bitorquis	Cd	% TWI	40.71	50.88	61.06
	Cu	% EFSA safe level	7.39	9.24	11.08
	Mn	% EFSA safe level	0.43	0.53	0.64
	Ni	% TDI	0.47	0.59	0.71
	Pb	MOE	4.90	3.92	3.27
	V	% MRL-equivalent	0.04	0.05	0.06
	Zn	% UL	0.87	1.09	1.30
Agaricus nivescens	Cd	% TWI	7.24	9.05	10.87
	Cu	% EFSA safe level	6.92	8.65	10.39
	Mn	% EFSA safe level	0.39	0.49	0.59
	Zn	% UL	1.37	1.72	2.06
Coprinopsis atramentaria	As	MOE	1.56	1.25	1.04
	Cd	% TWI	2.51	3.14	3.76
	Cu	% EFSA safe level	5.66	7.07	8.48
	Mn	% EFSA safe level	1.74	2.17	2.60
	Ni	% TDI	4.10	5.12	6.14
	Pb	MOE	1.54	1.23	1.03
	V	% MRL-equivalent	0.21	0.26	0.31

Taxon	Element	Output	DM = 0.08	DM = 0.10	DM = 0.12
	Zn	% UL	1.50	1.88	2.25
Leucoagaricus leucothites	Cd	% TWI	15.97	19.96	23.95
	Cu	% EFSA safe level	0.99	1.24	1.49
	Mn	% EFSA safe level	0.32	0.40	0.48
	V	% MRL-equivalent	0.08	0.11	0.13
	Zn	% UL	0.41	0.51	0.61
Marasmius oreades	Cd	% TWI	3.80	4.75	5.70
	Cu	% EFSA safe level	4.39	5.49	6.59
	Mn	% EFSA safe level	0.61	0.77	0.92
	Pb	MOE	8.16	6.53	5.44
	V	% MRL-equivalent	0.09	0.12	0.14
	Zn	% UL	1.05	1.31	1.58
Vascellum pratense	Cd	% TWI	29.37	36.71	44.06
	Cu	% EFSA safe level	4.39	5.49	6.58
	Mn	% EFSA safe level	5.48	6.84	8.21
	Pb	MOE	5.26	4.21	3.51
	Zn	% UL	1.33	1.66	1.99

Results correspond to the 300 g fresh-weight/week scenario. Intake-based percentage outputs change proportionally with the assumed dry-matter fraction (−20% at 0.08 and +20% at 0.12 relative to 0.10). MOE values change inversely (+25% at 0.08 and −16.7% at 0.12).

Table S12. Supplementary hypothetical Cr(VI)-fraction sensitivity analysis.

Screening block	Taxon	*N*	*N* detected	Censoring (%)	Total Cr central estimate (mg kg⁻¹ dw)	Assumed Cr(VI) fraction (%)	Cr(VI)-equivalent conc. (mg kg⁻¹ dw)	Weekly intake at 300 g FW (µg)
Edible-relevant	*Agaricus bitorquis*	22	8	63.6	0.318	1.0	0.0032	0.095
		22	8	63.6	0.318	10.0	0.0318	0.953
		22	8	63.6	0.318	16.7	0.0531	1.592
		22	8	63.6	0.318	100.0	0.3178	9.535
	Coprinopsis atramentaria	8	6	25.0	4.141	1.0	0.0414	1.242
		8	6	25.0	4.141	10.0	0.4141	12.422
		8	6	25.0	4.141	16.7	0.6915	20.744
		8	6	25.0	4.141	100.0	4.1405	124.216
Hypothetical ingestion	*Mycena citrinomarginata*	22	19	13.6	1.997	1.0	0.0200	0.599
		22	19	13.6	1.997	10.0	0.1997	5.990
		22	19	13.6	1.997	16.7	0.3335	10.004
		22	19	13.6	1.997	100.0	1.9967	59.902
	Panaeolina foenisecii	16	7	56.2	1.105	1.0	0.0110	0.331
		16	7	56.2	1.105	10.0	0.1105	3.314
		16	7	56.2	1.105	16.7	0.1845	5.534
		16	7	56.2	1.105	100.0	1.1046	33.137

Screening block	Taxon	*N*	*N* detected	Censoring (%)	Total Cr central estimate (mg kg⁻¹ dw)	Assumed Cr(VI) fraction (%)	Cr(VI)-equivalent conc. (mg kg⁻¹ dw)	Weekly intake at 300 g FW (µg)
	Pholiota adiposa	5	3	40.0	1.114	1.0	0.0111	0.334
		5	3	40.0	1.114	10.0	0.1114	3.342
		5	3	40.0	1.114	16.7	0.1860	5.581
		5	3	40.0	1.114	100.0	1.1141	33.422
	Pholiota gummosa	2	2	0.0	4.428	1.0	0.0443	1.328
		2	2	0.0	4.428	10.0	0.4428	13.284
		2	2	0.0	4.428	16.7	0.7395	22.184
		2	2	0.0	4.428	100.0	4.4278	132.835
	Psathyrella corrugis	2	2	0.0	1.874	1.0	0.0187	0.562
		2	2	0.0	1.874	10.0	0.1874	5.623
		2	2	0.0	1.874	16.7	0.3130	9.391
		2	2	0.0	1.874	100.0	1.8745	56.234
	Psathyrella fatua	6	5	16.7	1.638	1.0	0.0164	0.491
		6	5	16.7	1.638	10.0	0.1638	4.913
		6	5	16.7	1.638	16.7	0.2735	8.204
		6	5	16.7	1.638	100.0	1.6375	49.126
	Unknown/Unidentified taxon	17	15	11.8	1.738	1.0	0.0174	0.521

Screening block	Taxon	*N*	*N* detected	Censoring (%)	Total Cr central estimate (mg kg⁻¹ dw)	Assumed Cr(VI) fraction (%)	Cr(VI)-equivalent conc. (mg kg⁻¹ dw)	Weekly intake at 300 g FW (µg)
		17	15	11.8	1.738	10.0	0.1738	5.214
		17	15	11.8	1.738	16.7	0.2903	8.708
		17	15	11.8	1.738	100.0	1.7382	52.145

The assumed fractions of 1%, 10%, 16.7% and 100% are arbitrary sensitivity scenarios and are not measured or literature-derived Cr(VI) proportions. The 16.7% scenario corresponds mathematically to the former total-Cr/6 assumption. These results are excluded from the primary dietary screening and should not be interpreted as evidence that Cr(VI) was present.

References

12. Jagdev, G.S.; Evans, M.D.; Lobo-Bedmar, M.C.; Sgamma, T.; Peña-Fernández, A. Spatial distribution and soil-to-fungus trans-fer of cadmium, copper, and zinc in urban and rural green spaces of Leicestershire, UK. Environments 2026, 13, 312.

13. Peña-Fernández, A.; Cámara-Pastor, T.; Evans, M.D.; Lobo-Bedmar, M.C.; Jagdev, G.S. Metal(loid)s in urban and rural com-posite topsoils from Leicester and Leicestershire, UK: Spatial patterns, enrichment and screening-level health assessment. En-vironmental Geochemistry and Health, 2026, submitted manuscript.

20. EFSA CONTAM Panel. Scientific opinion on cadmium in food. EFSA J. 2009, 7, 980.

21. EFSA CONTAM Panel. Scientific opinion on lead in food. EFSA J. 2010, 8, 1570.

42. EFSA CONTAM Panel. Update of the risk assessment of inorganic arsenic in food. EFSA J. 2024, 22, e8488.

43. EFSA CONTAM Panel. Update of the risk assessment of nickel in food and drinking water. EFSA J. 2020, 18, e6268.

44. EFSA Scientific Committee. Re-evaluation of the existing health-based guidance values for copper and exposure assessment from all sources. EFSA J. 2023, 21, e7728.

45. EFSA NDA Panel. Scientific opinion on the tolerable upper intake level for manganese. EFSA J. 2023, 21, e8413.

46. Agency for Toxic Substances and Disease Registry. Toxicological Profile for Vanadium; ATSDR: Atlanta, GA, USA, 2012.

47. Scientific Committee on Food. Opinion of the Scientific Committee on Food on the Tolerable Upper Intake Level of Zinc; European Commission: Brussels, Belgium, 2003.
